# Supplementary material for: Advancing regulatory variant effect prediction with AlphaGenome
Source: Nature. 2026 Jan 28;649(8099):1206–18. doi: 10.1038/s41586-025-10014-0 (PMC12851941; doi:10.1038/s41586-025-10014-0)
Supplement: Supplementary file 1 — This file contains Supplementary Methods (detailed description of data acquisition and processing, model architecture, training procedures (pretraining and distillation) and benchmarking protocols against existing methods), figures, notes and references. [file 41586_2025_10014_MOESM1_ESM.pdf]

nature portfolio

https://doi.org/10.1038/s41586-025-10014-0

Supplementary information

Advancing regulatory variant effect prediction with AlphaGenome

In the format provided by the authors and unedited

Supplementary Information

Advancing regulatory variant effect prediction with AlphaGenome

Žiga Avsec[1*] ✉, Natasha Latysheva[1*], Jun Cheng[1*], Guido Novati[1*], Kyle R. Taylor[1*], Tom Ward[1*], Clare Bycroft[1*], Lauren Nicolaisen[1*], Eirini Arvaniti[1*], Joshua Pan[1*], Raina Thomas[1], Vincent Dutordoir[1], Matteo Perino[1], Soham De[1], Alexander Karollus[1], Adam Gayoso[1], Toby Sargeant[1], Anne Mottram[1], Lai Hong Wong[1], Pavol Drotár[1], Adam Kosiorek[1], Andrew Senior[1], Richard Tanburn[1], Taylor Applebaum[1], Souradeep Basu[1], Demis Hassabis[1] and Pushmeet Kohli[1] ✉

[1]Google DeepMind, [*]These authors contributed equally to this work, ✉avsec@google.com (Z.A.); pushmeet@google.com (P.K.)

Supplementary Methods

Data acquisition and processing

Reference Genomes and Genomic Annotations

AlphaGenome was trained using functional genomics data derived from human and mouse samples. Input sequences were extracted from the hg38 (human) and mm10 (mouse) reference genomes. For sequence intervals that extended beyond chromosomal boundaries, padding with 'N' characters was used to ensure consistent input length. Gene and transcript annotations used for model evaluations were based on the GTF V46 annotations (`https://www.gencodegenes.org/human/release_46.html`), unless otherwise specified.

Genomic coordinate system

For all genome intervals reported in the manuscript, we use a 0-based coordinate system (in which the starting element receives an index of 0, the second receives an index of 1, etc.). Each 0-based interval is non-inclusive or "half-open", meaning that each reported interval includes the basepairs at the *start* position (again, reported in a 0-based manner) up to the base pair at the *end*-1 position, and excludes the base pair at the end position. The width of a 0-based interval is therefore simply *end* - *start.*

For variants, we follow the 1-based coordinate system that is conventional in the human genetics literature.

Metadata Standardization and Data Grouping

To ensure consistent data interpretation and enable robust aggregation across experiments, metadata were standardized using established ontologies. These included UBERON[68] for tissue-level contexts, the Experimental Factor Ontology (EFO[69]) and Cell Line Ontology (CLO[70]) for cell lines, and Cell Ontology (CL[71]) for bulk primary cell type contexts.

Datasets were subsequently grouped to delineate distinct biological contexts for downstream analysis and signal averaging. The primary parameters for this grouping included:

- The assigned ontology term for the biosample, referenced using Compact Uniform Resource Identifiers (CURIEs). CURIEs are standardized, abbreviated codes (e.g., 'UBERON:0001114' for liver) that uniquely identify specific ontology terms.
- The specific assay type (e.g., 'polyA plus RNA-seq' versus 'total RNA-seq').
- The data source (e.g., ENCODE versus GTEx via RECOUNT3).
- Strand information, where applicable.
- For ChIP-seq experiments, the specific transcription factor (TF) or histone modification target (e.g., 'CTCF' or 'H3K27ac') was also a key grouping factor.

Within these groups, genomic signals were averaged across experiments to create representative tracks as detailed in the processing sections for individual assay types below. The selection of which experiments contributed to these averaged tracks was itself guided by hierarchical priority filters (as detailed in relevant sections, such as ENCODE Audit Tags Processing).

Training Data

To generate training targets for AlphaGenome, RNA-seq and epigenomic datasets were sourced from the ENCODE[72], FANTOM5[73], and GTEx[17] consortia. Specifically, this included RNA-seq, PRO-cap, DNase-seq, ATAC-seq, transcription factor (TF) and histone ChIP-seq data from ENCODE; RNA-seq

data from GTEx; CAGE data from Fantom5 portals; and genomic contact maps from the 4D Nucleome portal[74].

ENCODE Audit Tags Processing A consistent quality control (QC) step based on ENCODE's extensive audit system was applied to all ENCODE-derived datasets used in this study. For each experiment, metadata including detailed audit logs were downloaded via the ENCODE REST API (download link: https://www.encodeproject.org/search/?type=Experiment&format=json). Each ENCODE experiment can be associated with multiple audit flags, which are categorized by type and severity level (e.g., ERROR, NOT_COMPLIANT, WARNING; comprehensive details available at https://www.encodeproject.org/data-standards/audits/).

To facilitate an initial quality assessment, we processed these audit results to generate a single binary audit_filter_pass status for each experiment. An experiment was assigned audit_filter_pass = True (i.e., passed this initial QC) unless it contained any 'ERROR' level audits. Furthermore, experiments were also failed (assigned audit_filter_pass = False) if they possessed specific 'NOT_COMPLIANT' flags deemed critical for data quality. These exclusionary 'NOT_COMPLIANT' flags relating to issues with ligation motifs, long-range interactions, insufficient coverage or read counts, poor peak reproducibility or replicate concordance, low TSS enrichment, high PET redundancy, low library complexity, significant bottlenecking, or age consistency concerns (see **Supplementary Table 6** for the comprehensive list and definitions of all exclusionary audit flags). This audit_filter_pass status served as a primary QC filter for all processed ENCODE datasets. The original, more granular audit categories for experiments passing this initial filter were also retained for consideration in subsequent prioritization steps.

RNA-seq Data To capture gene expression information, we integrated RNA-seq data from both ENCODE[72] and GTEx[17].

ENCODE RNA-seq Data Processing of ENCODE RNA-seq data commenced with the metadata file report (downloaded between January 9-17, 2025) and associated experiment, analysis, and biosample reports from the ENCODE portal. We filtered for 'total RNA-seq' and 'polyA plus RNA-seq' assays available as bigWig files for the GRCh38 (hg38) or mm10 assemblies. The selected output types were 'minus strand signal of unique reads', 'plus strand signal of unique reads', and 'signal of unique reads'. Only 'released' files processed in 2020 or later were considered, and files known to have missing chromosome information were excluded.

To focus on baseline biological conditions, biosamples were selected if annotated as non-genetically modified, untreated, and not arrested in a specific cell cycle phase, according to the 'Simple biosample summary' field. Quality control measures included:

1. Applying an audit_filter_pass filter as described in the ENCODE Audit Tags Processing section above.
2. Ensuring a minimum mapped read length of 50 nucleotides (nt).
3. Selecting the single most representative bigWig file per experiment and output type, based on ENCODE's replicate information and processing date.

Finally, to ensure consistency when multiple experiments represented the same biological context, we applied a hierarchical priority filtering step. This favored experiments that, in order:

1. Used non-genetically modified samples (this preference primarily impacts TF ChIP-seq selection).
2. Had less severe audit flags (PASS > WARNING > NOT_COMPLIANT).
3. Used paired-end sequencing.

4. Derived from primary cells (vs. *in vitro* differentiated cells) or tissues (vs. organoids).
5. Originated from more commonly profiled life stages (e.g., adult > embryonic > child).
6. Were not from subcellular fractions.

By default, ENCODE RNA-seq BigWigs represent read coverage per position and come normalized as Reads per Million (RPM). As each read is counted for each alignment position, this means that each bigWig (or bigWig pair for stranded assays) sums to 1 million reads times the read length. To ensure robust aggregation of tracks within a metadata group as defined above, we renormalized bigWigs to a common factor of 1 million reads times a common read length (100 in this work). This re-normalized signal representation was then used for all subsequent analyses.

GTEx RNA-seq Data We utilized processed bigWig files derived from the RECOUNT3 project[75], which are unnormalized. From this, we selected only high-quality samples that corresponded to the set used for eQTL analysis within the GTEx consortium. In contrast to Borzoi, in which specific representative GTEx individuals were selected[2], our analysis incorporated data from all individuals for each tissue. Each individual bigWig file was normalized as RPM similarly to ENCODE.

RNA-seq Processing and Aggregation Normalized RNA-seq tracks from ENCODE and GTEx were grouped by their ontology CURIE and assay type, distinguishing between ENCODE total RNA-seq, ENCODE polyA plus RNA-seq, and GTEx RNA-seq. Within each group, the normalized signals were averaged across all included experiments or individuals. This procedure yielded a final set of consolidated RNA-seq tracks, each representing the average normalized expression profile for a distinct biological context.

CAGE Data Cap Analysis Gene Expression (CAGE) data from the FANTOM5 consortium was used to profile transcription start site (TSS) activity[73,76]. We obtained CAGE Tag Starting Site (CTSS) information as BED files, filtering to include only samples derived from untreated biological conditions. The stranded counts from these CTSS BED files were converted into corresponding stranded signal bigWig files. Following the pre-aggregation normalization strategy applied to RNA-seq data, each individual CAGE signal bigWig track was normalized to represent a total of 100 million reads. These normalized tracks were then grouped based on their mapped ontology CURIE and assay type ('LQhCAGE' or 'hCAGE'). Finally, within each group, the signals from the normalized tracks were averaged to produce a consolidated set of CAGE tracks representing average TSS activity across replicates or related samples for specific biological contexts.

PRO-cap Data For Precision Run-On sequencing with cap analysis (PRO-cap), we utilized a set of 12 stranded, processed bigWig files previously curated for the ProCapNet training dataset[9], obtained directly from the ENCODE portal (`https://www.encodeproject.org`). Consistent with the normalization strategy used for RNA-seq and CAGE data, each of these 12 individual bigWig tracks was normalized so that its signal summed to 100 million. Further aggregation was not needed as the tracks were already unique by biosample and strand.

DNase and ATAC Data DNase-seq and ATAC-seq datasets were obtained from the ENCODE portal. Strict Fraction of Reads in Peaks (FRiP) score thresholds were applied (>10% for both assays, determined via manual inspection to balance coverage versus quality), along with different minimum read length requirements ($\geq$36 nt for DNase-seq, $\geq$45 nt for ATAC-seq, following ENCODE guidelines).

Instead of using ENCODE pre-processed p-value or fold-change signal bigWigs, which can obscure base-resolution information, we ingested the raw alignment (BAM) files for individual replicates. These

BAM files were converted to base-resolution count bigWig files using the reads_to_bigwig.py script (from the ChromBPNet repository[29], https://github.com/kundajelab/chrombpnet/blob/master/chrombpnet/helpers/preprocessing/reads_to_bigwig.py), with default parameters. These files record counts of Tn5 transposase insertions (for ATAC-seq) or DNase-I cleavage sites (for DNase-seq) at each base. This approach preserves the base-resolution nature of the data and facilitates decoupling enzyme cut bias from true signal by applying appropriate read shifts. Specifically, shifts of +4/-4 for ATAC-seq and 0/+1 for DNase-seq were applied to correct for the misalignment of strand-specific Tn5 and DNase-I position weight matrices (PWMs), respectively.

The assays were grouped by biosample ontology CURIE. The resulting base-resolution count BigWigs within each ontology group were then averaged. Following this averaging step, these consolidated tracks underwent post-aggregation normalization, rescaling the signal such that the total counts summed to 100 million insertions per track.

Transcription Factor and Histone ChIP-seq Data Processing of ENCODE transcription factor (TF) and histone ChIP-seq data began with the same comprehensive metadata acquisition from the ENCODE portal (https://www.encodeproject.org/, downloaded January 9-17, 2025) and initial bigWig file filtering criteria (GRCh38/mm10 assemblies, 'released' status, processed 2020 or later, exclusion of files with missing chromosome data) as detailed for ENCODE RNA-seq data. Specific to ChIP-seq, only files representing 'fold change over control' signal were selected.

Key biosample filters, focusing on baseline cellular states (untreated, non-arrested), were applied as described previously, with specific criteria for genetic modifications: Histone ChIP-seq samples were required to be non-genetically modified, while for TF ChIP-seq, genetically modified samples were initially included if necessary (to enable profiling of TFs not typically expressed in the most studied cancer cell lines used by ENCODE), but non-modified samples were strongly preferred during priority filtering.

For quality control:

1. Experiments were filtered based on the audit_filter_pass status, determined as described in the ENCODE Audit Tags Processing section.
2. A frip_filter_pass flag was assigned based on the Fraction of Reads in Peaks (FRiP) score (>1% for TF ChIP-seq, >6% for Histone ChIP-seq). Experiments were retained if they passed either the initial audit or the FRiP filter (audit_filter_pass = True or frip_filter_pass = True), allowing inclusion of high-signal experiments potentially affected by single-replicate audit issues.

A minimum mapped read length of 50 nt was required, consistent with RNA-seq data. Where multiple signal files existed per experiment accession, the most recent file representing the most complete set of biological replicates (guided by the 'Biological replicates' metadata field) was chosen.

Finally, to ensure consistency for experiments representing the same biological context (cell type and target), we applied the hierarchical priority filtering step as outlined in the *ENCODE RNA-seq Data* section. The preference for non-genetically modified samples within this scheme primarily impacts TF ChIP-seq selection.

Following these steps, selected ChIP-seq experiments were grouped by their ontology CURIE, strand, and specific ChIP-seq target (TF or histone modification, **Supplementary Table 2**). Within each group, the 'fold change over control' signal was summed. This signal was then binned to a 128-bp resolution since the signal ENCODE-provided ChIP-seq bigWig files were already smoothed and and provided at coarser 100bp resolution. These resulting ChIP-seq tracks were used without further normalization, as fold-change values inherently account for control signals. We note that while higher resolution ChIP-based assays such as ChIP-exo and ChIP-nexus yield reliable base-pair resolution signal, these tracks are rare and the vast majority of ENCODE chromatin immunoprecipitation was done using ChIP-seq, which is not

inherently base-resolution. Given the substantial data processing effort that would be required to curate non-ENCODE base pair resolution ChIP data, we deferred base-resolution modeling for TF binding to future work.

Summary of data processing parameters **Supplementary Table 7** provides a consolidated summary of the data processing parameters applied to generate each type of 1D genomic track used in this study. It outlines critical aspects for each assay, including data sources, quality control filters, normalization approaches, and final aggregation methods.

Preparation of RNA-seq Data for Splicing Analyses Training data for AlphaGenome's splicing-related predictions (splice junctions, splice site usage (SSU), and splice site classification) were derived from the same ENCODE and GTEx RNA-seq datasets used for gene expression analyses, and samples were grouped by ontology CURIEs consistently with the RNA-seq processing pipeline.

To quantify splice junction count, reads from each RNA-seq sample were realigned using STAR (version 2.7.11b)[77] from the BAM files downloaded from GTEx and ENCODE. For human samples, alignment was performed against the GRCh38.p13 reference genome, with GENCODE v32 gene annotations guiding splice junction discovery. For mouse samples, the GRCm38.p6 reference genome and GENCODE vM23 annotations were used.

Key STAR alignment parameters were configured to optimize for junction detection, including setting a minimum splice junction overhang of 8 base pairs (`--alignSJoverhangMin 8`), allowing a maximum of 20 alignments for multi-mapping reads (`--outFilterMultimapNmax 20`), defining standard intron size limits (e.g., `--alignIntronMin 20`, `--alignIntronMax 1000000`), and outputting strand information derived splice dinucleotide motifs (`--outSAMstrandField intronMotif`).

The precise STAR command used was:

```
STAR --runMode alignReads \
 --genomeDir "gcs/${GENOME}" \
 --readFilesType ${read_file_type} \
 --readFilesCommand ${read_file_command} \
 --readFilesIn ${input_path} \
 --outSAMtype BAM Unsorted \
 --outFilterMultimapNmax 20 \
 --alignSJoverhangMin 8 \
 --alignSJDBoverhangMin 1 \
 --outFilterMismatchNmax 999 \
 --outFilterMismatchNoverReadLmax 0.04 \
 --alignIntronMin 20 \
 --alignIntronMax 1000000 \
 --alignMatesGapMax 1000000 \
 --outSAMstrandField intronMotif --outSAMunmapped Within \
 --outFileNamePrefix "${output_prefix}" \
 --outTmpDir data/tmp
```

The primary output files containing splice junction information (`sj.out.tab`) from STAR served as the raw data for all subsequent splicing data curation, specifically to define training targets for the three types of splicing-related predictions. Samtools (version 1.21)[78] was used for standard intermediate processing of BAM files generated during alignment.

1. Splice Junction Quantification, Filtering, and Normalization Individual splice junction output files for each sample were first combined into a single comprehensive table, indexed by chromosome, junction start coordinate, junction end coordinate, and strand, separately for human and mouse species.

A stringent quality filtering pipeline was then applied to these compiled junction lists using the `splicemap` package (available at https://github.com/gagneurlab/splicemap) to ensure high data fidelity:

- *Human GTEx Samples*: A junction was retained if its 90th percentile read count across all GTEx samples was greater than 1, AND the median of total read counts supporting any alternative splicing event sharing either its donor or acceptor site was at least 1.
- *Human ENCODE Samples*: Junctions from human ENCODE RNA-seq samples were filtered against the set of high-confidence junctions derived from GTEx; any ENCODE human RNA-seq splice junction not present in the filtered GTEx junction set was discarded.
- *Mouse ENCODE Samples*: For mouse ENCODE RNA-seq samples, a junction was retained if its median read count across all mouse RNA-seq samples within the same ontology CURIE group was greater than 3.

After these filtering steps, the retained junction counts for each sample were normalized to 1 million total filtered junction reads per sample.

For use in model training (loss calculation) and evaluation, these normalized junction counts underwent further tissue-specific preprocessing. Within each tissue:

1. Raw counts were first clipped at the 99.99th percentile for that tissue to mitigate the influence of extreme outliers.
2. Subsequently, these clipped counts were scaled by dividing by the mean count value, where this mean was calculated only across actively expressed junctions (defined as those with a clipped count > 0) within that specific tissue.

During training on splice junctions, the donor-acceptor pairing of a maximum of 512 splice sites on each strand are considered per input interval. If the sampled interval has more than 512 splice sites, we narrow the interval size to consider splice junctions until a maximum of 512 splice sites are in the interval on either strands. (Note: This only affects the interval used for inference; the input sequence length of the model, for example 1 Mb, remains unchanged). For each strand and each CURIE condition, splice junction counts are represented as a square matrix of shape [512, 512] corresponding to donor/acceptor pairs. Each element of the matrix represents the observed normalized read counts for the donor/acceptor pair. If less than 512 splice sites are observed, the matrix is padded to 512 with 0.

2. Splice Site Usage Splice Site Usage (SSU) was calculated for each potential splice site using the formula:

$$\text{SSU} = \frac{\text{\# reads using the splice site}}{\text{\# reads using the splice site} + \text{\# reads supporting skipping of the splice site}}$$

We adapted the basic splice site strength definition from Dent et al[79]. SSU quantification was performed using a custom script. For this calculation, we considered all reads spanning the splice sites regardless of the strand. SSU counting was done with a custom script. Reads flagged as PCR/optical duplicates, those with a mapping quality (MQ) below 30, or reads containing base calls with a base quality (BQ) below 20 were excluded from the counts. The counting of each RNA-seq sample was performed independently, and the SSU for each RNA-seq sample was calculated independently. Only splice sites

that were detected in the corresponding STAR splice junction output (`sj.out.tab` files) and passed the above splice junction filtering step were considered in this quantification process.

The splice site usage has two tracks per CURIE condition, corresponding to the two strands. Unlike splice sites, SSU does not distinguish between donor usage and acceptor usage.

3. Splice Site Definition for Classification The set of splice sites used for training the splice site classification task was defined as the union of all unique donor and acceptor sites present in the filtered splice junction data (from step 2 above) for each ontology CURIE. Notably, unlike splice junction counts or SSU values which can be tissue/sample-specific, the defined splice site training examples were not treated as tissue-specific.

The splice site classification task was formulated as a 5-class classification problem, where each relevant position could be classified as:

- Donor site on the positive strand (Donor+)
- Acceptor site on the positive strand (Acceptor+)
- Donor site on the negative strand (Donor-)
- Acceptor site on the negative strand (Acceptor-)
- Not a splice site on either strand

Contact maps Chromatin contact maps, which represent average inter-nucleotide contact probabilities typically derived from assays like Hi-C or Micro-C, are crucial for understanding gene regulation via 3D genome organization. We sourced contact map datasets from the 4D Nucleome portal (https://data.4dnucleome.org/, accessed 2021/03/04), aiming for quality comparable to or exceeding that of the 5 datasets used by Akita[80] and the 2 datasets used by Orca. To achieve this, we selected datasets provided as multi-resolution cooler files (.mcool) that were at least 7.74 GB (the size of the smallest Akita contact map). We retained only those files that were at 1000 bp resolution. This resulted in a curated collection of twenty-eight human and eight mouse datasets (see **Supplementary Table 2** for file accessions), which importantly includes the 5 Akita and 2 Orca datasets. We did not directly compare our model's performance to Akita's, as their dataset preprocessing includes an additional Gaussian blurring step not present in Orca's methods or ours, which prevents direct output comparisons.

We preprocessed these contact maps following the protocol established for the Orca 1 Mb model[14]. Briefly, 1000 bp resolution contact maps underwent two standard preprocessing steps: matrix balancing to scale the values and adaptive coarse-graining to apply smoothing. Subsequently, a distance-based normalization was applied:

- First, an average coarse-grained contact value was computed for each pairwise genomic distance ($mean[abs(i-j)]$) across each dataset (excluding all zero-valued bins).
- Distance-based normalization was then applied: the normalized contact map values y(i, j) were computed as the log-fold change over the distance-dependent means: $y[i,j] = \log((x[i,j] + eps)/(mean[abs(i-j)] + eps))$. Here $x[i,j]$ is the coarse-grained count and the numerical relaxation constant, eps, was set to the minimum value of the mean profile.

During model training, these preprocessed 1000 bp resolution contact matrices were interpolated to the 2048 bp resolution of AlphaGenome's pairwise representation blocks and aligned with the sampled input intervals. This interpolation used an area-weighted averaging scheme, where the value for each target 2048 bp bin was the average of overlapping source 1000 bp bins, weighted by their fractional area of overlap.

Dataset splitting and cross-validation For a robust comparison with Borzoi and other baselines, we utilized the identical cross-validation fold definitions (*fold-0*, *fold-1*, *fold-2*, and *fold-3*) previously established by Borzoi for both human and mouse genomes (sequences_*.bed.gz files in `https://github.com/calico/borzoi/tree/5c9358222b5026abb733ed5fb84f3f6c77239b37/data`). Each genome is split into eight distinct sections; each fold uses six sections for training, one for validation, and one for testing. In addition to these cross-validation setups, we also trained comprehensive *all-folds* models using all eight genomic sections for the training set; these models were exclusively evaluated on variant interpretation benchmarks.

Within the genomic regions assigned to each set by these folds, we specifically used the pre-defined target intervals (approximately 196kb) defined in the Borzoi study. Since AlphaGenome operates on 1 Mb input and output sequences, each 1 Mb input window was centered around the midpoint of the corresponding 196kb Borzoi target interval. Crucially, to prevent data leakage between sets arising from AlphaGenome's larger input window, a strict exclusion criterion was applied: any validation or test interval was removed from its respective set if its corresponding 1 Mb AlphaGenome input window overlapped with any 1 Mb input window derived from a training interval within that same fold.

Final data representations Processed genomic tracks, serving as model prediction targets, were converted to brain floating point (`bfloat16`) format for numerical efficiency and stored in z-standard compressed sharded matrices. Most data tracks were maintained at base-pair resolution, except for ChIP-seq (TF and Histone) tracks, which represent fold-change values, and were stored as base-resolution cumulative sums. This strategy allows for efficient querying of their average signal at 128 bp resolution.

The model directly uses the track values resulting from the upstream processing steps, which typically represent normalized read or insertion counts (often scaled to a total of 100 million signals per track for sequencing assays) or fold-change enrichments (for ChIP-seq). No additional scaling transformations (e.g., log-scaling or z-score normalization across tracks) are applied before model input. Predicted splice junction counts are an exception; while their values are additionally scaled (as described in the training methods), this does not affect their primary use in calculating ratios for relative splice site assessment.

Model

AlphaGenome is a deep learning based model that processes a 1 Mb DNA sequence along with a species identifier (human or mouse) to predict a diverse array of genomic features. These outputs include 1D tracks representing signals such as chromatin accessibility, TF binding, and gene expression at various resolutions; 2D chromosomal contact maps; and specific splicing-related predictions like splice site probabilities, splice site usage (SSU), and splice junction counts.

AlphaGenome employs an encoder-decoder framework that integrates convolutional and transformer components, drawing inspiration from models such as Enformer[1] and Borzoi[2]. It introduces several key architectural features, including a U-Net-style decoder incorporating residual (skip) connections from the encoder. The model processes information through five core components: (1) a sequence encoder, (2) a transformer tower, (3) pairwise interaction blocks, (4) a sequence decoder, and (5) task-specific output heads. AlphaGenome has approximately 450 million trainable parameters (20% in the encoder, 28% in the sequence transformer, 15% in the pairwise blocks, 25% in the decoder, and 12% in the output embedding and prediction heads). The model was implemented using JAX[81] and Haiku[82].

Sequence Encoder

The sequence encoder progressively downsamples the input DNA sequence from 1 bp resolution (length 1 Mb or 2^{20} bp) to 128 bp resolution embeddings (length 8192 or 2^{13}) over 7 stages. The number of feature channels increases from 768 up to 1536 to enhance representational capacity. This downsampling

is achieved through repeated convolutional blocks, each followed by max-pooling with a stride of 2. The corresponding pseudocode is as follows:

```
def conv_block(x: Array, num_channels: int, width: int = 5) -> Array:
  x = RMSBatchNorm(x)
  x = GeLU(x)
  if width == 1:
    x = Linear(num_channels=num_channels)(x)
  else:
    x = StandardizedConv1D(num_channels=num_channels, width=width)(x)
  return x

def dna_embedder(x: Array):
  out = Conv1D(num_channels=768, width=15)(x)
  return out + conv_block(out, num_channels=768)

def downres_block(x: Array) -> Array:
  # Increase number of channels by 128 and apply skip connection by padding.
  out = conv_block(x, num_channels=x.shape[-1] + 128)
  out = out + Pad(x, [(0, 0), (0, 0), (0, 128)])
  return out + conv_block(out, num_channels=out.shape[-1])

def sequence_encoder(x: Array) -> tuple[Array, map[str, Array]]:
  intermediates = {}
  for bin_size in [1, 2, 4, 8, 16, 32, 64]:
    block = dna_embedder if bin_size == 1 else downres_block
    x = block(x)
    intermediates[f'bin_size_{bin_size}'] = x
    x = MaxPool(pool_size=2)(x)
  return x, intermediates
```

The core component of the above code is a `conv_block`, which applies root mean square batch normalization (`RMSBatchNorm`—same as `BatchNorm`[83] with learned scale and offset, but without shifting by the sample mean), a GeLU[84] activation, and then a standardized 1D convolution (`StandardizedConv1D`) with a kernel width of 5. Following Brock *et al.*[85], these convolutions employ scaled weight standardization – re-parameterizing by standardizing and scaling the weights – which we found effective for stabilizing activation magnitudes. Each `RMSBatchNorm` layer maintains an exponential moving average (EMA, decay 0.9) of per-channel variance during training; this EMA variance is used for normalization during inference. To facilitate training and gradient flow[86], residual skip connections are added around each convolutional block. When channel dimensions differ across a residual connection (as in `downres_block`), the input is padded with zeros before addition. All convolution layers used "same" padding.

The initial stage uses the `dna_embedder` block, which applies a wider 1D convolution (kernel width 15, embedding to 768 channels), similar to Enformer[1], followed by a residual `conv_block`. The subsequent six `downres_block` stages each apply two `conv_block` layers (the first increasing the channel count by 128) with skip connections. Intermediate representations from each of the 7 stages, corresponding to resolutions of 1 bp, 2 bp, 4 bp, . . . , 64 bp before the max-pooling step, are stored for use as U-net skip connections in the sequence decoder. The final output of the encoder has a resolution of 128 bp, sequence axis length 8192 (1 Mb / 128 bp) and 1536 channels (768 + 6 * 128).

Transformer Tower

Following the encoder, a transformer tower processes the 128 bp resolution sequence embeddings to model long-range interactions across the full 1 Mb input context. The tower consists of 9 stacked transformer blocks. Each block contains a multi-head attention (MHA) layer followed by a two-layer perceptron (MLP) layer, both wrapped in residual connections. We use standard array dimension conventions where 'B' denotes batch size, 'S' sequence length ($S = 1Mb/128\ bp = 8192$), 'C' channels, 'P' pairwise sequence length, and 'F' pairwise channels. In pseudocode, these components can be written as:

```
def mha_block(x: Array[B, S, C], attention_bias: Array[B, 8, S, S]) -> Array[B, S, C]:
  x = RMSBatchNorm(x)
  # Multi-query attention: 8 query heads, 1 shared key/value head. Each
  # query and key head has 128 channels. The value head has 192 channels.
  q = LayerNorm(Linear((8, 128), with_bias=False)(x), axis=-1)
  k = LayerNorm(Linear((1, 128), with_bias=False)(x), axis=-1)
  v = LayerNorm(Linear((1, 192), with_bias=False)(x), axis=-1)
  # Apply RoPE with max_position = S = 8192 (1 Mb / 128 bp).
  q = apply_rope(q, max_position=8192)
  k = apply_rope(k, max_position=8192)
  attention_logits = Einsum('bshc,bS1c->bhsS', q, k) / sqrt(128)
  # Add bias from pairwise activations and soft-clip logits in [-5, 5].
  attention_logits = Tanh((attention_logits + attention_bias) / 5.0) * 5.0
  attention_weights = Softmax(attention_logits, axis=-1)
  y = Einsum('bhsS,bS1c->bshc', attention_weights, v)
  # Reshape and project back to input channels C = 1536.
  y = Linear(x.shape[-1])(y.reshape(y.shape[:2] + (-1,)))
  return Dropout(RMSBatchNorm(y))

def mlp_block(x: Array[B, S, C]) -> Array[B, S, C]:
  y = RMSBatchNorm(x)
  y = Linear(2 * x.shape[-1])(y)
  y = Dropout(ReLU(x))
  y = Linear(x.shape[-1])(y)
  return Dropout(RMSBatchNorm(y))

def attention_bias_block(x: Array[B, P, P, F]) -> Array[B, 8, S, S]:
  x = GeLU(RMSBatchNorm(x))
  x = Linear(8, with_bias=False)(x)
  # Repeat attention bias to S = 8192 length (factor S/P = 8192/512 = 16).
  return Moveaxis(Repeat(x, 16, axis=(1, 2)), 3, 1)

def transformer_tower(x: Array[B, S, C]) -> tuple[Array[B, S, C], Array[B, P, P, F]]:
  pair_x = None
  for i in range(9):
    if i % 2 == 0:
      pair_x = pair_update_block(x, pair_x)
    x = x + mha_block(x, attention_bias=attention_bias_block(pair_x))
    x = x + mlp_block(x)
  return x, pair_x
```

Before every second MHA block, pairwise representations at 2048 bp resolution ($P = 1Mb/2048\ bp = 512$) are initialized or updated based on the sequence embeddings (detailed in Pairwise blocks

section). They are primarily used for contact map prediction but also provide a bias term to the MHA layers. The `attention_bias_block` function projects the pairwise channels (F) to match the number of attention heads (8) and then repeats the result 16 times (2048 bp / 128 bp) along the two sequence axes to match the sequence length dimension. This bias is added directly to the attention logits before the softmax, analogous to techniques used in AlphaFold2[87].

Our MHA implementation (`mha_block`) uses multi-query attention[88], where key (k) and value (v) projections are shared across all 8 attention heads, reducing computational cost. We apply smooth clipping to the attention logits[89], constraining them to the range [-5.0, 5.0] using a scaled hyperbolic tangent function before the `Softmax`. Relative positional information is incorporated using Rotary Position Embeddings (RoPE[90]) applied to query and key vectors. We use a modified calculation for the inverse frequencies compared to the standard RoPE formulation (`apply_rope`). This modification reduces the density of rotational frequencies corresponding to short relative distances and is applied consistently throughout the model. The core of this implementation is detailed in pseudocode:

```
def apply_rope(x: Array, max_position: int, positions: Array | None = None):
  positions = positions or Arange(x.shape[1])
  num_freq = x.shape[-1] // 2
  freq = 1.0 / (Arange(num_freq) + Geomspace(1, max_position - num_freq + 1, num_freq))
  theta = Repeat(Einsum('...s,f->...sf', positions, freq), 2, axis=-1)
  x_rotated = Stack((-x[..., 1::2], x[..., ::2]), axis=-1).reshape(x.shape)
  return x * Cos(theta) + x_rotated * Sin(theta)
```

The MLP block (`mlp_block`) follows standard transformer design, using ReLU activation and expanding the number of channels by a factor of 2 in the hidden layer. Dropout is applied within the MHA and MLP blocks during training only, with rates of 0.3 for pre-training and 0.1 for distillation.

Pairwise Blocks

Interleaved with the transformer tower blocks (specifically, executed before the MHA layer in blocks 0, 2, 4, 6, and 8), `pair_update_block` modules operate on and update a 2D tensor representing pairwise interactions between sequence positions. These pairwise representations have 2048 bp resolution (sequence length $P = 512$) and have $K = 128$ channels; they are primarily used for contact map prediction but also contribute a bias to the transformer's attention mechanism (described in the Transformer Tower section). Each `pair_update_block` first computes an update from the sequence embeddings (`sequence_to_pair_block`), which initializes or is added to the previous pairwise state, and then performs two residual refinement steps (`row_attention_block` and `pair_mlp_block`).

```
def pair_update_block(
 sequence_input: Array[B, S, C], pair_input: Array[B, P, P, F] | None
) -> Array[B, P, P, F]:
  y = sequence_to_pair_block(sequence_input)
  x = y if pair_input is None else pair_input + y
  x += row_attn_block(x)
  x += pair_mlp_block(x)
  return x
```

The `sequence_to_pair_block` function first downsamples the 128 bp resolution sequence embeddings ($S = 8192$) to 2048 bp resolution ($P = 512$) using average pooling. It then computes pairwise features using a form of attention enhanced with relative positional encodings. The positional encodings are generated by `central_mask_features` using 32 exponentially spaced distance thresholds. Directionality is then incorporated by concatenating these 32 features with a copy multiplied by the sign

of the relative distance, yielding a 64-channel positional representation. This relative attention term is calculated efficiently using the `relative_shift` operation, as detailed in Transformer-XL[91]. The final sequence-to-pair representation is obtained by projecting the combined query-key and relative position terms, adding an outer sum of projections derived from the sequence embeddings, and applying dropout.

```
def sequence_to_pair_block(x: Array[B, S, C]) -> Array[B, P, P, F]:
  # Downsample sequence to P=512 length (Factor S/P = 8192/512 = 16).
  x = RMSNorm(AvgPool(pool_size=16)(x))
  # 32 query and key heads with 128 feature channels each.
  q = Linear((32, 128), with_bias=False)(x)
  k = Linear((32, 128), with_bias=False)(x)
  # Generate and project the directional positional relative encodings.
  pos_features = central_mask_features(sequence_length=512, feature_size=64)
  pos_encoding = Linear((32, 128))(pos_features)
  q_bias = GetParameter('q_r_bias', (1, 1, 32, 128))
  k_bias = GetParameter('k_r_bias', (1, 1, 32, 128))
  rel_q_a = relative_shift(Einsum('bqhc,bphc->bqph', q + q_bias, pos_encoding))
  rel_k_a = relative_shift(Einsum('bkhc,bphc->bkph', k + k_bias, pos_encoding))
  a = Einsum('bqhc,bkhc->bqkh', q, k) + (rel_q_a + rel_k_a.swapaxes(1, 2))/2
  # Additional projection based on outer sum of sequence embeddings.
  y_q = Linear(128, with_bias=False)(GeLU(x))
  y_k = Linear(128, with_bias=False)(GeLU(x))
  pair_activations = Linear(128)(a) + y_q[:, :, None, :] + y_k[:, None, :, :]
  return Dropout(pair_activations)

def central_mask_features(sequence_length: int, feature_size: int):
  # `relative_positions` spans from the min to the max `i_k - i_q`, i.e. -(L-1) to +(L-1)
  relative_positions = Arange(2 * sequence_length - 1) - (sequence_length - 1)
  center_widths = Arange(feature_size // 2) + Geomspace(
      1, sequence_length - feature_size // 2 + 1, feature_size // 2, endpoint=False)
  embeddings = (center_widths[None, :] > Abs(relative_positions)[:, None])
  return Concatenate(
      [embeddings, Sign(relative_positions)[:, None] * embeddings], axis=-1)

def relative_shift(x: Array[..., S, 2 * S - 1]) -> Array[..., S, S]:
  *batch_shapes, seq_length, num_diagonals = x.shape
  x = Pad(x, [(0, 0)] * (len(batch_shapes) + 1) + [(1, 0)])
  x = x.reshape(batch_shapes + [num_diagonals + 1, seq_length])
  x = x[..., 1:, :].reshape(batch_shapes + [seq_length, num_diagonals])
  return x[..., :seq_length]
```

The pairwise embeddings undergo further refinement by two transformer-style operations, each within a residual connection. The `row_attention_block` implements a self-attention mechanism that operates only along the second axis of the `PxP` pairwise matrix (each position (`i,j`) attends to all positions (`i,k`) in the same row `i`). We found that applying a second attention block that operates over columns yielded no empirical accuracy gains for downstream tasks (likely due to the explicit symmetrization applied before contact map prediction) and was less computationally efficient, particularly in distributed training settings. The `pair_mlp_block` then applies a standard two-layer MLP with a ReLU activation and 2x channel expansion for further feature refinement.

```
def row_attention_block(pair_input: Array[B, P, P, F]) -> Array[B, P, P, F]:
  x = RMSNorm(pair_input)
```

```
  # Single queries, keys and values heads with 128 feature channels.
  k = Linear(128, with_bias=False)(x)
  q = Linear(128, with_bias=False)(x)
  v = Linear(128)(x)
  x = Einsum('bpPf,bpkf->bpPk', q, k) / Sqrt(128)
  x = Einsum('bpPk,bpkf->bpPf', Softmax(x, axis=3), v)
  return Dropout(x)

def pair_mlp_block(pair_input: Array[B, P, P, F]) -> Array[B, P, P, F]:
  x = RMSNorm(pair_input)
  x = Linear(2 * pair_input.shape[-1])(x)
  x = Linear(pair_input.shape[-1])(ReLU(x))
  return Dropout(x)
```

Sequence Decoder

The sequence decoder mirrors the encoder's structure but operates in reverse: it progressively upsamples the 128 bp resolution embeddings (output from the transformer tower, with 1536 channels) back to the original 1 bp resolution, while simultaneously decreasing the number of feature channels down to 768. This is achieved through 7 iterative stages, each applying an `upres_block` module. As is standard in U-Net architectures[13], the decoder incorporates skip connections, adding embeddings (`intermediates`) stored at corresponding resolutions during the encoder pass. The corresponding pseudocode is as follows:

```
def upres_block(x: Array, unet_skip: Array) -> Array:
  num_channels = unet_skip.shape[2]
  out = conv_block(x, num_channels) + x[:, :, :num_channels]
  out = Repeat(out, 2, axis=1) * GetParameter('residual_scale', (1,), init=0.9)
  out += conv_block(unet_skip, num_channels, width=1)
  return out + conv_block(out, num_channels)

def sequence_decoder(x: Array, intermediates: map[str, Array]) -> Array:
  for bin_size in [64, 32, 16, 8, 4, 2, 1]:
    x = upres_block(x, intermediates[f'bin_size_{bin_size}'])
  return x
```

Each `upres_block` takes the output from the previous stage and the corresponding resolution skip connection from the encoder. First, a `conv_block` reduces the channel dimension of the embedding to match that of the skip connection. As usual, the convolution is paired with a residual connection which handles the reduction in channel dimension by cropping. The result is then upsampled by a factor of two along the sequence length axis via repetition and scaled by a learnable coefficient specific to that resolution level. Concurrently, the incoming skip connection is processed through its own `conv_block` (using a kernel width of 1). This processed skip connection is then added to the scaled, upsampled representation. Finally, this combined tensor passes through one more `conv_block`, again including an internal residual connection, before being passed to the next stage or returned as the final 1 bp resolution sequence embedding.

Output Heads

The AlphaGenome architecture, after processing the input DNA through its encoder, transformer, and decoder stages, produces key internal representations at multiple resolutions. These learned embeddings (specifically the 1D embeddings at 1 bp and 128 bp resolution along the sequence, and the 2D

pairwise embedding at 2048 bp resolution) form the basis for predicting a diverse array of genomic features. Predictions are adapted to the specific organism (human or mouse) by incorporating learned, organism-specific embeddings within these functions. The following pseudocode illustrates the core data flow that generates these embeddings via the main model components (`sequence_encoder`, `transformer_tower`, `sequence_decoder`) and output embedding functions (`output_embedder`, `output_pair`).

```
def output_embedder(x: Array, organism_index: int, skip_x: Array | None = None) -> Array:
  x = Linear(2 * x.shape[2])(x)
  if skip_x is not None:
    skip_x = Linear(x.shape[2], with_bias=False)(skip_x)
    x += Repeat(skip_x, x.shape[1] // skip_x.shape[1], axis=1)
  return GeLU(RMSBatchNorm(x) + Embedding(x.shape[2])(organism_index))

def output_pair(x: Array, organism_index: int) -> Array:
  x = (x + Swapaxes(x, 1, 2)) / 2.0 # Symmetrize.
  return GeLU(RMSNorm(x) + Embedding(128)(organism_index))

def model_embeddings(input_dna: Array, organism_index: int) -> tuple[Array, Array, Array]:
  trunk, intermediates = sequence_encoder(input_dna)
  trunk += Embedding(1536)(organism_index)
  trunk, pair_activations = transformer_tower(trunk)
  x = sequence_decoder(trunk, intermediates)
  embeddings_128bp = output_embedder(trunk, organism_index)
  embeddings_1bp = output_embedder(x, organism_index, embeddings_128bp)
  embeddings_pair = output_pair(pair_activations, organism_index)
  return embeddings_1bp, embeddings_128bp, embeddings_pair
```

Beyond the organism-specific embeddings applied during the generation of these shared representations (`embeddings_1bp`, `embeddings_128bp`, `embeddings_pair`), each output head itself learns a distinct set of parameters for human and mouse predictions. The subsequent sections detail the specific parameterization and loss function defined for each prediction modality. The total training loss for AlphaGenome is the sum of these losses defined by each head with no additional weighting coefficients.

RNA-seq, CAGE, ATAC, DNase and PRO-Cap Output Heads Predictions for these assays are generated from both the 1 bp and 128 bp resolution embeddings. Each resolution uses a dedicated head consisting of a linear layer mapping the input channel dimension to the number of target tracks for that assay type. A softplus activation is applied to the linear layer's output, which is then multiplied by a learnable, per-track positive scaling factor, ensuring non-negative outputs. Strand-specific readouts are treated as distinct output tracks. Each head predicts tracks as in the pseudocode:

```
def tracks_scaled_predictions(x: Array[S, C], num_tracks: int) -> Array[S, num_tracks]:
  x = Linear(num_tracks)(x)
  scale = GetParameter('scale', (num_tracks,), init=0.0)
  return Softplus(x) * Softplus(scale)
```

To improve numerical stability, experimental target tracks undergo a scaling procedure before loss calculation (`targets_scaling`). Tracks are first normalized by dividing by the mean of their non-zero values (pre-calculated per track across the entire genome during dataset generation). High values are then dampened using square-root-based smooth clipping. For RNA-Seq tracks only, an additional power

transformation is applied for further compression. These scaling and clipping operations are reversed (`predictions_scaling`) when evaluating model predictions against the original experimental data. The scaling and inverse scaling functions are:

```
def targets_scaling(
  targets: Array[S, C], track_means: Array[C], apply_squashing: bool
) -> Array[S, C]:
  targets = targets / track_means
  if apply_squashing: # Applied RNA-seq tracks only.
    targets = targets ** 0.75
  return Where(targets > 10.0, 2 * Sqrt(x * 10.0) - 10.0, targets)

def predictions_scaling(
  x: Array[S, C], track_means: Array[C], apply_squashing: bool
) -> Array[S, C]:
  x = Where(x > 10.0, (x + 10.0) ** 2 / (4 * 10.0), x)
  if apply_squashing:
    x = x ** (1.0 / 0.75)
  return x * track_means
```

The training loss follows the approach of Borzoi, calculated as a weighted sum of Poisson and Multinomial negative log-likelihoods (NLL). For each track, the relevant sequence axis (e.g., 2^{20} bins for 1 bp, 8192 bins for 128 bp resolution outputs) is divided into 8 equal segments. Within each segment, the loss combines two components:

1. A Multinomial NLL term comparing the predicted distribution of counts across bins to the target distribution, which ensures the model learns the relative values and overall shape of the predictions within each segment. This multinomial component is weighted by a factor of 5.0, as in Borzoi.
2. A Poisson NLL term comparing the sum of predicted and target counts for each segment, which encourages the model to learn the accurate overall count (regardless of shape). This term is scaled inversely by the segment length (`multinomial_resolution` = 2^{17}).

Calculating the loss over 8 segments, rather than the full sequence, mitigates numerical instability (using smaller segments was found to empirically degrade performance). The loss function is implemented as shown in the pseudocode:

```
def multinomial_loss(
  x: Array[S, C], targets: Array[S, C], multinomial_resolution: int
) -> Array[]:
  x = x.reshape((-1, multinomial_resolution, x.shape[-1]))
  targets = targets.reshape((-1, multinomial_resolution, targets.shape[-1]))
  sum_pred = Sum(x, axis=1, keepdims=True)
  sum_target = Sum(targets, axis=1, keepdims=True)
  poisson_loss = Sum(sum_pred - sum_target * Log(sum_pred + 1e-7))
  multinomial_prob = x / (sum_pred + 1e-7)
  positional_loss = Sum(-targets * Log(multinomial_prob + 1e-7))
  return (poisson_loss / multinomial_resolution + 5.0 * positional_loss)
```

For RNA-seq tracks, an additional loss component, inspired by Decima[44], promotes tissue-specific expression patterns. First, predicted and scaled target counts are aggregated within annotated gene

boundaries found in the input interval and subsequently normalized by gene length to yield per-gene counts. These per-gene counts are then used to compute a loss across the tissue/cell type dimension for each gene. This loss calculation mirrors the structure of the main track loss described above: it combines a Poisson NLL term on the total normalized expression per gene across all tissues and a Multinomial NLL term on the distribution across tissues. The Multinomial term within this calculation is weighted by a factor of 5.0. This auxiliary gene-level loss contributes to the model's total training loss with an overall weight of 0.1.

After all model training runs had finished, we identified an implementation error affecting the RNA-seq output head's tissue-specific gene expression loss. This error resulted in incorrectly flipped gene boundary strands for half of the training intervals. We have since verified that, due to the low relative weight and magnitude of this auxiliary loss term, the error's impact on final model performance was within the range of expected noise, as measured by the Pearson correlation of log-fold change in gene expression.

TF ChIP-seq and Histone ChIP-seq Output Heads These tracks are predicted from the 128 bp resolution embeddings. The prediction head structure, target scaling (without squashing), loss calculation (Poisson and Multinomial over 8 segments), and inverse scaling are identical to those used for the other genomic tracks described above, but applied only to the 128 bp resolution pathway (e.g., `multinomial_resolution` = $2^{17}/128 = 1024$).

Contact Maps Output Head Contact frequency predictions are derived from the 2048 bp resolution pairwise embeddings. The raw pairwise activations are first explicitly symmetrized. An organism-specific embedding is added, followed by `RMSNorm` and `GeLU` activation. A final linear transformation (not shown in snippets) maps these processed pairwise embeddings (with $K = 128$) to the predicted target contact map tracks. The contact map outputs are trained using a mean squared error (MSE) loss between the predictions and the targets.

Splice Sites Classification Output Head This head predicts the probability of each base belonging to one of five classes: Donor+, Acceptor+, Donor-, Acceptor-, or not a splice site (see Splice Site Definition for Classification section in Training Data). A linear layer is applied to the 1 bp resolution embeddings that maps to 5 logits per base, followed by a Softmax activation to produce class probabilities. Training uses a standard per-base cross-entropy loss function against the true splice site labels.

Splice Site Usage Output Head This head predicts the proportion of splicing events utilizing each potential splice site (SSU), separately for each strand and tissue/cell type. It takes the 1 bp resolution embeddings as input. A linear layer maps the embedding dimension to the number of tissues/cell types per strand at each potential splice site location, followed by a Sigmoid activation to constrain outputs between 0 and 1. The model is trained using a binary cross-entropy loss against the observed SSU values.

Splice Junctions Output Head This head predicts counts for potential splice junctions between donor and acceptor sites. It operates on the 1 bp resolution embeddings. Because each sequence in the batch might have a different number of donor and acceptor splice sites, in practice we perform the calculation using batch padding. In the pseudocode below, we show the calculation for a single sequence:

```
def tissue_scaled_rope(x: Array[S, 768], indices: Array[P]) -> Array[P, num_tissues, 768]:
  x = x[indices, :]
  scale = GetParameter('scale', (num_tissues, 768))
```

```
  offset = GetParameter('offset', (num_tissues, 768))
  x = scale[None, :, :] * x[:, None, :] + offset[None, :, :]
  return apply_rope(x, max_position=2 ** 20, positions=indices)

def splice_junctions(
  x: Array[S, C], donor_indices: Array[D], acceptor_indices: Array[A]
) -> Array[D, A, N_tissues]:
  x = Linear(768)(x)
  donor_embedding = tissue_scaled_rope(x, donor_indices)
  acceptor_embedding = tissue_scaled_rope(x, acceptor_indices)
  return Softplus(
      Einsum('dtk,atk->dat', donor_embedding, acceptor_embedding))
```

First, a linear layer projects the embeddings to an intermediate dimension. For sequence-level pre-training and evaluation (as described in this section), the tissue-specific genomic coordinates of expressed donor (`donor_indices`) and acceptor (`acceptor_indices`) sites, derived from the data processing pipeline, are provided as input. During distillation, these sites are obtained by selecting the top-k (with $k = 512$) highest probability sites from the teacher's splice site classification head. The procedure for identifying splice sites differs during variant effect prediction, as described in the Variant Scoring section. The projected embeddings corresponding to these ground truth site coordinates are then processed by the `tissue_scaled_rope` function. This function first extracts the embeddings at the provided site locations and then applies the modified RoPE implementation described previously. Because splice sites are not contiguous, the function uses relative genomic coordinates as positions to incorporate distance information accurately. The modification to RoPE's frequency calculation is particularly relevant here, reducing density at short ranges to better focus on the longer distances typical for splice junctions. Finally, the function applies a learnable, tissue-specific affine transformation (with distinct parameters per site type, strand, and organism). The main `splice_junctions` function computes the predicted count for each potential junction via an inner product (Einsum) between the corresponding processed donor and acceptor embeddings, followed by a Softplus activation to ensure positive values.

The training loss for splice junction predictions (`junctions_loss`) combines multiple terms. Two cross-entropy terms compare predicted and target count ratio distributions. These ratios represent conditional splicing probabilities. The first term evaluates the distribution of acceptor site usage for each donor, akin to a Percent Spliced In from the 5' site (PSI5) perspective. This is achieved by normalizing counts over all acceptors for each donor (i.e., $axis = 1$, effectively $P(\text{Acceptor} \mid \text{Donor}, \text{Tissue})$). The second term evaluates the distribution of donor site usage for each acceptor, akin to a PSI3 perspective. This normalizes counts over all donors for each acceptor (e.g., axis=0, effectively $P(\text{Donor} \mid \text{Acceptor}, \text{Tissue})$). Additionally, two Poisson loss terms compare the marginal sums: one compares the total predicted and target counts summed across all acceptors for each donor (e.g., $axis = 1$), and the other sums across all donors for each acceptor (e.g., $axis = 0$). For numerical stability of the Poisson loss term calculation, the sums of the target counts undergo an additional `soft_clip` operation. The final loss is a weighted combination of the cross-entropy and Poisson components. This entire loss calculation is performed independently for positive and negative strand predictions using their respective splice sites and targets. It is specified by the pseudocode:

```
def soft_clip(x: Array) -> Array:
  return Where(x > 10.0, 2 * Sqrt(x * 10.0) - 10.0, x)

def multinomial_cross_entropy(
  x: Array[D, A, N_tissues], targets: Array[D, A, N_tissues], axis: int
) -> Array[]:
```

```
  pred_ratios = (x + 1e-7) / (x + 1e-7).sum(axis=axis, keepdims=True)
  target_ratios = (targets + 1e-7) / (targets + 1e-7).sum(
  axis=axis, keepdims=True)
  return - (targets * Log(pred_ratios)).sum()

def poisson_loss(
  x: Array[D, A, N_tissues], targets: Array[D, A, N_tissues], axis: int
) -> Array[]:
  sum_pred = x.sum(axis=axis)
  sum_targets = soft_clip(targets.sum(axis=axis))
  return (sum_pred - sum_targets * Log(sum_pred + 1e-7)).sum()

def junctions_loss(
  x: Array[D, A, N_tissues], targets: Array[D, A, N_tissues]
) -> Array[]:
  ratios_loss = (multinomial_cross_entropy(x, targets, axis=0) +
      multinomial_cross_entropy(x, targets, axis=1))
  counts_loss = (
      poisson_loss(x, targets, axis=0) + poisson_loss(x, targets, axis=1))
  return 0.2 * ratios_loss + 0.04 * counts_loss
```

Training

AlphaGenome was trained in a multi-task setting to jointly predict genomic features across both human and mouse reference genomes. We use two training strategies: the first is pre-training, where a model learns from experimental data, and the second is distillation, where a model is trained to reproduce the predictions of an ensemble of pre-trained models. Model architecture is unchanged between training regimes.

Pre-training

When training from data, at every step we sampled contiguous 1 Mb intervals from the training set along with the corresponding experimental data for all model outputs. The training, validation, and test sets for these pre-training runs strictly followed the Borzoi-defined cross-validation folds. Borzoi's original target intervals of 196Kb were extended to 1 Mb input windows for AlphaGenome, and any validation or test intervals that overlapped with training windows after extending were excluded (see Training Data).

We employed two forms of data augmentation during training. First, we applied a shift augmentation, where each sampled interval was shifted by a distance sampled uniformly at random from -1024 to +1024 bp. Second, reverse complementation was applied to the input sequence and corresponding outputs with a 50% probability.

Sequence Parallelism Pre-training utilized TPU v3 hardware, employing sequence parallelism to distribute the computation for each 1 Mb input interval across 8 TPU cores. AlphaGenome's architecture, which alternates between local convolutional processing at high resolutions and global transformer-based processing at lower resolutions, is particularly amenable to this strategy.

For the sequence encoder, the 2^{20} bp input (1 Mb) is divided into 8 sub-sequences, each of length 2^{13} bp. To handle convolutional operations efficiently with minimal cross-device communication, each sub-sequence is extended with a 1024 bp copy of sequence from its neighbors on both sides before being processed by its assigned TPU core. After the encoder converts the input to 128 bp resolution embeddings, 8 embedding vectors (corresponding to the 1024 bp overlap) are trimmed from each end of the processed sub-sequences. This overlap/trimming strategy ensures that the encoder's receptive field

is accounted for (each 128 bp output bin embedding vector is influenced by an additional context of 513 bp to either side) and yields results nearly identical to non-parallel execution. A minor deviation is caused by batch normalization, which requires communication across all devices to aggregate statistics and slightly alters results due to double-counting the overlapping regions (2048 bp from each subsequence are double counted in the variance calculation). However, we observed no practical impact on model performance from this effect.

Processing the transformer tower and pairwise blocks under sequence parallelism requires more inter-core communication compared to the encoder's convolutional layers. For instance, computing multi-query attention does not require communicating the queries (q) but requires gathering the key (k) and value (v) tensors from all 8 cores onto each core. The pairwise activations are distributed along only the first sequence axis; this allows optimizations, such as only gathering the k tensor (and not the q tensor) during the `sequence_to_pair` operation.

Sequence parallelism for the sequence decoder mirrors the encoder's strategy but operates in reverse. The 128 bp resolution embeddings entering the decoder on each core are first extended by concatenating 8 embedding vectors (corresponding to 1024 bp) from neighbors on each side. After the decoder upsamples these to 1 bp resolution, the overlapping regions (1024 bp on each side) are removed from the final output sub-sequences. Subsequent track prediction heads operate independently on the 1 bp output of each core, and the final loss computation and gradient updates are aggregated across all devices, akin to standard data parallelism.

Model Training Parameters In pre-training and distillation for the fold-specific and *all-folds* models, we trained the model using the AdamW[92] optimizer with default hyperparameters ($\beta_1 = 0.9$, $\beta_2 = 0.999$, $\epsilon = 10^{-8}$) and a weight decay of 0.4. Each gradient step processed a batch size of 64 samples using 8-way sequence parallelism, requiring 512 TPUv3 cores, with pre-training runs typically completing in approximately 4 hours. Training proceeded for a fixed duration of 15,000 steps without early stopping. The learning rate followed a schedule with a linear warm-up from 0 to 0.004 over the first 5,000 steps, followed by a cosine decay to 0 over the remaining 10,000 steps. The number of steps was selected to balance expected model performance across both reference genome prediction tasks and zero-shot variant effect prediction tasks, as evaluated on validation data subsets.

Using this protocol, we trained a model for each of the four cross-validation folds (*fold0*, *fold-1*, *fold-2*, and *fold-3*), evaluated on their respective held-out reference genome validation sets. Following best practice, final test set performance was assessed only once, after all dataset choices and model training runs were finalized. Additionally, 64 models were trained using all available reference genome intervals (*all-folds*). As these models lack a dedicated reference genome holdout set, they were not used for track prediction evaluations, and they serve as the teacher ensemble for distillation.

Distillation

Model distillation begins with the same 1 Mb interval sampling and 50% reverse complement augmentation strategy used during pre-training. At this stage, we introduce additional input perturbations to increase the diversity of input sequences. Firstly, 4% of the nucleotides in each input sequence are randomly mutated by substituting with a nucleotide chosen uniformly at random. Secondly, we apply structural variations: insertions (using random sequences), deletions, and inversions. The number of such structural variations per 1 Mb sequence is sampled from a Poisson distribution ($\lambda = 1.0$), and the length of each variation is chosen uniformly from the range [1, 20] base pairs.

Distillation training was performed without sequence parallelism across 64 NVIDIA H100 GPUs, with a batch size of 64 (effectively one sample per GPU). Each GPU loaded a different frozen teacher model from the pool of 64 pre-trained *all-folds* models. For each GPU, an interval is randomly sampled and predictions were generated using its assigned frozen teacher model. The student model's parameters were replicated

across all devices and it was then trained using the teacher predictions as targets by minimizing the same set of loss functions used during the pre-training phase. By averaging gradients computed against different teachers, this setup provides a computationally efficient stochastic approximation of training the student against the expected predictions of the full teacher ensemble, without needing to run all teachers for every sample.

Model Training Parameters The distillation process ran for 250,000 steps, taking approximately 3 days. We used the AdamW optimizer with default parameters and a reduced weight decay coefficient of 0.04 compared to pre-training. The learning rate schedule consisted of three phases: a linear ramp-up to 0.002 over the initial 5,000 steps, a constant phase at 0.002 for the next 120,000 steps, and finally a cosine decay down to 0 over the concluding 125,000 steps.

Benchmarking Against Existing Methods

AlphaGenome's performance was benchmarked against several existing state-of-the-art sequence-to-function models. These included general-purpose models like Enformer[1] and Borzoi[2], as well as models specialized for specific tasks: ProCapNet[9] for PRO-cap signal prediction; SpliceAI[4], DeltaSplice[93], Pangolin[11], and Splam[94] for splicing predictions; Orca[14] for contact map prediction; and ChromBPNet[29] for local chromatin feature prediction. For these comparisons, evaluations were performed on held-out human test intervals, using metrics appropriate for each prediction task. Pearson correlation coefficient (Pearson *r*) was used for quantitative coverage-based tracks, while the Area Under the Precision-Recall Curve (auPRC) was used for binary classification tasks (details and full results in **Supplementary Table 3** and **Supplementary Table 4**).

Publicly available pre-trained models, weights, or source code for these comparison models were utilized as explained in Supplementary Table 8. Specific considerations were applied for certain comparisons to ensure fair evaluation. When scoring variants with SpliceAI, Pangolin, and DeltaSplice, predictions were made by considering a 2,000 base pair window on either side of the variant, overlapped with the gene mask for this region. For performance comparisons between AlphaGenome and ChromBPNet, evaluations were conducted exclusively on experimental tracks with directly matching ENCODE accessions. For ATAC, these were ENCSR200OML (IMR-90) and for DNase: ENCSR000EOT (K562), ENCSR149XIL (HepG2), ENCSR000EMT (GM12878) and ENCSR477RTP (IMR-90).

Genome Track Evaluation

We evaluated AlphaGenome's ability to predict various genomic features using several metrics tailored to the nature of each output type.

Correlation for Continuous Tracks

Concordance between predicted and observed continuous track signals (such as those for ChIP-seq, DNase-seq, ATAC-seq, CAGE, and PRO-cap) was primarily measured using the Pearson correlation coefficient (r). For a given track and a held-out test interval, Pearson r was calculated between the vector of predicted values and the vector of observed values across all corresponding genomic bins within that interval. The distributions shown in **Fig. 2c** represents the Pearson r values calculated for all tracks within a specific assay group (e.g., all TF ChIP-seq tracks) and organism (e.g., human and mouse) across all held-out test intervals. The average Pearson r for each group (shown as text and circle) is the mean of these individual track correlations.

Gene Expression Correlation

Pearson correlation (r) was also computed between observed and predicted gene expression values on held-out test intervals. Gene expression values for both observed and predicted tracks were calculated as the log-transformed mean read coverage across all annotated exons for a given gene, using GENCODE version 46 annotations and considering strand matching. To avoid duplicate genes across test intervals, we only considered genes with at least 50% of their exons falling within a test interval. Three specific correlation approaches were used:

- *Raw; Across Genes (**Fig. 2d**, left)*: For each cell type/track, correlation was computed across all genes between their predicted and observed log-transformed expression values.
- *Normalized; Across Genes (**Fig. 2d**, middle):* For each track, log-transformed expression values were quantile normalized across genes. Then, for each gene, its mean expression across all tracks was subtracted. Correlation was then computed across genes between these normalized predicted and observed values within each track.
- *Normalized; Across Tracks (**Fig. 2d**, right):* Using the same quantile-normalized, gene-mean-centered data as above, correlation was computed *across* tracks/cell types for each gene separately, assessing per-gene prediction consistency over different cellular contexts.

Alternative Polyadenylation

To assess the model's understanding of alternative polyadenylation site usage, we compute polyadenylation-centric coverage ratios (COVR), as defined in the Borzoi paper. These COVR values quantify the relative usage of the distal versus proximal polyadenylation sites for a gene as annotated by PolyADB[95], restricted to sites no further than 50bp from the gene boundary. We compare AlphaGenome's and Borzoi's predicted COVR values on held-out test sites against the true COVR values derived from tissue-pooled GTEx data.

Splicing Prediction Performance

AlphaGenome's performance on various splicing-related track prediction tasks was assessed using specific test sets, metrics, and comparisons depending on the specific output type.

Splice Site Classification The model's ability to correctly identify and classify splice sites was primarily evaluated using the auPRC metric. For this task, we evaluated separately with true labels derived from RNA-seq observed splice sites and those annotated in GENCODE GTF files. At each relevant genomic position, the model predicted probabilities for four positive splice site classes: Donor site on the plus strand (Donor+), Acceptor site on the plus strand (Acceptor+), Donor site on the minus strand (Donor-), and Acceptor site on the minus strand (Acceptor-). The test sequences were split into batches with input sequence length 1M base pair. A separate auPRC was computed for each of these four classes, comparing predicted probabilities against true labels, and the overall reported performance is the average auPRC across them.

For specific comparisons against models like SpliceAI and DeltaSplice, AlphaGenome's performance (using the ensemble of four *fold-1* models, chosen for maximal test set overlap with these peer models) was assessed on test intervals derived from human chromosomes 1, 3, 5, 7, and 9 for consistency. Pangolin was not evaluated for this task as it does not predict separately donors or accepters, only splice sites in general.

Splice Site Usage Prediction The evaluation of splice site usage (SSU) was conducted using Pearson correlation coefficients (r). These were computed between the vector of AlphaGenome's predicted SSU

values and the vector of observed SSU values (derived from RNA-seq) for each tissue across held-out test intervals, treating each tissue's SSU profile as a distinct track.

For comparative benchmarking (e.g., against DeltaSplice), the same held-out test intervals and SSU predictions FROM AlphaGenome *fold-1* were used. Annotated SSU values were derived from RNA-seq. Comparisons with Pangolin for SSU were not performed due to differing SSU definitions and due to Pangolin not using its SSU prediction head for variant effect prediction.

Splice Junction Prediction The model's performance in predicting splice junctions was evaluated through three distinct approaches on the held out test intervals of *fold-1* with an ensemble model trained with *fold-1* training data.

1. *Classification of true vs. false junctions:* This task assessed the model's ability to distinguish true junctions (defined as donor-acceptor pairs with supporting RNA-seq read counts after the filtering steps described in the data section) from the vast majority of false junctions (defined as donor-acceptor pairs with no RNA-seq supporting). This task is challenging since only a small set of donor/acceptor pairs are true within 1M base range. The model's predicted junction counts were the classification scores. For Splam, since it does not directly predict a junction score, we used the min of the donor and acceptor splice site probabilities as a prediction for the junction score for each splice junction. An auPRC was computed for each tissue independently by comparing flattened matrices of these predicted scores against the corresponding flattened binary ground truth labels. The final reported performance is the average auPRC across all tissues.
2. *Quantitative prediction of junction counts:* The accuracy of predicting the strength of junction usage was assessed using the Pearson correlation between $\log(1 + x)$ transformed predicted junction counts and $\log(1 + x)$ transformed measured junction counts (interpreted as junction strength). This correlation was only computed over junctions that had non-zero read counts in the ground truth test set data.

Prediction of PSI5 and PSI3 levels: The model's ability to predict local splicing choices was further evaluated by comparing measured and predicted PSI5 and PSI3 levels using Pearson correlation. These metrics are defined as: PSI5(D,A)=n(D,A) /A'n(D,A'), PSI3(D,A)=n(D,A) /D'n(D',A), where n(D,A) is the number of split reads supporting the specific junction from donor D to acceptor A, A'n(D,A') is the total number of split reads supporting all splice junctions originating from donor D, and D'n(D',A) is the total for all splice junctions ending at acceptor A. The PSI correlation reported in (**Extended Data Fig. 2**) are from the test intervals in chr2 only.

Together, these three approaches provide a multifaceted assessment of AlphaGenome's ability to accurately predict both the presence and quantitative strength of splice junctions, as well as local splice isoform choices.

Contact Maps Performance

Our evaluation of contact map predictions was designed to closely mirror the evaluations performed on the Orca 1 Mb model[14]. The primary metric for assessing performance was the mean Pearson correlation coefficient (r), where individual Pearson r values were calculated between AlphaGenome's predicted contact scores and the observed experimental contact scores within each held-out genomic interval in a specific human tissue type. The held-out test intervals were defined by intersecting the Orca test split (human chromosomes 9 and 10) with AlphaGenome's cross-validation splits (specifically, those aligned with Borzoi FOLD_0). Human tissue types chosen for this evaluation matched key training samples from the Orca study, specifically the H1-hESC and HFFc6 Micro-C datasets.

To align with Orca 1 Mb model's headline evaluations, genomic intervals for comparison were 1,000,000 bases (1 Mb) in length, divided into 250 bins of 4 kb each (the native output resolution of Orca).

Since AlphaGenome's native output resolution for contact maps is 2048 bp bins over a 1,048,576 bp interval, AlphaGenome's predictions were resized and interpolated to match Orca's 4 kb resolution using bilinear interpolation (`jax.image.resize`).

Furthermore, the H1-hESC - HFF Micro-C *difference* prediction evaluation was re-implemented following the specific protocol detailed in the Orca code repository (Cell 20 of https://github.com/jzhoulab/orca_manuscript/blob/main/Model_performance.ipynb). This difference analysis was performed over the same held-out chromosomal splits mentioned above.

Benchmarking Against Enformer Track Predictions

To facilitate a direct and fair comparison with Enformer[1], a specialized AlphaGenome model was trained using the identical training sequence intervals as the original Enformer model. This AlphaGenome variant incorporated a dedicated "Enformer head" to predict Enformer's target tracks at their native 128 bp resolution (while the model's other heads predicted standard AlphaGenome outputs). For this Enformer-specific head, loss was calculated exclusively over the central 114,688 bp region corresponding to Enformer's target width, using AlphaGenome's full 1 Mb input. To prevent data leakage, test set intervals whose 1 Mb AlphaGenome input windows overlapped these Enformer training intervals were excluded from evaluation.

Benchmarking Against Borzoi Track Predictions

For a direct comparison with the Borzoi *fold-1* model[2], an AlphaGenome model, which was initially trained on the identical Borzoi *fold-1* data split, underwent fine-tuning. This fine-tuning process involved augmenting the AlphaGenome architecture with two additional heads to mirror Borzoi's outputs.

The first head aggregates AlphaGenome's 1 bp embeddings into 32 bp embeddings and makes predictions matching the Borzoi tracks (7,611 human and 2,608 mouse). This head is trained on Borzoi's TFRecords dataset (original resolution and scaling) to allow for a direct comparison with the published Borzoi model. This head is used for metrics reported in **Fig. 1d** where we compare against Borzoi at 32 bp resolution.

The second RNA-seq head is trained on the same RNA-seq tracks as Borzoi but reprocessed at 1 bp resolution and without any Borzoi specific scaling. When comparing this head against Borzoi, we unscale and repeat Borzoi's predictions 32 times (to equal 1 bp unscaled predictions). This head is used for metrics reported in **Fig. 1d** where we compare against Borzoi at 1 bp resolution.

We validated the approach of upsampling and scaling the additional RNA-seq head to match Borzoi by applying the same procedure to the training data. AlphaGenome's base-resolution data was aggregated to a 32 bp resolution and Borzoi's original scaling methodology was applied. Compared to Borzoi's provided TFRecord files, we achieved high concordance (0.988 average Pearson r correlation) . This comparison excluded unmappable regions, as flagged by the 'umap' entry in the Borzoi data examples. Finally, in the Borzoi's and AlphaGenome's RNA-seq comparison at 1 bp resolution, we only include the tracks for which the Pearson r correlation is larger or equal than 0.99 and exclude the unmappable regions.

Relative Improvement Metric

To contextualize performance gains, particularly when comparing AlphaGenome against various models and random baselines across different tasks, we calculated a Relative Improvement score (as shown in **Fig. 1d** for tracks and **Fig. 1e** for variant effect prediction). This metric is defined by the formula:

$$\textit{Relative Improvement (\%)} = 100 \times \frac{\text{alphagenome\_metric} - \text{comparison\_metric}}{\text{comparison\_metric} - \text{random\_baseline\_metric}}$$

In this formula:

- `alphagenome metric` refers to the performance score (e.g., auPRC, Pearson *r*) achieved by AlphaGenome.
- `comparison_model_metric` is the performance score of the specific model AlphaGenome is being compared against (e.g., Borzoi, Enformer) on the identical task and dataset.
- `random_baseline_metric` represents the expected performance of a random predictor, chosen appropriately for the primary evaluation metric being used.

Random baseline metrics were computed as follows:

- For tasks evaluated with either Pearson or Spearman correlation, the `random_baseline_metric` was set to 0.
- For tasks evaluated with auPRC, the `random_baseline_metric` was set to the fraction of positive instances in the dataset (i.e., the class prevalence).
- For tasks with class-balanced datasets evaluated with auROC, the `random_baseline_metric` was set to 0.5.

This relative improvement metric normalizes the gain achieved by AlphaGenome over a comparison model by the amount that comparison model itself improved over a random baseline. Detailed individual comparisons that contribute to these relative improvement calculations are presented in **Supplementary Table 3** and **Supplementary Table 4**.

For accessibility variants, we evaluated AlphaGenome and the SOTA models on a number of highly similar tasks, specifically five directionality benchmarks and three causality benchmarks, as described in the section Chromatin accessibility variants & bQTLs. We outperform SOTA in each individual case. However, to present a consolidated number in **Fig. 1**, we computed the average relative improvement across these benchmarks.

Variant evaluations

Variant scoring

Variant scoring aims to distill AlphaGenome's multifaceted genomic predictions into a single, informative scalar value for each genetic variant, representing its predicted biological impact. This score quantifies the difference in predicted features (e.g., chromatin accessibility, TF binding, gene expression, splicing outcomes) between sequences carrying the reference (REF) and alternative (ALT) alleles. The methodology for deriving this score is critical, involving choices about the genomic region analyzed, the function used to aggregate allelic differences or activity levels across this region, and any applied transformations (e.g., log ratios).

Based on optimization against downstream evaluation tasks, we established a set of 19 recommended variant scoring configurations covering diverse genomic modalities (details in Supplementary Table 9, with illustrated workflows in Supplementary Fig. 13). These scorers primarily fall into two categories – those using masks centered on the variant (*center mask scorers*) and those using masks derived from gene annotations (*gene mask scorers*) – supplemented by specialized scorers for features like contact maps and specific splicing or polyadenylation events.

Scoring pipeline overview

Before detailing scorer-specific configurations, we first outline the overall pipeline for center mask and gene mask variant scorers.

1. Allele-Specific Predictions AlphaGenome generates two sets of genomic predictions centered on the variant: one for the DNA sequence containing the REF allele and another for the sequence with the ALT allele. The REF allele sequence used for comparison is either the standard reference genome allele or, when specified, the specific REF allele defined by the evaluation dataset.

2. Indel Alignment (Optional) Following previous work[4], for insertion or deletion (indel) variants , the ALT allele's prediction profile is aligned to the REF allele's coordinate space. Inserted bases are summarized by taking the maximum value over the inserted segment, while deleted bases are treated as having zero signal in the ALT context, thereby enabling consistent positional comparisons.

3. Mask Application and Spatial Aggregation A spatial mask is applied to focus on the relevant genomic regions within the 1 Mb input window.

- For **center-mask approaches**, a fixed-width window (e.g., 501 bp for local marks like ATAC-seq or DNase-seq; 2001 bp for broader histone modifications) is defined around the variant.
- For **gene-mask approaches**, windows for each gene are defined by gene annotations (e.g., gene bodies, exons, or TSS locations from a GTF file).

Predictions across the spatial windows are then aggregated using sum, mean, L2 norm, or taking the maximum across all REF and ALT signals. This results in a single output per track (center-mask) or per track and gene (gene-mask) for both the REF and ALT allele.

4. Transformation and Allelic Comparison Mathematical transformations (e.g., log, log1p, absolute value) may be applied to stabilize variances or emphasize particular types of effects, either before or after spatial aggregation, depending on the specific scorer configuration. Finally, the REF and ALT predictions are compared to yield a final score per track (center-mask) or per track and gene (gene-mask).

5. Track Aggregation (Optional) After a score is derived for each output track (e.g., per cell type or assay target), a final step may aggregate these scores (e.g., by taking the maximum absolute score across relevant tracks or selecting a specific track of interest) to produce a single scalar value representing the variant's overall predicted impact for that specific scoring configuration.

Core Differential Variant Scorers We provide the following core scorers for quantifying the impact of variants by comparing REF and ALT allele predictions:

- *Gene expression (using RNA_SEQ outputs):* To score effects on RNA abundance (from RNA_SEQ model outputs), predictions are aggregated over annotated gene regions (mean signal across exons). The variant score is the log-fold change of this aggregated gene expression level between the ALT and REF alleles (Supplementary Fig. 13b). Only genes fully contained within the input interval are scored.
- *Splicing – Site-based Impact (SPLICE_SITES AND SPLICE_SITE_USAGE outputs):* Variant effects on predicted splice site probabilities (from SPLICE_SITES outputs) or usage percentages (from SPLICE_SITE_USAGE outputs) are evaluated within gene boundaries. The score is the maximum absolute difference in these predicted values between the REF and ALT alleles across all positions within a gene body (Supplementary Fig. 14a). Only genes overlapping the variant are scored.

- *Splicing – Junction-based Impact (SPLICE_JUNCTIONS output):* To assess effects on specific splice junctions, scores are derived from the change in predicted junction counts or strengths between REF and ALT alleles, after a log transformation. Splice junction prediction for the REF and ALT are based on the same set of splice sites. In contrast to the model training stage, the splice sites used are a union of those observed from RNA-seq data (described in splice site training data), those predicted from the reference sequence, and those predicted from the alternative sequence. The splice site probability is an element-wise maximum of the three sources (RNA-seq splice sites are labeled as probability 1 to ensure they have the highest priority). For indels, an alignment step is performed before taking the element-wise maximum. Only splice sites within the gene body of the genes overlapping the variant are considered. For each variant, we consider a maximum of 256 donor or acceptor sites. To score the variant for each tissue, the maximum absolute log fold change of the junction score per junction across all junctions within a gene is reported (Supplementary Fig. 14b).
- *Polyadenylation site usage (using RNA_SEQ outputs):* Variant effects on polyadenylation (from RNA_SEQ model outputs) are scored using a method analogous to Borzoi's paQTL approach. This gene-based score compares predicted RNA coverage at annotated 3' cleavage junctions for REF and ALT alleles. It reflects the maximum change in relative usage between any two potential polyadenylation sites (one considered proximal, one distal) within a gene's 3' UTR, expressed as an absolute log2 fold change of these isoform ratios (Supplementary Fig. 13c). Only genes with 80%+ of their polyadenylation sites within the input interval are scored.
- *Local chromatin accessibility and activity (using ATAC, DNASE, CAGE, PRO_CAP outputs):* For assays measuring local chromatin state or transcriptional start site (TSS) activity, variant effects are assessed using a mask centered on the variant (501 bp). The score represents the log2 fold change of total signal summed within the window for the ALT allele versus the REF allele (e.g., log2((sum(ALT) + p) / (sum(REF) + p)), Supplementary Fig. 13a).
- *Transcription factor binding and histone modifications (CHIP_TF and CHIP_HISTONE outputs):* For these assays, variant effects are assessed using a 501 bp centered window for CHIP_TF and 2001 bp for CHIP_HISTONE to account for the broader nature of some histone marks. The score represents the log2 fold change of summed ALT vs. REF signals.
- *Contact Maps (CONTACT_MAPS output):* For variants affecting 3D chromatin contacts, a method similar to that used by Orca is employed for SNVs. This calculates the mean absolute difference between REF and ALT contact map predictions for all interactions involving the single genomic bin containing the variant and other bins within a defined local window (e.g., 1 Mb).

This suite of scoring strategies allows for a multifaceted interpretation of variant effects across various genomic functions predicted by AlphaGenome.

Active Allele Scoring In addition to the differential scores described above, we also provide scoring configurations that capture the *absolute activity level* associated with an allele, rather than quantifying the change between REF and ALT. This is calculated by taking the maximum of the aggregated signals from the REF and ALT alleles over the central window (center-mask scorers) or gene region (gene-mask scorers). Active allele scorers provide insight into the overall strength of a regulatory element harboring the variant by reporting the maximum of the summed signals for the REF and ALT alleles within the defined centered window.

Composite splicing variant scorer To provide a comprehensive measure of a variant's overall impact on splicing, predictions from the splice junction, splice site, and splice site usage heads are integrated. Splice site and usage scores inherently range from 0 to 1. Splice junction scores, representing absolute log-fold changes, predominantly exhibit values up to approximately 5 in typical scenarios. Therefore, to ensure comparable contributions from each component, the splice junction scores are first normalized by

dividing by 5. The final aggregated splicing score is then the sum of this normalized junction score and the original splice site and usage scores.

Calibration Methodology

To make raw variant scores more interpretable and comparable across different assays and genomic contexts, we implemented an empirical quantile calibration procedure against a background set of common genetic variants. This variant set comprised 348,126 common human SNPs from chromosome 22 with MAF>0.01 in any of gnomAD's ancestral groups (gnomAD v3). We used this set of variants to estimate a background score distribution for each variant scorer and track. We can then derive a 'quantile score' for any arbitrary variant (using the procedure below), representing its percentile rank (or signed percentile rank) within the distribution of scores from common variants for a given scorer and track. This provides a measure of predicted impact that is standardized to the same scale across variant scorers and tracks. We used common variants as they are more likely to be depleted for high-impact variants compared to random genomic variants, and are less likely to be biased to a particular use-case or modality.

The calibration process was as follows:

1. *Raw score generation:* The full set of 19 recommended AlphaGenome variant scorers was run on these 348,126 background variants to generate their raw effect scores.
2. *Quantile computation:* For each unique combination of scorer and output track (e.g., a specific epigenetic mark in a particular cell type), empirical quantile probabilities were computed from the distribution of raw scores. This was achieved by stratifying the raw scores into 1,000 bins. To enhance precision at the extremities of the score distributions (i.e., for very strong or very weak predicted effects), the spacing of these bins was determined using a sigmoid function.
3. *Handling of invalid scores:* Any Not-a-Number (NaN) scores were ignored during this process. NaN scores arise in scenarios such as when a gene-based scorer is applied to a variant outside of any relevant gene mask (e.g., a splicing scorer applied outside of a gene mask, such as for an intergenic variant) or when predictions are made for genes on the incorrect strand relative to the scorer's expectation.
4. *Minimum thresholding:* Quantile probabilities were thresholded at 1×10^{-5} (e.g., values below this were floored to this minimum) to mitigate the impact of extreme outliers, and due to the number of variants in the background set, $O(1e5)$.
5. *Adjustment for signed scores:* For signed variant scores (which indicate effect direction like up-regulation or down-regulation), their [0,1] quantile probabilities – derived directly from the rank order of the original signed raw scores – are linearly transformed to a [-1,1] range. This rescaling ensures the calibrated score reflects the original directionality: for instance, the 0th percentile (representing the most negative raw scores) maps to -1, the 50th percentile (raw scores around zero) to 0, and the 100th percentile (most positive raw scores) to +1.
6. *Tie-breaking:* Occasionally, multiple background variants yielded identical raw scores, such that the same score would map to multiple quantiles. To enforce unique ranks, a tie-breaking procedure was applied: variants in a tied group were randomly (with uniform distribution) assigned a distinct quantile score, spanning the percentile range they collectively occupy.

In Silico *Mutagenesis (ISM) for Contribution Scores*

To interpret which nucleotides in a sequence of interest contribute most to AlphaGenome's predictions for a specific genomic feature, we employed an *in silico mutagenesis* (ISM) approach. This method estimates a contribution score for each position in the sequence.

The procedure is as follows:

1. *Systematic single nucleotide variant (SNV) generation:* For each position within the L-length input sequence, all three possible alternative nucleotide substitutions are generated. This creates a comprehensive set of SNVs covering every possible single base change from the original sequence.
2. *Variant scoring:* Each of these generated SNVs is then scored using a chosen AlphaGenome variant scorer (as described in the Variant Scoring section) to obtain a single scalar value representing the predicted impact of that specific mutation on a particular model output (e.g., an epigenomic track or gene expression value in a specific cell type).
3. *Construction of an effect matrix:* The resulting scalar scores for the three alternative mutations at each position are organized into an $L \times 4$ matrix, where L is the sequence length and the 4 columns correspond to the four possible nucleotides (A, C, G, T). For each position, the entries for the three alternative (mutant) nucleotides are populated with their respective variant scores, while the entry for the original reference nucleotide at that position has a score of 0 (as it represents the baseline against which mutations are compared).
4. *Mean-centering:* To normalize these effects, for each position i in the sequence, the mean of the three variant scores (i.e., the scores associated with mutating the original base to its three alternatives) is calculated. This mean is then subtracted from all four entries (A, C, G, T) in the matrix at that position i. Consequently, after this step, the matrix entry corresponding to the original reference base at position i now holds the value $0 -$ mean_of_alternative_scores.
5. *Computing the final contribution scores*. The final contribution score for each nucleotide in the original input sequence is then taken directly from this mean-centered $L \times 4$ matrix by selecting the value that corresponds to the actual reference base at that position. These L resulting scores (one per position) represent the influence of each reference base relative to the average of its alternatives and are typically visualized as a contribution score track or “saliency map”.

These per-position contribution scores are then visualized as sequence logos, where the height of each letter in the original reference sequence is scaled by its calculated contribution score. The value thus obtained for each position quantifies the specific contribution of the reference base to the predicted outcome, relative to the average effect of substituting it with alternative nucleotides.

Signed values are meaningful for two-sided scorers. For variant scorers producing strictly non-negative scores, we always report non-negative values corresponding to the mean of alternative scores.

Comparative ISM for Variant Effect Interpretation Furthermore, to specifically investigate how a genetic variant might alter local sequence motifs, this ISM procedure is applied independently to both the reference (REF) sequence and the sequence containing the alternative (ALT) allele. The resulting REF and ALT contribution score profiles (as sequence logos) are then compared. This comparison is powerful because the single nucleotide change in the ALT sequence can alter the model’s interpretation of the entire local region, not just the mutated base itself. This comparative approach is particularly useful for identifying if a variant potentially disrupts an existing regulatory motif present in the REF sequence or, conversely, creates a novel motif in the ALT sequence.

Test-time augmentation

A test-time augmentation (TTA) strategy involving strand averaging was employed when evaluating performance on variant, unaggregated, and aggregated track evaluations. Predictions were generated for both the forward DNA sequence and its reverse complement. For track evaluations, performance metrics were computed on the average of the two predictions. For variant evaluations, variant effect scores were computed independently based on each stranded prediction, and the final reported score for a variant was the average of these two. This approach generally improved the performance metrics observed for non-distilled models, but did not consistently enhance the performance of AlphaGenome’s distilled model evaluations.

Chromosome Splits for Variant Benchmarks

For zero-shot evaluations, each variant was assigned to either the validation or test set, according to the following chromosome split: variants in chromosomes 1, 2, 4, 5, 7, 8, 10, 11, 13, 14, 15, 17, 20, 22, X were used as the validation set to inform model design and for variant scoring strategy development, whereas variants in chromosomes 3, 6, 9, 12, 16, 18, 19, 21 were used as the test set, on which all final performance metrics are reported.

For supervised evaluations, we used the following split: chromosomes 1, 4, 7, 8, 10, 13, 15 were used as the training set, chromosomes 2, 5, 11, 14, 17, 20, 22, X as the validation set, and chromosomes 3, 6, 9, 12, 16, 18, 19, 21 as the test set. Supervised models were developed on the basis of the training and validation set, with final performance metrics reported on the test set.

Splicing Variant Benchmarks

To assess AlphaGenome's ability to predict the functional consequences of variants on splicing, we utilized several curated datasets and benchmarks, each focusing on different aspects of splicing regulation. Different models were evaluated on these benchmarks using suitable variant scorers corresponding to their supported output types. AlphaGenome was evaluated using composite splicing scores and splice junction scores. Borzoi was evaluated using RNA-seq scores computed as the maximum absolute difference in normalized coverage across the gene span, as in the original study[2]. SpliceAI was evaluated using splice site prediction scores. DeltaSplice and Pangolin were evaluated using splice site usage scores.

1. Splicing Quantitative Trait Loci (sQTLs) Splicing QTLs (sQTLs) are genetic variants that influence gene splicing, often by altering isoform abundance. We utilized a curated dataset previously developed for the Borzoi model based on the eQTL catalogue[96]. This dataset comprises 21,514 fine-mapped causal sQTLs and tissue pairs (positive examples) and 21,514 carefully selected distance-matched non-sQTL variants and tissue pairs (negative controls) across 49 GTEx tissues.

The task was to distinguish between these causal sQTLs and the matched negative controls, evaluated using auPRC per tissue.

2. ClinVar Variants ClinVar variants were downloaded from ClinVar ftp portal (`https://ftp.ncbi.nlm.nih.gov/pub/clinvar/vcf_GRCh38/`, release date 20250323). Only variants that meet the following criteria were considered:

1. Annotated as one of 'Pathogenic', 'Likely_Pathogenic', 'Benign', 'Likely_Benign'.
2. Has at least one review star.
3. In autosome
4. Annotated either intronic, synonymous, or missense

All 'Pathogenic', 'Likely_Pathogenic' variants are labelled as 1 and all 'Benign' and 'Likely_Benign' variants are labelled as 0. ClinVar variants are divided into three categories based on molecular consequences from the ClinVar VCF file and distance to the nearest splice site:

1. *Deep intronic and deep synonymous*: variants annotated as 'intronic' and >6 bp from the closest splice site, or variants annotated as 'synonymous' and >3 bp from the closest splice site. Since the majority of intronic and and synonymous variants are benign, a further sampling step was performance for 'clinvar_splicing'. For each pathogenic/likely_pathogenic variant, up to 100 benign/likely_benign were sampled randomly. All benign/likely_benign were sampled for the gene if

the negative variant is less than 100 fold of the positive variant. 1,628 positive and 95,269 negative variants are in this category.

2. *Missense variant*: all variants annotated as 'missense' and are predicted by AlphaMissense as 'Likely Benign'. 5,108 positive and 100,031 negative variants are in this category.
3. *Splice site region*: intronic, synonymous, and missense variants that are less than 3 bp from splice sites for exonic variants or less than 6 bp from splice sites for intronic variants. 7,155 positive and 47,354 negative variants are in this category.

3. Splicing Outliers from GTEx Splicing outliers junctions are characterized by highly aberrant splicing patterns (e.g., exon skipping, intron retention, or cryptic splice site usage). Aberrant spliced splicing outliers and the associated rare variants were derived from GTEx RNA-seq data following the description in the AbSplice study[50]. Specifically, splicing outliers were detected with the FRASER 2.0[97] and DROP pipeline (version 1.3.3)[98]. Variant allele frequency was derived from GnomAD release 4.0 (accessed February 2024)[99]. Only GTEx variants with minor allele frequency less than 0.1% and that are present in at most two individuals are considered. Splicing outlier junctions are paired with rare GTEx variants matching the individual and require that the rare variant is at most 250 bp from the aberrant splice junction. We refer to the AbSplice study[50] for more detailed data processing steps. This comprehensive dataset includes over 1 million rare variants of which 5,819 are splicing outliers in one or more GTEx human tissues, annotated for their potential to cause tissue-specific aberrant splicing.

On this benchmark, AlphaGenome was evaluated in two ways on the same test subset of variants:

- *Zero-shot evaluation:* Directly using AlphaGenome aggregated splicing scores and splice junction scores.
- *Supervised evaluation:* Training an Explainable Boosting Classifier model (from the InterpretML python package[100]) and evaluating on the held-out test subset of variants. Features for this classifier included splicing-related variant scores derived from AlphaGenome (scores from splice site, splice site usage, and splice junction predictions, as well as RNA-seq scores computed as the maximum absolute difference in normalized coverage across the gene span[2]). An additional binary feature, indicating whether a splice site is expressed in a given tissue (using a cutoff of 10 reads for the median number of split reads sharing the splice site from GTEx RNA-seq samples), was also included. As baseline, we used the AbSplice ensemble model [50] re-trained and evaluated on the same dataset split as AlphaGenome.

The metric used was auPRC across all GTEx tissues, computed by assigning a tissue to each rare variant: positive variants were mapped to their corresponding GTEx tissue, while negative variants were randomly assigned a tissue according to the positive variants' tissue distribution.

4. MFASS (Massively Parallel Assay of Splicing Sequences) To evaluate predictions against high-throughput experimental measurements of splicing, we used data from the MFASS study[22], a massively parallel reporter assay that tested the exon skipping effects of 27,733 ExAC single nucleotide variants (SNVs) spanning or adjacent to 2,339 exons. Data processing for the MFASS dataset followed the pipeline detailed in the MMSplice paper's code repository (`https://github.com/gagneurlab/MMSplice_paper/blob/master/code/Figure2/MFASS.ipynb`).

Model performance was assessed using auPRC for predicting whether a variant is a "splice disrupting variant" (SDV) or not. SDVs were defined in the MFASS study as variants that change the exon inclusion index by at least 0.5. Variants with mislabelled strand information were removed. The final dataset has 1,040 positive splice disrupting variants and 26,464 negative variants.

To score MFASS variants, we found that using predicted splice site logits instead of probabilities improved the performance of all models, therefore logits are used to score variants in this benchmark

instead of probabilities as in other benchmarks. MFASS measures exon inclusion index. To score MFASS variants, the mean predicted splice site probability and splice site usage differences at the donor and acceptor of the target exon are used to score variants. To score variants with the splice junction head, we compute the predicted junction score differences for all junctions using the donor or acceptor of the target exon, take the max absolute difference for donor and acceptor separately and then take the sum. The aggregate splicing score sums the scores from splice sites, SSU, and splice junctions. Scores across tissues are averaged.

Expression Quantitative Trait Loci (eQTL) Variants

To evaluate AlphaGenome's ability to predict the impact of variants on gene expression regulation, we built upon eQTL benchmarks established by Borzoi[2].

1. eQTL Dataset Preparation For SNP eQTL effect size and sign evaluations, we use a dataset of eQTLs from GTEx v8[17], fine-mapped using the SuSiE method[25]. For these evaluations, only variants with a posterior inclusion probability (PIP) $\geq$ 0.9 were retained. For all reported metrics and counts of eQTLs, we used variants only in the 'test' set chromosomes (see 'Chromosome splits for variant benchmarks' above), which correspond to approximately one third of the fine-mapped variants.

For indel eQTL evaluations, we downloaded the reprocessed and SuSiE fine-mapped GTEx data provided via the EMBL-EBI eQTL catalogue[101]. We filtered this data down to non-SNV variants with PIP $\geq$ 0.9. We excluded variant-gene pairs where the eGene was not annotated in the GTF used throughout our analyses (Gencode V46). The resulting set contains 2645 indel eQTL (variant/gene/tissue triplets), 1535 of which are deletions.

2. Variant Effect Scoring for eQTLs For all zero-shot eQTL evaluations we used the gene-specific RNA-seq scorer, using the ALT and REF alleles as specified for each eQTL in a given evaluation dataset, regardless of whether this REF allele corresponds to the reference genome used. This scorer summarizes the impact of the ALT allele compared to the REF allele by aggregating log fold change in predicted RNA-seq counts over a gene's exons (**Fig. 4a**; Methods). We use the score for the GTEx tissue and gene corresponding to each eQTL in the evaluation dataset. For simplicity, we applied the same scoring strategy to Borzoi's RNA-seq predictions (we note that this yielded very similar results to the author's published best scoring method for eQTLs). For GTEx tissues unavailable in Borzoi outputs, we averaged the scores corresponding to other tissues belonging to the same broad category, as defined by Borzoi[2] (e.g. for 'Brain (Hippocampus)' we used the average of scores from other brain tissues available in Borzoi outputs). For Enformer, a linear layer was fitted to predict the GTEx expression counts at TSSs from the output predicting on Enformer's training intervals. Scores were calculated by aggregating the log fold change in predicted RNA-seq counts over a gene's annotated transcription start site, following the variant scoring strategy as previously published (with the primary adaptation being that input sequences consistently centered the variant, in line with AlphaGenome's standard variant input preparation).

3. Prediction of eQTL Effect Size (Coefficient) To assess the model's ability to predict the magnitude of the effect of known causal variants, we evaluate its performance at predicting an eQTL's effect size (SuSiE 'beta posterior'). This value need not be in the same scale as the predicted effect size, but we expect the ranks and sign to be consistent. Thus, for each GTEx tissue, we compute the Spearman correlation between the predicted variant scores and observed effect size across all variant/gene pairs. We report this Spearman ρ value averaged across all tissues, weighted by the number of eQTLs in each tissue. For this evaluation we used causal eQTLs as described above, consisting of n=17,675 unique variant/gene/tissue combinations, which comprises 6,626 unique variant/gene pairs, and 6,208 unique variants.

4. Prediction of eQTL Effect Direction (Sign) We evaluate the ability of the model to predict whether a causal variant will have a positive or negative effect on gene expression in their causal gene and tissue. An eQTL is designated as "positive" if its beta posterior is ≥ 0 (n=9,759) and "negative" if beta posterior is < 0 (n=7,916). For each tissue, we compute the auROC for the predicted values against the binary sign labels, and report the average auROC across tissues, weighted by the number of eQTLs in each tissue.

5. Zero-Shot eQTL Causality Prediction We evaluated the model's zero-shot ability to differentiate causal from non-causal eQTLs. For this purpose, we downloaded the reprocessed and SuSiE fine-mapped GTEx data provided via the EMBL-EBI eQTL catalogue[101]. We removed all non-SNV variants. We then designated variant-gene pairs with PIP ≥ 0.9 as putatively causal, whereas variant-gene pairs with PIP ≤ 0.01 were designated as non-causal. We excluded variant-gene pairs where the eGene was not annotated in the GTF used throughout our analyses (Gencode V46).

To account for the inherent distance bias of this data (most causal eQTL are close to their target genes, whereas negatives show a more uniform distribution), we performed a distance balancing. Specifically, we computed the log of the absolute distance (bp) between the eQTL variant and the TSS of its target gene (using the median TSS across all transcripts of that gene in the GENCODE V46 GTF). We then divided the range of distances into 10 equal-width bins, and within each bin, randomly downsampled the variant/gene/tissue tuples belonging to the non-causal class to match the number in the causal class. This resulted in 21,948 variant/gene/tissue tuples in the test set, with a class balance of exactly 50%. We measured model performance at distinguishing causal from non-causal eQTLs using the auROC metric, averaged across GTEx tissues weighted by the fraction of eQTLs in each tissue. We also evaluated distance-to-TSS as a predictor using the same metric, and confirmed that it was no longer predictive in the distance-balanced causality dataset.

6. Supervised eQTL Causality Prediction In order to leverage AlphaGenome's multimodal predictions (not just RNA-seq) to differentiate causal from non-causal eQTLs, we trained a random forest model using the absolute values of the variant scores from multiple modalities as features ('Causality (RF)'). We used the same dataset and metric as for zero-shot eQTL causality evaluation (see above), but used a 5-fold cross-validation approach to train and evaluate the random forest. Specifically, we trained a random forest model for each fold only using eQTLs within chromosomes *not* in the test set. We then evaluated the supervised model by making predictions using these trained models for the eQTLs in the held-out test set, and calculated the mean auROC across the 5 folds.

We used the following sets of features, each trained and evaluated separately. For a full list, see the results reported in **Extended Data Fig. 5e**:

- For AlphaGenome, the features consisted of a comprehensive set of variant scores derived from all its output modalities, using the scorer configurations described in the "Variant Scoring" section. For gene-specific variant scorers we used the score corresponding to an eQTL's target gene.
- For Borzoi, features were generated using its published gene-agnostic variant scoring strategy[2], i.e. the L2 norm of log1p-transformed differences computed over a 524,288 bp window across all track outputs. For RNA-seq we used the same scorer as for AlphaGenome.

The Random Forest classifier was implemented using Scikit-learn's `RandomForestClassifier` (version 1.0.2)[102], with a `max_depth` of 5 and default values for other hyperparameters.

7. Stratification of eQTLs by Functional Annotation To investigate the influence of functional context on model performance (**Extended Data Fig. 5c**), we categorized variants based on their overlap with known functional annotations, including:

- Locations of 15 ChromHMM-derived chromatin states (like enhancers and repressed polycomb regions) from ROADMAP Epigenomics[103].
- Locations of candidate *cis* regulatory elements (enhancers, promoters, and CTCF-bound regions) from ENCODE[72,104].
- Locations of enhancers in 5 cell types from the activity-by-contact (ABC) method[105] and enhancers and promoters (tissue-aggregated) from the updated ABC paper[106].
- Variant effect prediction annotation from Ensembl's VEP[107] for variants with a minor allele frequency (MAF) $\geq$ 0.01.
- Gene-level features on expression levels and tissue-specificity of gene expression derived from GTEx.
- We also derive GTF-based features such as whether the variant is in an intron, whether the target gene is protein-coding or a lncRNA etc. from a GTF.

Measuring Sign Prediction Coverage of GWAS

To evaluate AlphaGenome's ability to predict the direction of effect for variants underlying GWAS signals, we developed a pipeline involving curation of GWAS credible sets, derivation of predicted effect signs, calibration against eQTLs, and stratified analysis.

1. Curation of GWAS Credible Sets We accessed GWAS data from Open Targets (data release 22_09, sourced from `bigquery-public-data.open_targets_genetics`). Starting with the Open Targets *variant_disease_credset* table, we first retained only GWAS studies. Subsequently, we selected credible sets exhibiting robust association signals, defined by having at least one variant with genome-wide significant marginal and conditional p-values (marginal $P < 5 \times 10^{-8}$ and conditional $P < 5 \times 10^{-8}$), and where the sum of Posterior Inclusion Probabilities (PIPs) for all variants within the set was 1.

To ensure the independence of these signals, we resolved instances of colocalizing signals (identified through Open Targets' colocalization analysis across studies) by selecting the credible set from the GWAS study that reported the largest number of associated loci genome-wide, using the `num_assoc_loci` field in the Open Targets *studies* table. If credible sets still shared the same lead variant after this step, we prioritized the study where the shared lead variant had the highest PIP, a step that excluded approximately 1,400 credible sets. Finally, we excluded credible sets from any GWAS study (n=73) that were not available in Open Targets' *locus2gene* table. This curation process yielded 18,537 distinct credible sets from 2,476 unique GWAS studies, encompassing 581,248 unique variants.

2. Deriving a Predicted Effect Sign for GWAS Loci To hypothesize the direction of effect for the causal variant underlying a GWAS signal (i.e., a credible set) on its putative causal gene, we utilized AlphaGenome's RNA-seq variant scores. These scores provide predictions for all proximal genes across numerous GTEx tissues, which is advantageous for subsequent calibration using eQTLs. The putative causal gene for each credible set was assigned using Open Targets' *locus2gene* table, selecting the gene with the highest `y_proba_full_model` score.

We first ensure that all variants in the credible set are aligned with respect to sign. That is, the GWAS-signed AlphaGenome score for a variant and gene, $\hat{S}_{vg}$, is the AlphaGenome score (S_{vg}) flipped such that it is aligned with respect to the risk allele (or trait-increasing allele) for a given binary (quantitative) phenotype, p. For example, if the alternative allele is associated with *decreased* risk of diabetes, we flip the sign of the score. This ensures that variants within the same credible set are always oriented such that their sign is with respect to the risk (or trait-increasing) allele.

$$\hat{S}_{vg} = S_{vg} \times \text{sign}(\beta_{vp})$$

Since typically the causal tissue(s) is unknown for a GWAS, we define the AlphaGenome score $\hat{S}_{vg}$ as the one with the largest absolute value across tracks corresponding to GTEx tissues.

Given that the true causal variant within a GWAS credible set is also usually unknown, we applied several strategies to derive a single, representative AlphaGenome score (SCG) per credible set (C) and its putative causal gene (G):

- *PIP-weighted average:* A credible set provides a discrete probability distribution over multiple candidate variants, assuming it contains a single causal variant. We can estimate the expected value of the score for the causal variant (and a given gene), by integrating over the uncertainty about which variant is causal using the law of total expectation:

$$\hat{S}_{CG} = \mathbb{E}[\hat{S}_{CG} \mid G = g] = \sum_{v} \mathbb{E}[\hat{S}_{CG} \mid C = v, G = g] P(C = v) = \sum_{v} \hat{S}_{vg} P(C = v)$$

  We can estimate $P(C = v)$ by using the PIP for each variant in a 99% credible set, normalized to sum to one.
- *PIP-max:* We use the AlphaGenome score corresponding to the variant with the largest PIP in a credible set, thus assuming that the largest PIP is the causal variant.
- *Any-variant max effect:* We use the variant with the largest absolute AlphaGenome score in the credible set. This approach assumes that variants with large AlphaGenome scores are more likely to be the causal variant for the GWAS trait.

This analysis relies on the following simplifying assumptions:

- The putative causal gene identified by Open Targets' predictive model is correct.
- The (unknown) GWAS causal variant exerts its phenotypic effect primarily through changes in the expression of this putative causal gene, detectable within one of the GTEx tissues or cell types for which AlphaGenome provides predictions.

3. Calibrating to a specific sign accuracy Many credible sets may have an AlphaGenome score that is non-zero. However, some of these may be close to zero, and not be predicting the sign reliably. We can use known expression-modulating variants (eQTLs) to pick a score threshold, T_k, that yields a particular accuracy (e.g. $k = 80\%$) for predicting the correct sign. We can then ask how many GWAS credible sets (and their corresponding putative causal genes) have an absolute score greater than a threshold, T_k. To determine T_k we used the same set of fine-mapped eQTLs as in the 'eQTL Sign' evaluation, where we have AlphaGenome scores and strong evidence (PIP>0.9) that the variants are causal (for gene expression) in a given gene, as well as their observed direction of effect, with one change to account for the uncertainty in the causal tissue in the GWAS setting. That is, for a given variant/gene pair in the evaluation dataset, we define the ground truth sign as the sign of the tissue with the largest maximum absolute observed effect size, but we use the sign of the tissue with the largest maximum AlphaGenome score across *all* GTEx tissues in order to calculate accuracy. In **Fig. 4h** the calibrated score thresholds used for 80% (90%) accuracy are $T_{80} = 0.015$ ($T_{90} = 0.107$).

4. Identification of eQTL-Colocalizing GWAS Loci We identified GWAS credible sets likely sharing a causal variant with an eQTL by utilizing Open Targets' colocalization data (*variant_disease_coloc* table). Credible sets demonstrating a COLOC[27] posterior probability of a shared causal variant (H4) greater than 0.95 were classified as colocalizing. Out of the 18,537 curated GWAS credible sets, 3,132 (17%) satisfied this colocalization criterion.

5. Stratification of GWAS Loci for Analysis To explore how sign prediction coverage by AlphaGenome and eQTL colocalization varied with the characteristics of GWAS signals, credible sets were stratified based on the following criteria:

- *Single-variant credible sets:* These are sets that contain only one variant, thereby having little to no ambiguity regarding the identity of the potential causal variant.
- *Minor Allele Frequency (MAF):* Credible sets were categorized by the PIP-weighted average MAF of their constituent variants. Sets falling into the lowest quintile of these weighted average MAFs were labeled 'small MAF,' and those in the highest quintile were labeled 'large MAF.' MAF values for Non-Finnish Europeans were used, as supplied in the Open Targets' *variants* table (originally sourced from gnomAD).
- *GWAS effect size*: A similar quintile-based categorization was applied using the PIP-weighted average of the absolute value of each variant's estimated GWAS effect size (beta coefficient, from the *tag_beta* column in the Open Targets credible set table), defining 'small effect size' and 'large effect size' subsets.
- *High Causal Gene Probability:* Credible sets where the putative causal gene – as assigned by Open Targets' predictive causal gene model (detailed in their *locus2gene* table) – had an estimated probability (`y_proba_full_model` column) greater than 0.8.

Polyadenylation variants

We evaluate the model's ability to predict the effects of polyadenylation-altering variants using the tissue-pooled paQTL variant set from Borzoi, which contains fine-mapped 3' QTLs from the eQTL Catalog[96]. For all paQTL metrics, we used variants only in the 'test' set chromosomes (see 'Chromosome splits for variant benchmarks' above). We follow Borzoi paQTL processing steps to generate a dataset of causal paQTLs (n=613) that affect mRNA isoform abundance by altering polyadenylation (pA) site usage, together with a negative set (n=1950) controlled for distance to the nearest 3' cleavage site and for similar expression levels. We applied Borzoi's approach to calculating AUPRC by performing 100 permutations of randomly matching each positive SNP to one of multiple potential negative SNP matches and calculating the AUPRC. We then average AUPRC performance across all permutations to get our final metric. SNPs that are not scored because they do not have > 80% of PAS within the input interval or only have one PAS site are imputed as 0 scores for metric calculations.

Following Borzoi's methodology, we score paQTLs by comparing predicted RNA-seq coverage between the reference and alternative alleles at 3' cleavage sites i.e. polyadenylation signals (PASs). The scoring approach quantifies the log fold change in expression between the set of proximal PASs vs. the set of distal PASs, where the sets of proximal and distal PASs are the PASs upstream or downstream a given 3' cleavage site respectively. For each gene within the input window, and for both REF and ALT predictions:

1. Each PASs is extended with 400 bp of upstream context.
2. Predicted RNA-seq coverage is summed spatially to produce a single aggregated value per PASs per RNA-seq track.
3. For each possible split of PASs into proximal and distal sets, we compute the absolute log fold change: $|\log(\text{ALT}/\text{REF})|$.
4. The maximum value over all possible splits is taken for each track, producing one score per track.
5. The average score across tracks is then used as the final score for metric calculations.

Chromatin accessibility variants & bQTLs

We sourced the following chromatin accessibility variants - DNase-seq quantitative trait loci (dsQTLs) and chromatin accessibility QTL (caQTL) - evaluations from ChromBPNet paper.

We evaluate the models on their ability to distinguish causal versus non-causal variants, and the effect size of causal variants, on the following datasets:

- *dsQTL Yoruba*. Yoruba African ancestry dsQTLs from 70 lymphoblastoid cell-lines (LCLs).
- *caQTL European.* European ancestry caQTLs from ATAC-seq profiling in ~100 LCLs.
- *caQTL African.* Representing African ancestry of six ancestry subgroups caQTLs from ATAC-seq profiling in ~100 LCLs.

As per ChromBPNet, we further evaluate the models on their ability to predict the reported effect size of significant caQTLs in 2 specific cell types *microglia* and *coronary smooth muscle cells (SMCs)* by indexing into closest matching tracks. We also evaluate the model on their ability to predict binding QTL (bQTL) variants for the SPI1 pioneer TF identified from 60 Yoruban LCLs by indexing into SPI1 ChIP-seq tracks for Borzoi and AlphaGenome.

Although ChromBPNet trained specialized cell type-specific models for these tissues, we evaluate AlphaGenome and Borzoi zero-shot on these tasks by indexing into the closest matching tracks. As with all variant evals, we assign a VALID/TEST chromosome split and only report the TEST numbers.

- *Cell type matching for GM12878.* For the Yoruba dsQTL, European caQTL, and African caQTL tasks (causality and effect size prediction), these variants are from LCLs. The ChromBPNet model was trained on a single reference LCL (GM12878). We zero-shot extract AlphaGenome GM12878 predictions. AlphaGenome contains GM12878 predictions for both ATAC and DNase outputs, and we find that DNase GM12878 predictions perform slightly better. For Borzoi, GM12878 was only available for DNase, there are 2 tracks and we take the mean of these. We use the same recommended scorer for both (center mask, width=501, aggregation DIFF_LOG2_SUM which is LFC).
- *Cell type matching for microglia*. On the validation set, we examined which AlphaGenome tracks performed best for predicting microglia caQTL effect sizes and found that it was Microglia ATAC for Borzoi (kai182) and suppressor macrophage DNase (CL:0000862) for AlphaGenome (microglia are a type of macrophage). We then used these columns for predictions on the test set.
- *Cell type matching for coronary smooth muscle cells*. Similarly, we found that AlphaGenome's "Left cardiac atrium" (UBERON:0002079) ATAC track and Borzoi's "Vascular Smooth Muscle 2" (kai222) ATAC track performed best on the validation set.
- *Cell type matching for SPI1 bQTL tasks.* For AlphaGenome, we use ChIP-seq predictions for the SPI1 TF in GM12878 (EFO:0002784). Borzoi did not have SPI1 GM12878 ChIP-seq, the closest match was SPI1 GM12891.

Prioritization of Trait-Associated GWAS Variants

To assess AlphaGenome's ability to prioritize functional variants from GWAS, we first curated a benchmark dataset using credible set data from Open Targets[108] (sourced from `bigquery-public-data.open_targets_genetics`, release 22_09). This initial dataset contains variants statistically associated with a wide range of diseases and complex traits such as height and BMI. We applied stringent filtering to retain only high-confidence credible sets, requiring that at least one variant within each set demonstrated genome-wide significance for both marginal and conditional associations (marginal 'tag_pval' $P < 5 \times 10^{-8}$ and conditional 'tag_pval_cond' $P < 5 \times 10^{-8}$), and that the sum of Posterior Inclusion Probabilities (PIPs) for variants within any given set did not exceed 1.

To focus on potentially non-coding regulatory variants, we then excluded variants whose most severe consequence, as annotated by the Ensembl Variant Effect Predictor (VEP)[107], fell into protein-altering categories. The excluded categories comprised: 'missense_variant', 'frameshift_variant', 'stop_gained',

‘inframe_deletion’, ‘inframe_insertion’, ‘start_lost’, ‘stop_lost’, ‘coding_sequence_variant’, and ‘protein_altering_variant’. Following this, variants were deduplicated by selecting their maximum PIP value across all associated phenotypes. For the benchmark, variants with a final PIP ≥ 0.9 were labeled as ‘causal’ (positive examples), while those with PIP < 0.01 were labeled as ‘non-causal’ (negative examples). This dataset was subsequently rebalanced at the variant ID level to achieve an equal 50/50 ratio of causal to non-causal variants.

Given that GWAS variants can influence phenotypes through diverse molecular mechanisms affecting multiple genomic features, we trained a Random Forest (RF) classifier to integrate information from model-derived variant scores across modalities. This allowed us to evaluate the collective power of these scores in distinguishing causal from non-causal GWAS variants. The RF models were trained and evaluated using a 5-fold cross-validation scheme on the curated GWAS variant dataset, with average auROC performance reported. Input features were as follows:

- For AlphaGenome, the features input to the classifier consisted of a comprehensive set of variant scores derived from all its output modalities, using scorer configurations previously optimized on modality-specific evaluations (see Variant Scoring section).
- For Borzoi, features were generated using its published gene-agnostic variant scoring strategy[2], which involves calculating the L2 norm of log1p-transformed differences over a 524,288 bp window across all its track outputs.

In all cases we used the absolute values of scores for this task. The Random Forest classifier was implemented using the `RandomForestClassifier` function from Scikit-learn (version 1.0.2)[102], with the `max_depth` parameter set to 5 and other hyperparameters kept at their default values without further optimization. Note that this benchmark does not use the chromosome split described previously, as we did not use it to optimize our variant scorers.

The features used for the ‘baseline’ analysis were derived from publicly-available sources. We used the same set of features as listed in *eQTL functional segmentation*, except for those related to a specific gene (last to bullets).

TraitGym

To train and evaluate models using a random forest on the Traitgym complex variant benchmark, we followed the same approach as above.

To evaluate the ability of sequence-to-function models to predict trait-affecting variants (mendelian and complex) in a zero-shot fashion, we followed Benegas et al[55]. Specifically, for each track predicted by a model, we first computed the predicted log-fold change in activity per position (or bin) due to the variant and then calculated the L2 norm across the sequence. For simplicity, we excluded tracks which were not easily amenable to this score, either due to their sparsity (splice-site location and usage) or because they involved an additional dimension (contact maps and splice junctions). As a result, Borzoi and AlphaGenome have access to the same modalities in this evaluation. As in Benegas et al., we averaged effect predictions from the forward and reverse strand sequence for all models (see ‘Test-time augmentation’).

To aggregate variant scores across modalities, Benegas et al. applied another L2 norm along the track dimension. However, the models we analyzed differ in the number of tracks they predict, how these tracks are scaled and at which resolution they are predicted. Therefore, a single aggregation strategy may not perform well for all tested models. To account for this possibility, we evaluated each model under three different aggregations (L2, mean and max) and took the respective best. We found that AlphaGenome always performed best when taking the max across tracks. For mendelian traits, the max across tracks also performed best for Enformer and the Borzoi ensemble, but not for Borzoi *fold-0*, which

exhibited a slight boost from using the L2. For complex traits, both Borzoi and Enformer performed best under the L2 norm. Note that this benchmark does not use the chromosome split described previously, as we did not use it to optimize our variant scorers.

Enhancer-gene linking

Our evaluations are based on the dataset of CRISPRi-validated enhancer-gene pairs assembled in the ENCODE-rE2G study[12]. We followed the authors' approach of filtering for genes present in their annotations, resulting in 471 positives and 10356 pairs in total. We needed to also drop 3 additional element-gene pairs whose gene ids were not present in our annotation file (GENCODE v46), resulting in a final dataset of 471 positives and 10353 examples in total.

Zero-shot evaluation Each element-gene pair was scored using an established input gradient scoring method, derived from K562 RNA-seq data as previously described [2], to directly assess sequence importance. That is, we centered the reference input sequence on the target gene midpoint and computed gradients with respect to the input. The scalar used for gradient computation was the average predicted gene expression (log sum across exons) across K562 tracks. We then computed each element-gene pair score by taking a weighted average of absolute input gradient contribution scores in the local window centered at the element. A Gaussian kernel was used to compute the weighted average, with window size 2400 and standard deviation 300. Element-gene pair scores were normalized by dividing by the mean absolute input gradient across the input sequence, to account for genes being expressed at different levels. Putative enhancers falling outside a model's input context window (i.e. too far from the target gene) were imputed with zero scores.

The above strategy was applied to AlphaGenome and Borzoi-ensemble models. We benchmarked the models by comparing the area under the Precision-Recall curve for selected TSS distance bins, borrowing the ones reported in the ENCODE-rE2G paper. We used the TSS annotations provided by ENCODE-rE2G for binning the elements. As simple baselines, we have included random predictions and the inverse distance to TSS.

Complementing this, we also scored enhancer-gene pairs using our variant scorers by permuting the 2kb interval centered on the enhancer element 10 times and taking the mean effect on a specified track as the score **Extended Data Fig. 7a**.

Supervised evaluation The ENCODE-rE2G models[12] are logistic regression models trained on bundles of features, and evaluated on out-of-fold predictions. For ENCODE-rE2G and ENCODE-rE2G extended, we used the precomputed cross-validated scores on the K562 cell line provided by the authors. We evaluated the effect of including AlphaGenome-based features in their feature bundles by including the K562 RNA-seq input x gradient score from AlphaGenome as a single additional feature into the full ENCODE-rE2G extended feature set (**Extended Data Fig. 7b**) and re-running their full training pipeline (forked from their repository). We also evaluated the performance of this input x gradient feature alone, and in combination with the TSS distance feature.

Subsequently, we used a more comprehensive set of AlphaGenome variant scorer-based features from K562 including Allele-Specific Activity Scores (AAS) and differential variant effect scores for RNA-seq of the target gene, ChIP-seq for EP300 and H3K27ac, CAGE, Pro-cap, as well as H1-ESC contact maps (K562 contact maps were not in our model outputs). These variant scores were combined with the existing ENCODE features for training the supervised regression model (**Extended Data Fig. 7c**).

Benchmarking on the CAGI5 MPRA challenge

AlphaGenome's performance variant effect prediction was further evaluated on the experimental massively parallel reporter assays (MPRA) data from the CAGI5 challenge. All predictions were made using the native genomic sequence surrounding each variant, using hg19 (human) reference genome and GTF V19 annotations to extract input sequences to match vcf file coordinates. Variant effect scores were computed following either using our recommended variant scorers or the Enformer or Borzoi strategies as described below. Zero-shot performance was computed by taking the Pearson r correlation between DNASE variant effect scores averaged across cell type-matched DNase tracks and observed CAGI5 effects. Following our previous work, for the lasso regression we scaled test set features using scaling factors from the training set such that the training features had a mean and standard deviation of 0 and 1 respectively. We then applied sklearn.LassoCV to train a model for each locus using the corresponding CAGI5 challenge training set with 10-fold cross validation and n_alphas = 100. Enformer Lasso comparisons were cell type-agnostic, using the full set of cell type tracks for CAGE and DNase. Borzoi comparisons used either DNase alone, or jointly with ChIP-Histone and RNA output types, in combination with either a cell type-agnostic or cell type-matched strategies described below.

For comparisons against Enformer, we used the previously defined variant scoring strategy of using the predicted difference in coverage between the reference and alternative allele summed across a 512 bp mask centered on the variant (CAGE and DNase modalities). All CAGE features had a 1 pseudocount added and then were log transformed before computing this difference. For cell type-matched comparisons, we used the same gene name to substring mapping as defined by the paper with the following modifications for when there was no available match. (1) The additional inclusion of 'melanocyte' for *IRF4*, (2) The addition of 'adrenal gland' for *TERT* performed in GBM cells.

For Borzoi comparisons, we followed their strategy of using the log-fold changes of total coverage summed across a 4000 bp window centered on the variant (DNase, CHIP-HISTONE) or from the log fold changes of summed exon coverage of the target gene (RNA-SEQ). For cell type-matched comparisons, we used the same gene name to substring mapping as defined in their report with the following modifications. (1) The additional inclusion of 'melanocyte' for IRF4, (2) the substitution of the substring 'adrenal gland' for the kidney tubule cell ontology CURIE 'CL:1000507' for the loci *LDLR, HNF4A, MSMB, TERT, MYC* for subsetting DNASE and CHIP-HISTONE predictions. RNA predictions were still subsetted using 'adrenal gland' for these loci. See **Supplementary Table 10** for complete list of ontology CURIEs extracted from AlphaGenome predictions.

For average comparisons described in **Fig. 5j**, the following loci are excluded to ensure a fair comparison: TERT-GBM (TERT variants tested in GBM cells) because ChromBPNet only reports TERT-HEK293T variant performance, and MYC due to non-convergence of LASSO regression for both AlphaGenome and Borzoi. F9 is excluded from comparisons of LASSO regressions using only DNASE features due to non-convergence when using cell type-matched DNase features for both AlphaGenome and Borzoi. The F9 Pearson r performances using cell type-agnostic DNase features for AlphaGenome and Borzoi were 0.62 and 0.54 respectively. When cell type-matched comparisons are specified, AlphaGenome used the modified Borzoi cell type-matching strategy as described above.

Multimodal variant example: TAL1

We gathered clinically observed variants from published literature reporting T-ALL noncoding variants around *TAL1* (**Supplementary Table 11**): a cluster of 5' neo-enhancer mutations upstream of the *TAL1* TSS [6,33]; an intronic single nucleotide variant [34]; and a 3' neo-enhancer [35].

To predict the impact of mutations on TAL1 gene expression and local epigenetic changes, we used AlphaGenome's variant scorers. Variant effects of cancer-associated mutations were compared against background variants (length-matched randomly shuffled insertions at the loci). Background variants were either saturating (all possible combinations at that length), otherwise 100 shuffles were used. Variant

scores in CD34+ common myeloid progenitors from TAL1 RNA-seq, DNAase, histone modifications such as H3K4me1/3, H3K27ac, H3K27me3, H3K9me3, H3K36me3) were collected. There is no tissue matched contact map, therefore we reported the mean effect across all contact map tracks.

To visualize a heatmap of these predicted effects, variant effects for each track were min-max scaled, grouped by their insertion length and position (as grouped in **Fig. 6c**).

Model Ablations

The following sections detail the specific experimental setups for the ablation studies presented in **Fig. 7**. Unless otherwise specified, models were trained using the same architecture, pre-training data, optimization procedures and evaluation metrics as described throughout the Methods section. All ablation experiments were conducted using four independent training runs with different random seeds, unless stated otherwise.

The performance across these studies was assessed on human tracks only and by using a consistent set of metrics: RNA-Seq Pearson correlation (log1p transformed counts), RNA-Seq gene-level Pearson correlation (Log Fold Change), Junction Counts Ratio Pearson correlation, ATAC-seq Pearson correlation, Histone ChIP-seq Pearson correlation, Contact Maps Pearson correlation, eQTL Sign prediction auROC, eQTL Causality prediction auROC (gene-balanced dataset), sQTL Causality prediction auPRC, Splicing Outlier prediction auPRC, Chromatin Accessibility Variants Causality auPRC (average of dsQTL Yoruba, European caQTL, and African caQTL tasks), and paQTL prediction auPRC.

To ensure a fair comparison across all model configurations, we implemented a specialized evaluation protocol for all ablation results. While in our primary analyses use fixed target intervals (defined by Borzoi, see Dataset splitting and cross-validation), here we uniformly tiled the validation set genomic regions with non-overlapping target intervals. For track prediction, the size of these intervals was dynamically set to 3/16 of the input sequence length. This formula maintains the same proportional evaluation window across all tested sequence lengths and corresponds to the standard 196kb for 1 Mb input. For contact map evaluations we tiled the validation by the full input sequence length. Contact map evaluations were also performed at AlphaGenome's native 2048 bp resolution, without interpolation, and averaged over all 28 human datasets.

Impact of Target Resolution In this study, we systematically varied the output resolution for our natively high-resolution targets: gene expression (RNA-Seq, CAGE-seq, PRO-cap), DNA accessibility (ATAC-seq, DNase-seq), and splicing tracks. Separate models were trained where these tracks were predicted at 1 bp, 2 bp, 8 bp, 32 bp, and 128 bp. The model's inherently lower-resolution targets – ChIP-seq and contact maps – were held constant and trained at their native resolutions (128 bp and 2048 bp, respectively) in all experiments. This design allowed us to isolate the effect of changing the resolution of one set of tasks on the performance across the entire model.

For these ablations, the core model architecture, including the decoder structure up to the point of embedding extraction, remained unchanged. The decoder embeddings are extracted at the resolution of the target tracks, and used to compute the output embeddings with the `output_embedder` module described in Output Heads. The specific methods used to downsample the high-resolution ground truth data were as follows:

- For continuous tracks (RNA-Seq, ATAC-seq, etc.), 1 bp resolution counts were summed into consecutive bins after augmentation was applied at base resolution.
- For per-base classification (splice sites), 1 bp resolution ground truth labels (positive classes) were max-pooled to the target resolution.
- For splice junction count prediction, which relies on base-resolution positions of donor and acceptor sites, lower resolution embeddings from the Output Embedder were upsampled by repeating along

the sequence axis (e.g., 4 times for 4 bp embedding), and used by the junction prediction head as if at base-resolution.

Impact of Sequence Length during Training and Inference This ablation explored the effect of varying input DNA sequence length (8kb, 32kb, 131kb, 512kb, and 1 Mb) on model performance. AlphaGenome's architecture is designed to be trained or evaluated at any sequence length that is a multiple of 2048 (the resolution of the pair activations modules), and the training and evaluation sequence lengths can differ. Three scenarios were tested: Three scenarios were tested:

- *Fixed Training Length, Variable Evaluation Length (Blue Series):* A single set of models, pre-trained using 1 Mb input sequences, was evaluated using varying input sequence lengths at inference time.
- *Variable Training Length, Fixed Evaluation Length (Purple Series):* Models were trained using input sequences of varying lengths. During training, the batch size was adjusted inversely to the input sequence length to maintain a constant cumulative sequence length processed per gradient step across runs. All models in this series were subsequently evaluated using a fixed 1 Mb input sequence length during inference.
- *Matched Training and Evaluation Length (Green Series):* Models were trained and evaluated using the same matched input sequence length. In all evaluation scenarios, we uniformly tile the holdout set by the model's target interval (the specific sub-sequence of model predictions that we evaluate) to ensure a consistent and unbiased comparison across different sequence lengths.

Impact of Ensembling and Distillation To assess the benefits of ensembling and distillation, 64 independent models were pre-trained using the Fold 0 data partition. Single student models (with the same architecture) were produced by distilling knowledge from ensembles of these 64 Fold 0 pre-trained teacher models, using 1, 4, or 64 unique teachers in the distillation process, following the procedure described in the main "Distillation" methods. The performance of mean ensembles was evaluated by averaging the predictions of randomly selected subsets of these 64 pre-trained models, with ensemble sizes ranging from 1 to 4.

Impact of Multimodal Learning (Modality Ablations) To investigate the contribution of different data types to learning shared representations and to overall predictive performance, models were trained with gradients restricted to specific modality groups. For each ablated model, the standard loss functions were applied normally to all prediction heads. However, for prediction heads outside this target group, stop-gradients were applied between the model embeddings and the head linear layer for both mouse and human tracks within each modality group. This ensured that these other heads were still trained (allowing their accuracy to be evaluated) but did not contribute to the learning of the shared model trunk representations.

The performance of these modality-specific models (n=8 seeds per group) was compared against the fully trained multimodal model (n=4 seeds). The modality groups investigated were: accessibility (ATAC-seq, DNase-seq, and chromatin contact maps), expression (RNA-Seq, CAGE-seq, and PRO-cap), splicing (splice site classification, splice site usage, and splice junction counts), and Histone ChIP-seq.

Cumulative modality addition experiments To determine the marginal performance contribution of each data modality, we trained a series of cumulative models. This ablation study started with a baseline model trained only on RNA-seq data. Subsequent models were trained by progressively adding data modalities in a fixed order, allowing us to quantify the performance gain at each step. The cumulative order of addition was: 1) RNA-seq, 2) + ATAC-seq, 3) + DNase-seq, 4) + Histone Modifications, 5) + TF ChIP-seq, 6) + Contact Maps, and 7) + CAGE/PRO-cap (the full model).

Model performance analysis and visualization

Data analysis used Python v.3.11.8 (https://www.python.org/), NumPy v2.2.5 (https://github.com/numpy/numpy), SciPy v.1.14.1 (https://www.scipy.org/), seaborn v.0.12.2 (https://github.com/mwaskom/seaborn), Matplotlib v.3.9.1 (https://github.com/matplotlib/matplotlib), Pandas v.2.2.3 (https://github.com/pandas-dev/pandas), anndata v0.11.4 (https://github.com/scverse/anndata), Scikit-learn v1.6.1, (https://github.com/scikit-learn/scikit-learn), InterpretML v0.6.10 (https://github.com/interpretml/interpret), and Colab (https://research.google.com/colaboratory).

Supplementary Figures

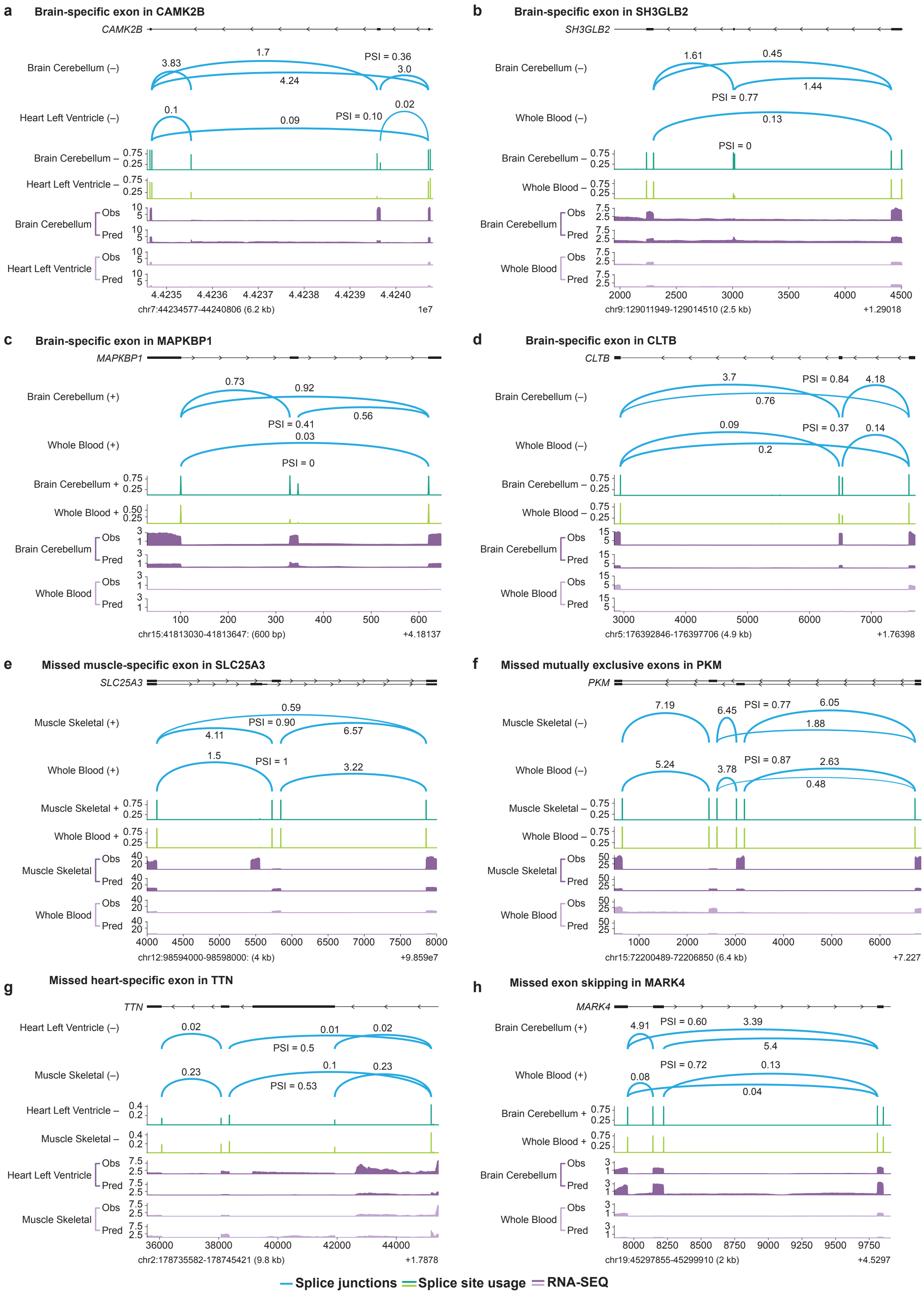

Supplementary Figure 1 | **Examples of AlphaGenome predictions for tissue-specific alternative splicing.**

Supplementary Figure 1 | *(continued)*
(a) Panels illustrate both successful predictions and specific challenges/failure modes. Coordinates are hg38. AlphaGenome generally predicts differential splice site usage and splice junction patterns across tissues, with failures typically involving missed predictions of specific tissue-regulated exon inclusion or skipping events. Measured data from GTEx. Predicted and observed RNA-seq, predicted splice site usage, and splice junctions surrounding an alternative exon (chr7:44239589-44239663) in CAMK2B, exhibiting differential splicing between neuronal and non-neuronal tissues[49]. **(b-d)** Additional examples of predicted splicing patterns around alternative exons exhibiting neuronal vs non-neuronal splicing[49] in: **(b)** SH3GLB2 (chr9:129013008-129013019), **(c)** MAPKBP1 (chr15:41813330-41813347), and **(d)** CLTB (chr5:176396479-176396532). **(e)** Failure case: Predictions surrounding an alternatively spliced exon in SLC25A3. AlphaGenome fails to predict the observed inclusion of the muscle-specific exon. **(f)** Failure case: Predictions surrounding mutually exclusive exons in PKM. AlphaGenome erroneously predicts the inclusion of both exons in Muscle and Blood tissues where only one is expected. **(g)** Failure case: Predictions surrounding exons 49 and 50 of TTN. AlphaGenome fails to predict the observed inclusion of the heart-specific exon 49. **(h)** Failure case: Predictions surrounding an alternative exon (chr19:45298143-45298222) in MARK4. AlphaGenome fails to predict the observed exon skipping event in Blood relative to neuronal tissues[49].

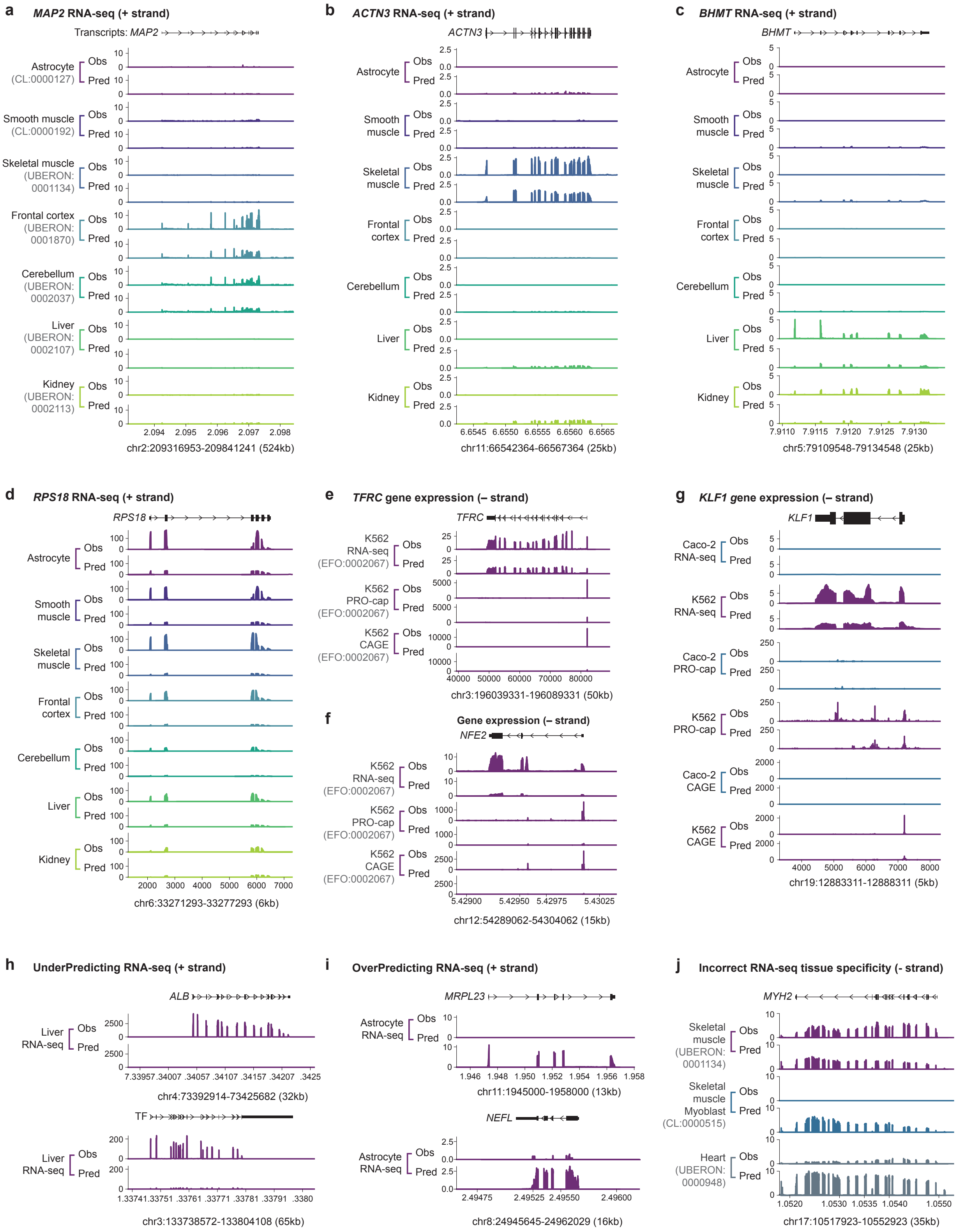

Supplementary Figure 2 | **Examples of AlphaGenome gene expression predictions.** Observed (obs) versus predicted (pred) signals for various expression-related assays across different genomic loci (hg38 coordinates) and tissues/cell types, illustrating both successful predictions and limitations. AlphaGenome generally captures expression patterns across multiple expression assays (RNA-seq, CAGE, PRO-cap), with failures typically involving underprediction of signal magnitude and errors in tissue specificities.

Supplementary Figure 2 | *(continued)*
(a) Accurate prediction of *MAP2* (Microtubule Associated Protein 2) expression in brain tissues (frontal cortex, cerebellum) but absence in astrocytes. **(b)** Accurate prediction of *ACTN3* (Actinin Alpha 3) expression specifically in skeletal muscle tissue. **(c)** Accurate prediction of *BHMT* (Betaine-Homocysteine S-Methyltransferase) expression in liver and kidney tissues. **(d)** Prediction for the housekeeping gene *RPS18* (Ribosomal Protein S18). The RNA-seq expression patterns are largely correct, but signal magnitude is underestimated across tissues. **(e)** Example of multi-output type expression analysis (RNA-seq, CAGE, PRO-cap shown) for *TFRC* (Transferrin Receptor) in K562 cells. Predicted signal output shapes are accurate but magnitudes are underestimated. **(f)** Additional multi-output type expression analysis example for *NFE2* (Nuclear Factor, Erythroid 2) in K562 cells. **(g)** Multi-output and multi-tissue expression analysis for *KLF1* (KLF Transcription Factor 1), correctly predicting high expression in K562 but absence in Caco-2 cells. **(h)** Failure cases: Underprediction of RNA-seq signal for liver-specific genes *ALB* (Albumin) and *TF* (Transferrin). **(i)** Failure cases: Overprediction of *MRPL23* (Mitochondrial Ribosomal Protein L23) and *NEFL* (Neurofilament Light Chain) RNA-seq signal in astrocytes. **(j)** Failure case: Incorrect tissue-specificity predicted for *MYH2* (Myosin Heavy Chain 2), showing slight underprediction in skeletal muscle alongside overprediction in skeletal muscle myoblasts and heart tissue.

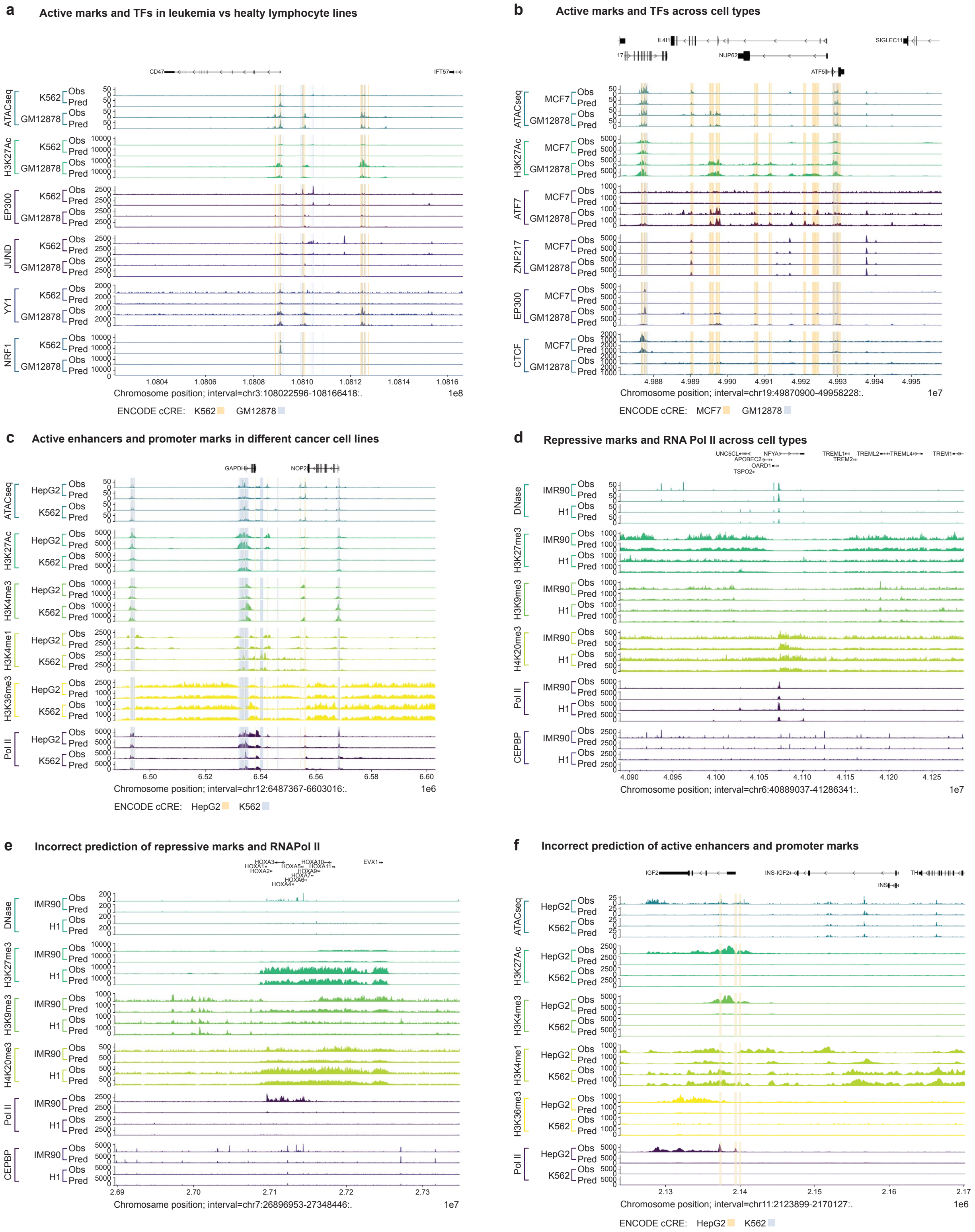

Supplementary Figure 3 | **AlphaGenome prediction examples for chromatin tracks.** Comparison of predicted (pred) and observed (obs) epigenetic features and transcription factor (TF) binding profiles on held-out genomic locations (hg38), illustrating capabilities and limitations. AlphaGenome is broadly accurate at predicting diverse chromatin landscapes, including accessibility, histone modifications, and protein binding, often capturing cell-type specific patterns seen across lineages. Observed prediction failures typically involve underpredicting expected signal magnitude or presence in specific contexts, rather than placing marks in incorrect locations.

Supplementary Figure 3 | *(continued)*
(a) Differential regulatory landscape at the *CD47* locus between K562 (leukemia) and GM12878 (lymphoblastoid) cell lines. Despite shared lymphoid origin and *CD47* expression, AlphaGenome correctly captures cell-line-specific differences in TF binding, EP300 DNA-binding cofactor occupancy, and histone modifications in the proximal regulatory region. **(b)** Cell-type-specific predictions at the *IL4I1* (Interleukin 4 Induced 1) locus. AlphaGenome accurately recapitulates chromatin accessibility and TF binding in GM12878 versus MCF7 (breast cancer) cells. **(c)** Active transcription marks at the ubiquitously expressed housekeeping gene *GAPDH* (Glyceraldehyde-3-Phosphate Dehydrogenase). AlphaGenome correctly predicts patterns for both broad and sharp active chromatin marks, as well as RNA Polymerase II binding. **(d)** Prediction of an active *NFYA* (Nuclear Transcription Factor Y Subunit Alpha) promoter region embedded within heterochromatin. AlphaGenome accurately predicts the overall chromatin landscape, including broad flanking repressive marks. **(e)** Failure case: Developmentally regulated *HoxA* (Homeobox A) cluster. AlphaGenome correctly predicts the repressed state in H1 human embryonic stem cells but fails to capture the activation of the anterior part of the cluster in IMR90 fibroblasts. **(f)** Failure case: Liver-specific gene regulation at the *IGF2* (Insulin Like Growth Factor 2) locus. While correctly predicting inactivity in non-expressing K562 cells, AlphaGenome fails to predict expression-associated histone modifications and RNA Polymerase II binding in expressing HepG2 liver cancer cells.

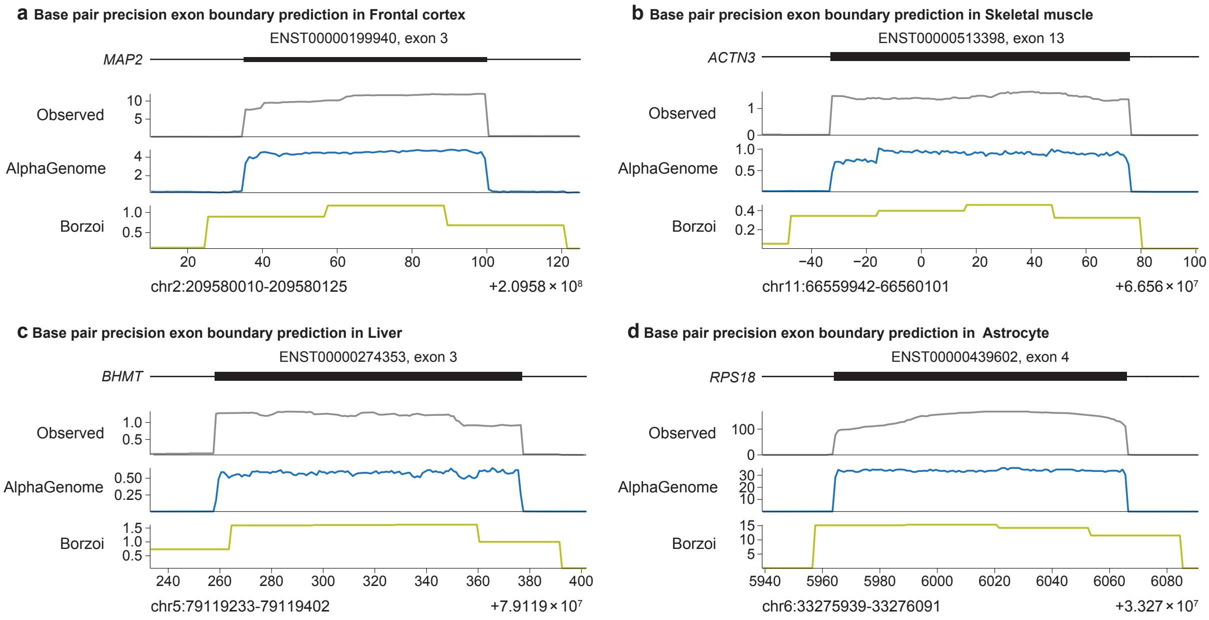

Supplementary Figure 4 | **AlphaGenome enables single base-pair predictions of RNA-seq coverage at exon-boundaries. (a)** Observed and predicted RNA-seq coverage for exon 3 of the *MAP2* gene in frontal cortex tissue (same gene as in S2.2a). AlphaGenome correctly predicts the exon boundaries. Borzoi predicts RNA-seq coverage in 32 bp bins, preventing exact inference of the beginning and end of the exon. **(b)** Observed and predicted RNA-seq coverage for exon 13 of the *ACTN3* gene in skeletal muscle tissue (c.f. S2.2b). **(c)** Observed and predicted RNA-seq coverage for exon 3 of the *BHMT* gene in liver tissue (c.f. S2.2c). **(d)** Observed and predicted RNA-seq coverage for exon 4 of the *RPS18* gene in astrocytes (c.f. S2.2d).

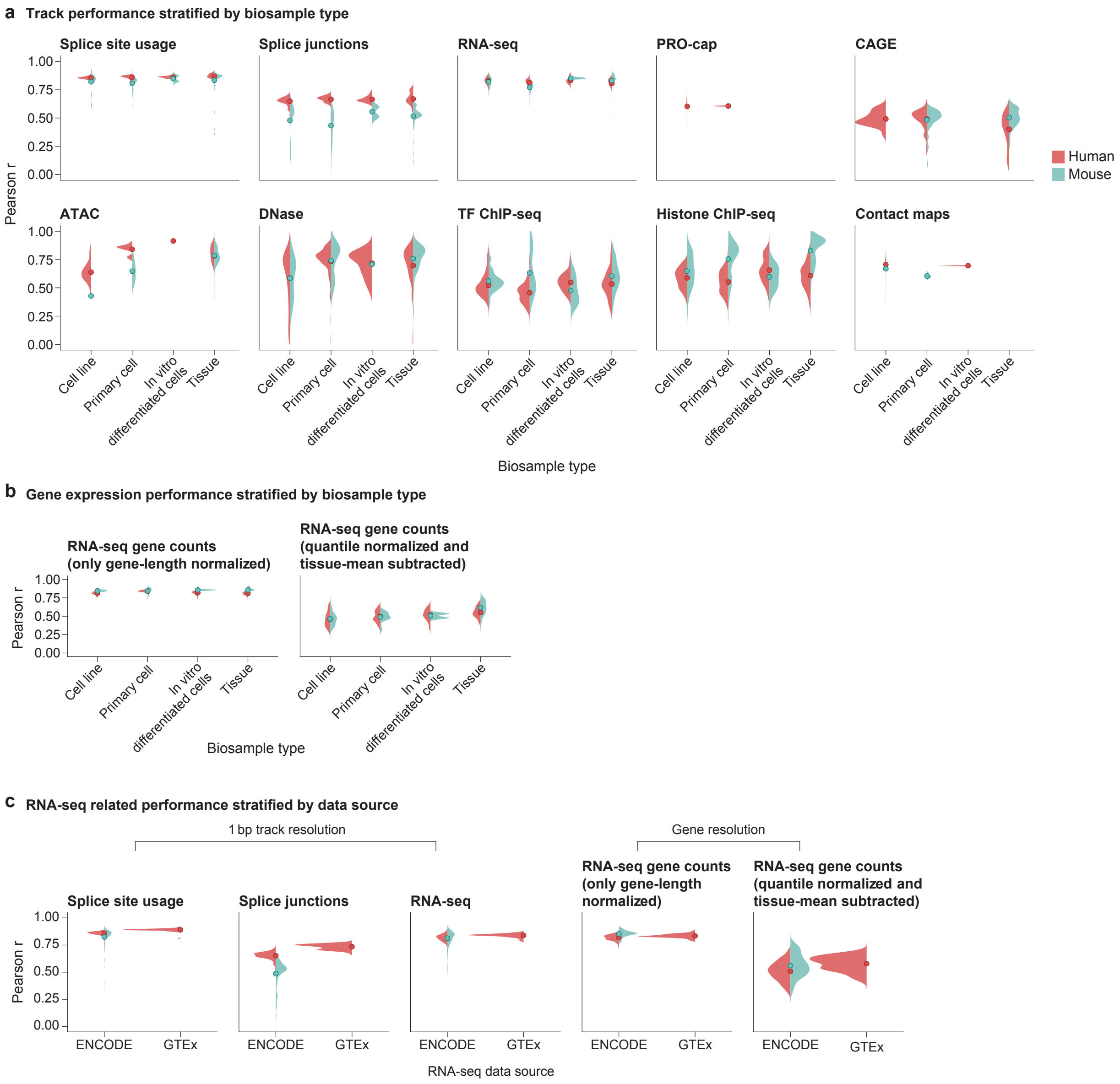

Supplementary Figure 5 | **Stratification of model performance supplementary to main Fig. 2c,d**. Model performance evaluation metrics stratified by metadata variables. **(a)** Track correlation performance (Pearson r, related to **Fig. 2c**) stratified by biosample type (cell line, primary cell, in vitro differentiated cells, tissue) for Human (red) and Mouse (blue). Performance is broadly consistent across types for most assays, with notable exceptions including lower performance observed for splice junctions in mouse primary cells, and for DNase-seq and ATAC-seq in cell lines compared to tissues. **(b)** Gene expression correlation performance (Pearson r, related to **Fig. 2d**) stratified by biosample type. Performance on both raw and normalized/mean-subtracted gene expression metrics remains generally consistent across different biosample types for both species. **(c)** Performance comparison for RNA-seq derived metrics (Pearson r, related to **Fig. 2c,d**) stratified by data source (ENCODE vs GTEx). Only minor performance differences are observed between the two data sources, such as slightly lower correlation for splice junctions derived from ENCODE data compared to GTEx.

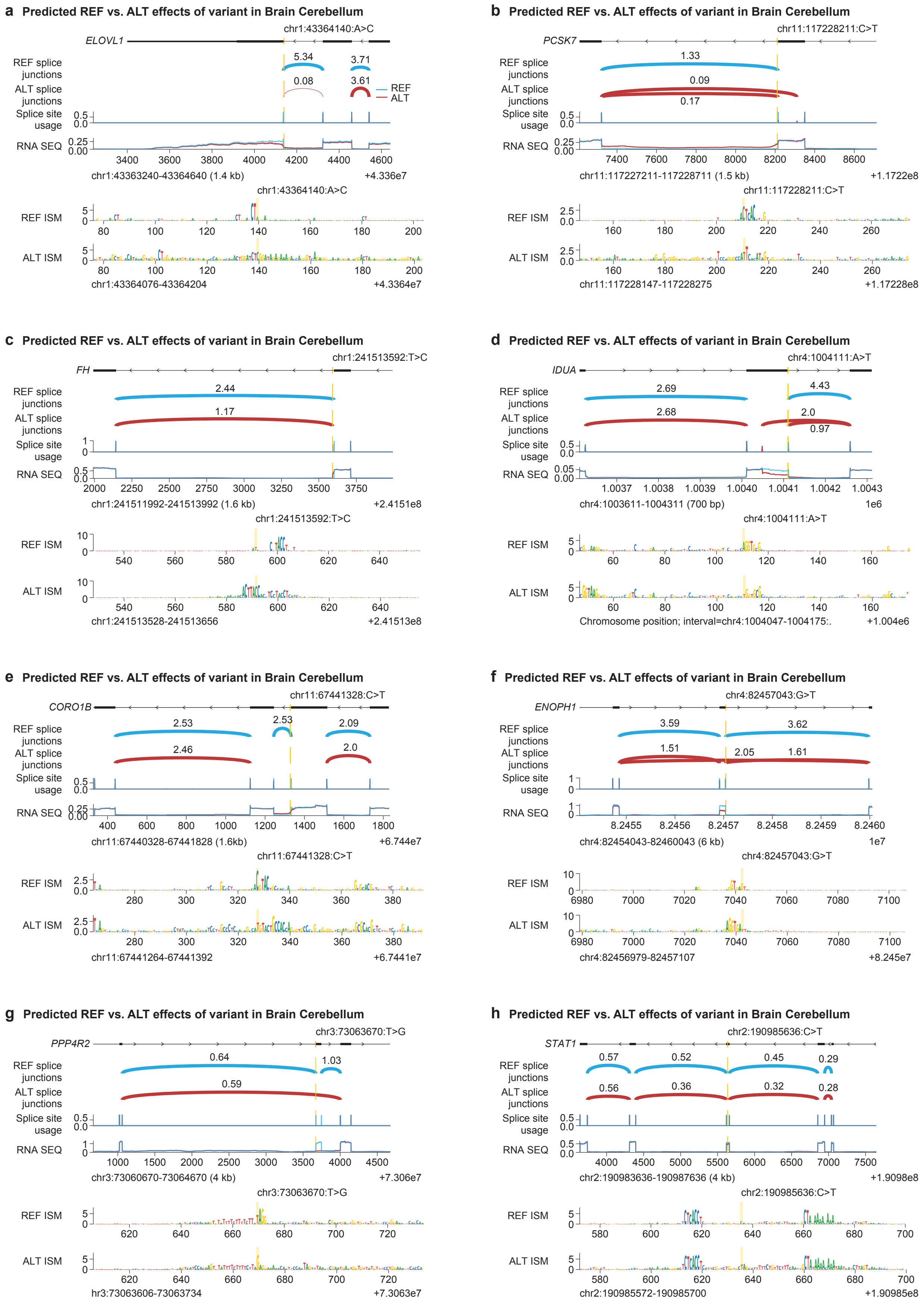

Supplementary Figure 6 | **AlphaGenome predictions on validated de novo cryptic splice mutations in autism spectrum disorder (ASD) individuals from the SpliceAI study[4].**

Supplementary Figure 6 | *(continued)*
(a-b) Reference and Alternate Splice Junction, Splice Site Usage and RNA-seq AlphaGenome predictions on cases with validated intron retention with the variant. ISM on both the Reference and Alternate sequences is also shown. **(c-e)** Example predictions where the variant results in the creation of novel junctions. In examples **(c)** and **(d)**, AlphaGenome clearly predicts the novel junctions, whereas in example **(e)** the model predicts an intron retention event. **(f-h)** Example predictions where the variant results in exon skipping. In examples **(f)** and **(g)**, AlphaGenome correctly predicts the exon skipping event, whereas in example **(h)** it misses it.

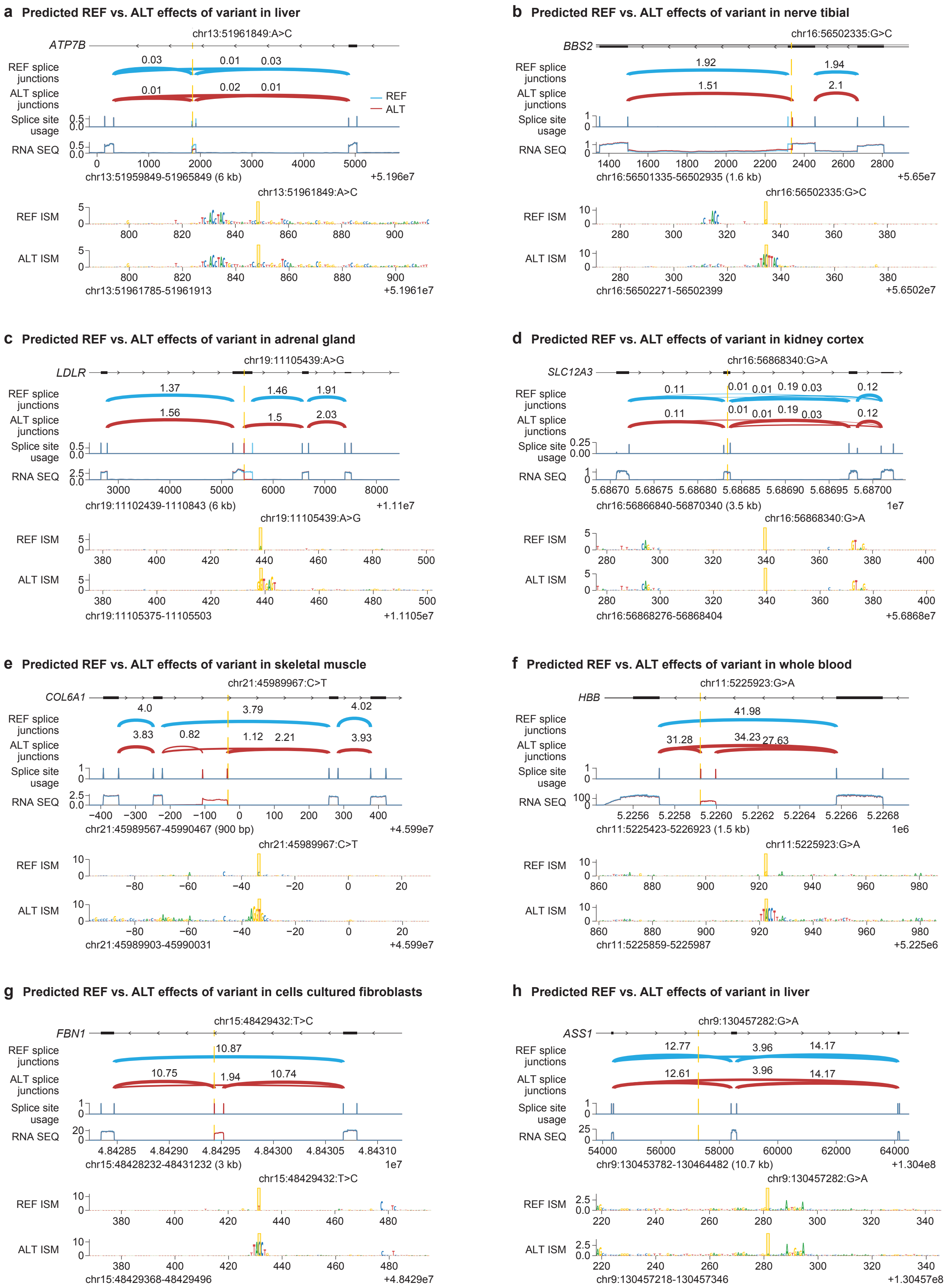

Supplementary Figure 7 | **AlphaGenome predictions for missense and deep intronic variant examples from ClinVar. Success and failure examples.**

Supplementary Figure 7 | *(continued)*
(a) Reference and Alternate Splice Junction, Splice Site Usage and RNA-seq AlphaGenome predictions on a missense variant associated with Wilson disease[109]. ISM on both the reference (REF) and alternate (ALT) sequences is also shown. AlphaGenome predicts an exon skipping event in the presence of the variant ($Ref\ PSI = 0.75$, $Alt\ PSI = 0.33$). **(b-c)** AlphaGenome predictions indicate the creation of novel junctions for two missense variants associated with (b) Bardet-Biedl syndrome[109,110] and **(c)** familial hypercholesterolemia. **(d)** AlphaGenome fails to predict a splicing effect for a missense variant associated with Gitelman syndrome[111]. **(e-g)** AlphaGenome predictions on ClinVar pathogenic deep intronic (distance to closest splice junction > 50 bp) variants. Predictions indicate exon inclusion events due to intronic variants associated with **(e)** Bethlem myopathy[112] and Ullrich congenital muscular dystrophy[113], **(f)** beta-thalassemia[114,115], and **(g)** Marfan syndrome[114]. **(h)** AlphaGenome fails to predict a splicing effect for a deep intronic variant associated with citrullinemia[116].

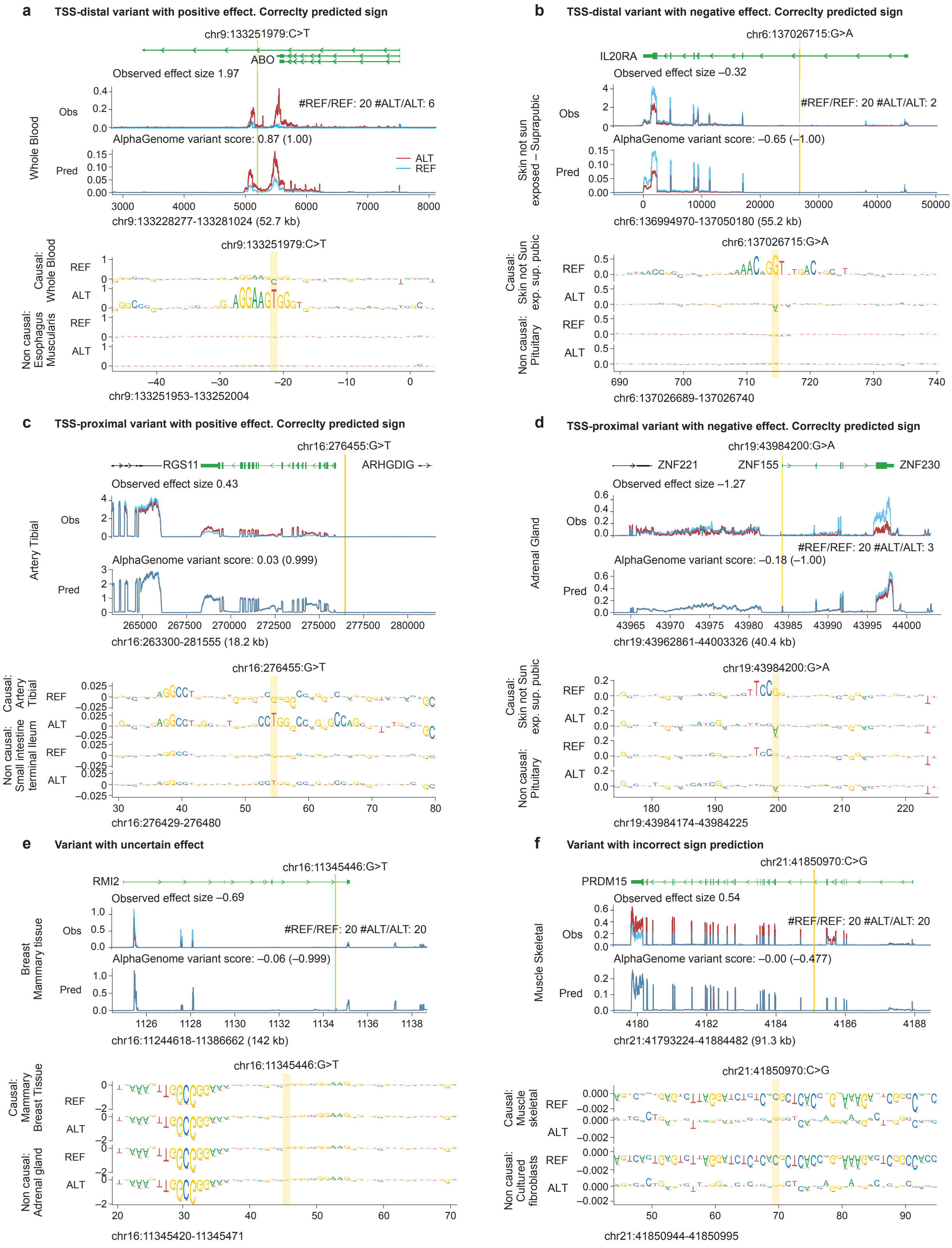

Supplementary Figure 8 | **AlphaGenome eQTL effect predictions.**

Supplementary Figure 8 | *(continued)*
(a) Example of distal variant (12-35kb from the target gene TSS) with a positive effect and correctly predicted sign. The variant generates a new ETS-like binding site resembling a known binding site (Jaspar matrix #MA0080.2) for SPI1, a critical transcription factor for myeloid and lymphoid cells development. ISM scores highlight how the motif is only relevant in the GTex tissue where this eQTL was identified, Whole Blood. **(b)** Example of distal variant with a negative effect and correctly predicted sign, with the variant leading to reduced gene expression. **(c)** Example of proximal variant with positive effect and correctly predicted sign. The variant generates a CCTGG sequence, a known non-CpG target for DNA methylation associated with gene repression. **(d)** Example of proximal variant with negative effect and correctly predicted sign. The variant disrupts the edge of an ETS1-like motif (Jaspar matrix #MA0098.1) leading to strongly reduced motif score and correctly predicted reduction in gene expression. **(e)** Example of an ambiguous case for a distal variant with correctly predicted sign. ISM scores do not highlight any relevant motif for either alleles overlapping the variant. Only a tissue non-specific motif is evident nearby. **(f)** Example of failure case on a variant with incorrectly predicted effect and very low ISM scores.

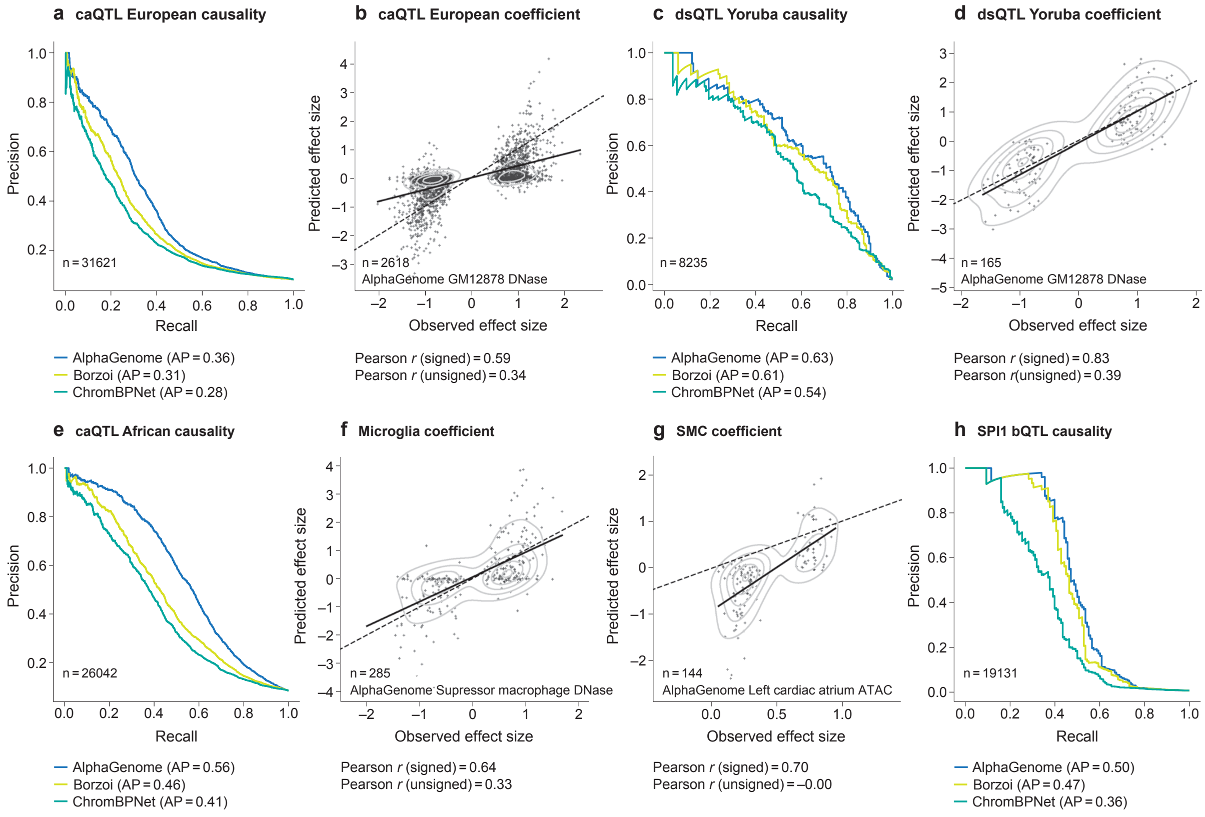

Supplementary Figure 9 | **Additional accessibility variant analysis.** Extended evaluation of variant effect prediction on chromatin accessibility across diverse contexts. AP = average precision (auPRC). Signed Pearson R correlation uses raw values; unsigned Pearson R uses absolute values first. **(a)** Precision-Recall curves comparing AlphaGenome, Borzoi, and ChromBPNet performance on caQTL causality prediction in European ancestry. **(b)** Scatterplot comparing AlphaGenome's predicted versus observed effect sizes (Coefficient) for causal caQTL variants in European ancestry. **(c)** Precision-Recall curves comparing AlphaGenome, Borzoi, and ChromBPNet performance on dsQTL causality prediction in Yoruba ancestry. **(d)** Scatterplot comparing AlphaGenome's predicted versus observed effect sizes (Coefficient) for causal dsQTL variants in Yoruba ancestry. **(e)** Precision-Recall curves comparing model performance for caQTL causality prediction (African ancestry). **(f)** Effect size prediction for microglia causal caQTL variants. Scatterplot compares observed effects versus AlphaGenome's predicted DNase effects in a closely-related available cell type (suppressor macrophage). **(g)** Effect size prediction for cardiac smooth muscle cell (SMC) causal caQTL variants. Scatterplot compares observed effects versus AlphaGenome's predicted ATAC effects in a closely-related available cell type (left cardiac atrium ATAC). **(h)** Precision-Recall curves comparing model performance for SPI1 bQTL causality prediction.

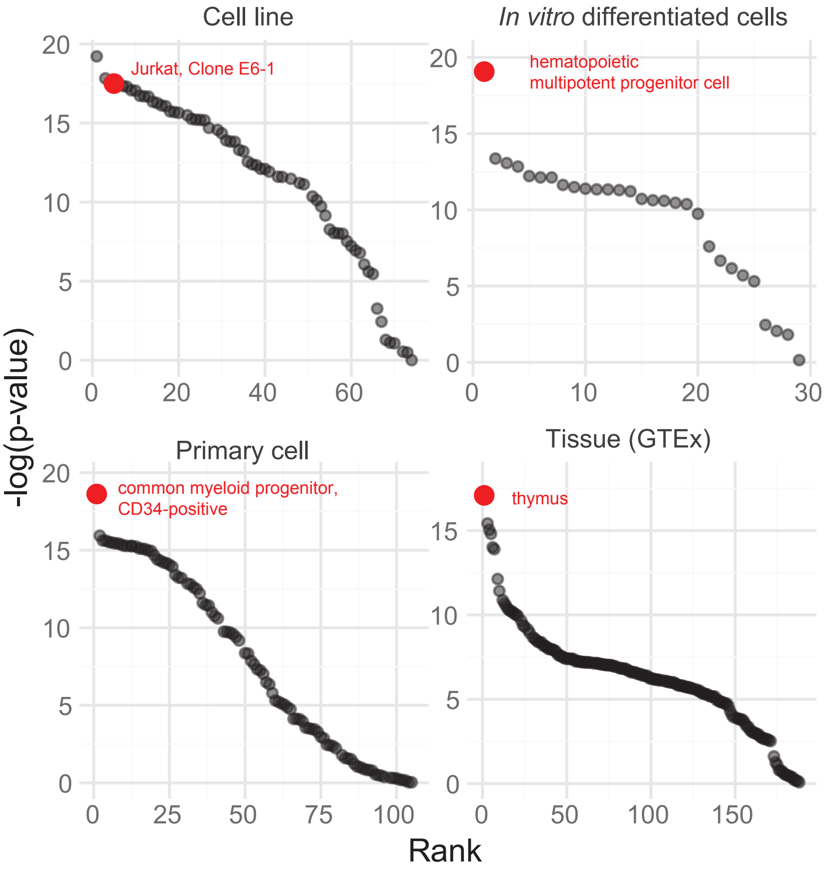

Supplementary Figure 10 | **Tissue specificity of *TAL1* upregulating oncogenic variants.** Each data point is a human RNA-seq track. For each track, the group difference between predicted *TAL1* expression for oncogenic and shuffled background variants defined in **Fig. 6** was assessed using a two-sided Mann-Whitney U statistic. The plot is split across four biosample types: *in vitro* differentiated cells, primary cells, GTEx tissues, and cell lines. Within each group, each sample is ranked in the x-axis by the magnitude of the Mann-Whitney U statistic (on the y-axis). A single labeled red data point highlights the sample that most closely matches the T-ALL tissue of origin within that biosample type.

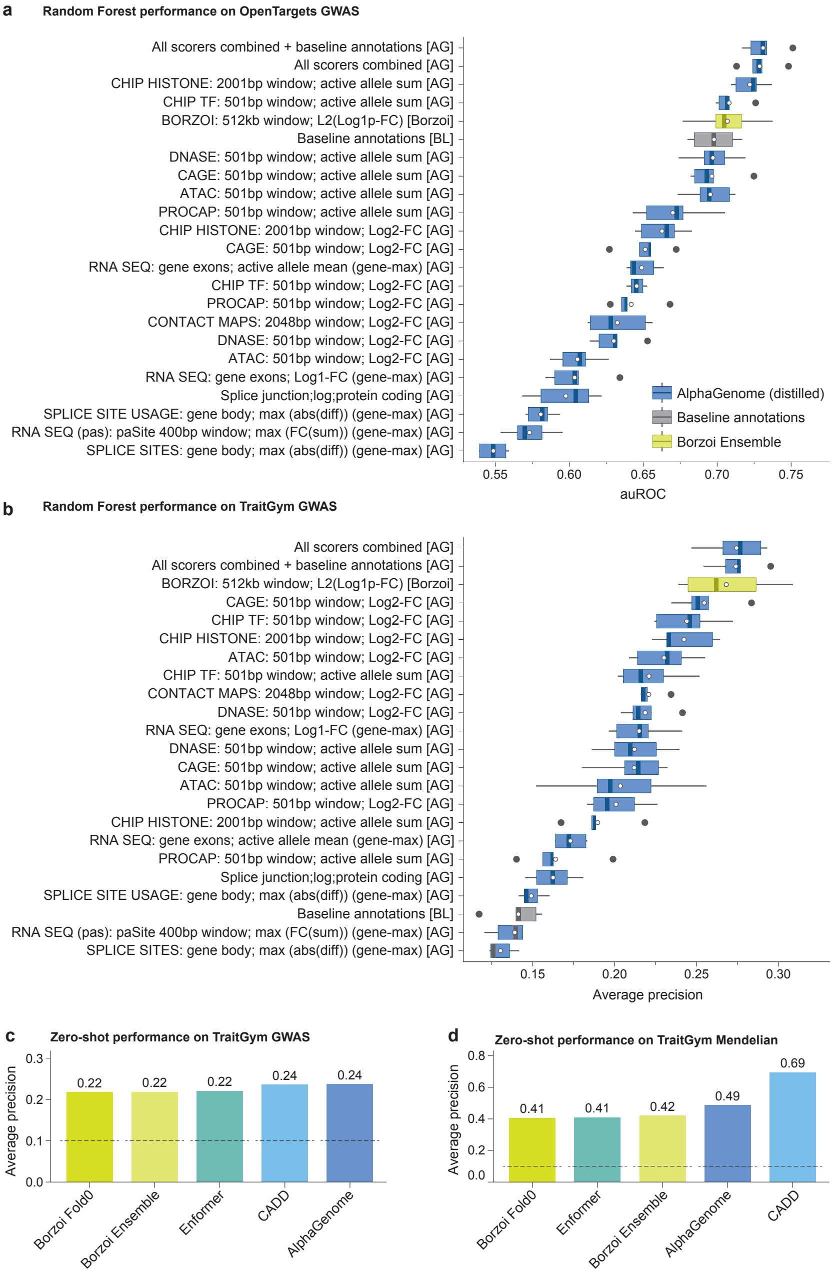

Supplementary Figure 11 | **Evaluating models on complex and mendelian traits. (a)** AUROC for classifying whether variants are implicated in complex traits using random forests trained on model predictions or baseline features ($n = 7636$). This evaluation uses positive and negative variants derived from the OpenTargets dataset[108] (Methods). Random forests were evaluated using five-fold cross-validation, white dot indicates the mean across folds. Whiskers represent 1.5 times the interquartile range. **(b)** Average Precision (auPRC) for classifying whether variants are implicated in complex traits using random forests trained on model predictions or baseline features ($n = 11400$). This evaluation uses causal and control variants as defined in Traitgym[55]. Random forests were evaluated using five-fold cross-validation, white dot indicates the mean across folds. Whiskers represent 1.5 times the interquartile range. **(c)** Average Precision (auPRC) for classifying whether variants are implicated in complex traits using aggregated zero-shot model predictions (Methods, $n = 11400$). This evaluation uses the same variants as in **(b)**. However, as no random forest is trained, no cross-validation is performed. Grey line indicates random performance (class balance). All methods, including CADD, exhibit moderate predictive power. **(d)** Average Precision (auPRC) for zero-shot classification of whether a variant is implicated in a mendelian trait or is a matched negative control, sampled from common variants ($n = 3380$). Causal and control variants are taken from TraitGym. AlphaGenome outperforms previous sequence-to-function models. However, CADD strongly outperforms all other methods, likely because it can leverage conservation information to separate rare from common variants. Grey line and error bars as in **(c)**.

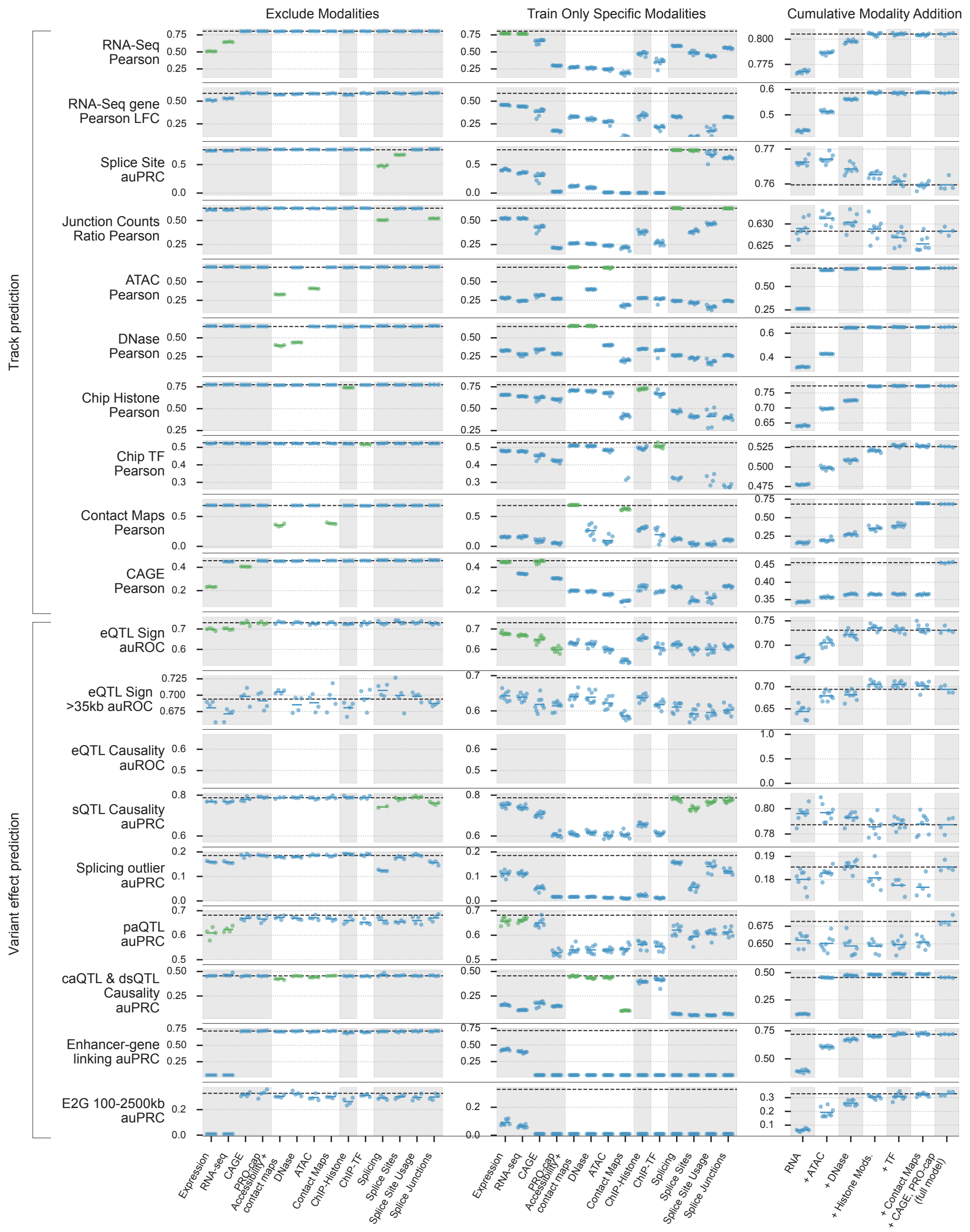

Supplementary Figure 12 | **Impact of excluding, isolating, and cumulatively adding modalities on track and variant tasks.** Comparison of AlphaGenome performance on variant and track prediction tasks when training excludes a single output/modality group (left), when training only a single modality group (middle), and when cumulatively adding modalities (right). The x-axis indicates the specific output head (e.g., 'CAGE'; regular size label) or the broader modality group (Expression, Accessibility + contact maps, and Splicing; bolded label) targeted by the ablation. Each dot represents an independent training run (n=4 for 'Exclude Modalities' and n=8 for 'Train only Specific Modalities' random seeds); black dashed lines indicate the mean performance of the full multi-task model for reference; green dots indicate when the tasks is related to the ablated modality and blue is the default color.

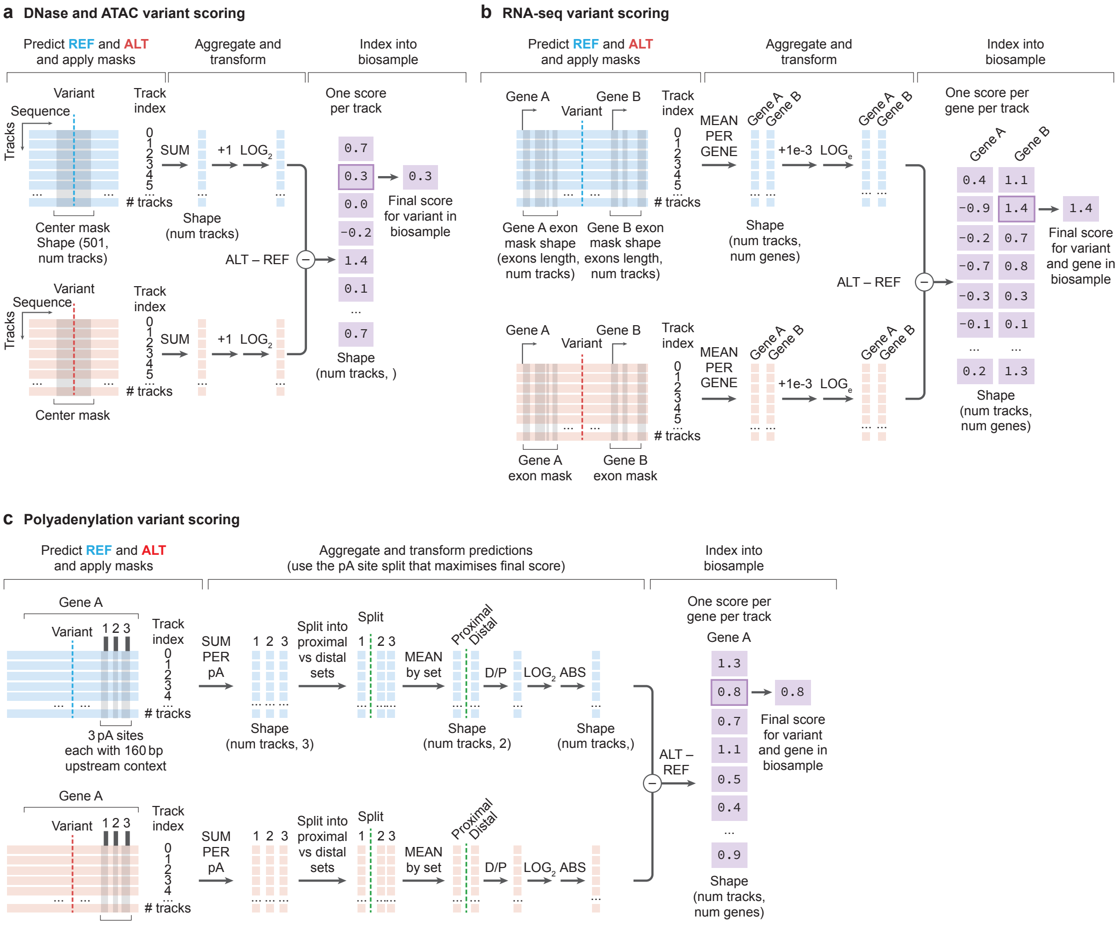

Supplementary Figure 13 | **Illustrative workflows for selected variant scoring mechanisms. (a) Chromatin accessibility variant scoring (i.e. DNase/ATAC)**. Schematic illustrating a center-mask based approach. Signals from reference (REF) and alternative (ALT) allele predictions are summed within a window, log2-transformed, and then subtracted to quantify variant impact on local chromatin accessibility. **(b) RNA-seq gene expression variant scoring**. Schematic for gene-based scoring of expression changes. REF and ALT predictions are averaged within gene boundaries, log-transformed, and then subtracted to estimate a log-fold change per gene. **(c) Polyadenylation variant scoring** Schematic for assessing variant effects on polyadenylation site usage. The workflow involves comparing REF and ALT predictions at potential polyA sites to determine changes in relative proximal versus distal site usage, summarized as a maximum log-fold change of isoform ratios.

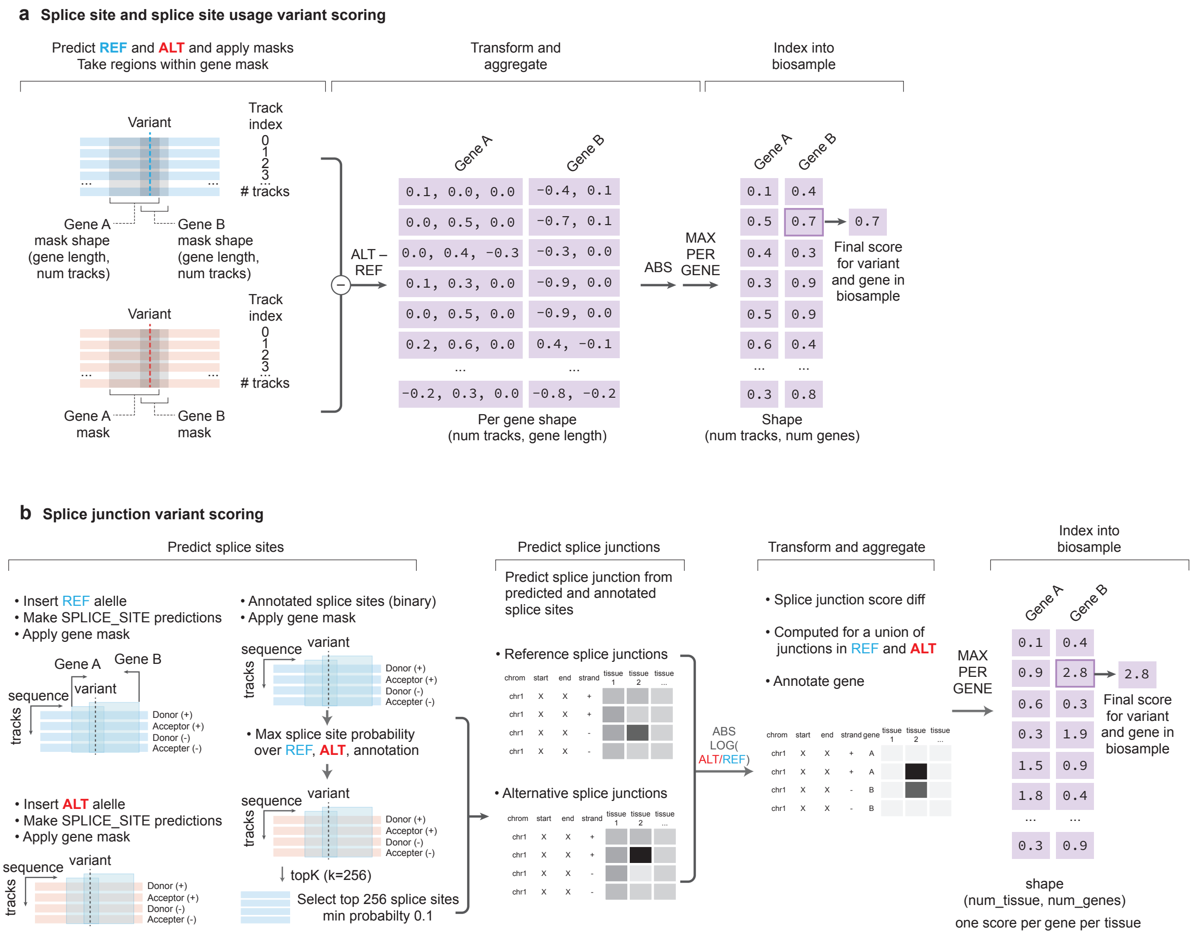

Supplementary Figure 14 | **Illustrative workflows for splicing variant scoring mechanisms. (a) Splice site and splice site usage variant scoring**. Schematic for gene-based splicing impact. Differences between REF and ALT splicing predictions are calculated within gene body regions, an absolute value is taken, and the maximum absolute difference across relevant sites forms the variant score. **(b) Splice junction variant scoring**. Schematic for gene-based splicing impact scored with the splice junction head. Only predicted and annotated splice sites from the genes overlapping the variant are considered for splice junction predictions. Absolute log fold change on splice junction scores is computed for all predicted junctions. A maximum score per tissue is used as the variant score for that tissue.

Supplementary Note: AlphaGenome shows competitive performance on the CAGI5 MPRA benchmark

Massively parallel reporter assays (MPRAs) provide high-throughput functional readouts, experimentally measuring the impact of sequence variation on gene regulation, albeit in artificial constructs lacking native chromatin context. Building upon previous work demonstrating strong performance by models like Enformer on the CAGI5 saturation mutagenesis MPRA challenge[31,32], we evaluated AlphaGenome on the same dataset, comparing against Enformer, Borzoi, and ChromBPNet (**Extended Data Fig. 8**). Using a zero-shot approach with cell-type matched scores derived from accessibility predictions alone (DNase), AlphaGenome performed comparably to both ChromBPNet and the Borzoi Ensemble (overall Pearson $r \approx$ 0.54-0.56; **Extended Data Fig. 8a**). Furthermore, employing a cell-type agnostic strategy and using LASSO regression to aggregate features derived from multiple AlphaGenome tracks (e.g., DNase combined with CAGE), AlphaGenome achieved strong correlations ($r \approx$0.61) comparing favorably to both Enformer ($r \approx$ 0.548) and Borzoi ($r \approx$ 0.549) across different feature sets (**Extended Data Fig. 8b**). We then explored the use of LASSO regression on other modalities. Using Borzoi's variant scoring strategy of ensembling over cell-type matched tracks from multiple modalities (DNASE and CHIP and RNA or only DNASE and CHIP, **Extended Data Fig. 8c**) improved both AlphaGenome ($r \approx$ 0.609 - 0.635) and Borzoi's ($r \approx$ 0.599 - 0.635) performances. Finally, we tried a cell type agnostic LASSO regression across these combined modalities using recommended scorers for each modality (**Extended Data Fig. 8d**) and saw AlphaGenome's highest performance ($r \approx$ 0.648 - 0.662) and (Borzoi $r \approx$ 0.584 - 0.601), indicating that specific cell type matching may not be necessary, and can hinder performance if they are not optimized properly. These results on an independent MPRA benchmark further validate AlphaGenome's ability to predict the effects of regulatory variants on gene expression, achieving performance competitive with state-of-the-art models.

Supplementary Note: Trait-altering variants

Linking genetic variants to their phenotypic consequences, particularly complex traits and diseases, remains a central challenge in genomics. This task is complicated by the difficulty in establishing gold-standard causal variants for benchmarking, often relying on fine-mapped GWAS signals or curated databases like OMIM. Such limitations frequently necessitate aggregating data across diverse traits, potentially introducing heterogeneity. Furthermore, the optimal strategy for selecting negative variants in these benchmarks is often unclear, yet it critically influences performance assessment. To evaluate AlphaGenome's utility in prioritizing trait-associated variants, we explored two benchmarking approaches. The first was an in-house benchmark using Open Targets data, where positives were fine-mapped GWAS variants (PIP >= 0.9) and negatives were sampled from low-PIP variants (PIP < 0.01) to achieve a 50:50 class balance. The second, TraitGym, also uses fine-mapped GWAS variants as positives but employs a negative set matched for baseline features like TSS distance, MAF, and linkage disequilibrium (LD), with a 9:1 negative-to-positive class balance. A key question is how to best leverage sequence-to-function models like AlphaGenome for this task, as they do not directly predict traits or pathogenicity. Intuitively, non-coding trait-associated variants should alter regulatory outcomes detectable by such models. However, the specific gene, tissue, and regulatory modality affected are often unknown, necessitating methods to aggregate multiple model predictions. We explored both training random forests for this aggregation and a simpler zero-shot approach (e.g., taking the maximum predicted effect). Our benchmarking revealed that the choice of negative set heavily influences apparent predictor performance. When negative variants were not matched for baseline features, these baseline features themselves showed predictive power, although AlphaGenome modestly outperformed them and Borzoi (Supplementary Fig. 11a). In this scenario, modalities detecting variant location within cis-regulatory elements were more predictive than actual variant effect scores. Conversely, when negatives were matched for baseline features (as in TraitGym), these features were, unsurprisingly, not predictive (Supplementary Fig. 11b). Here, variant effect scores became more informative, though the performance differences between models using either random forest aggregation or zero-shot approaches were not

substantial (Supplementary Fig. 11c). We found no evidence that explicitly adding baseline features improved AlphaGenome's predictions, suggesting it inherently captures such information. Overall, however, robustly solving the general variant-to-trait prioritization problem remains challenging. GWAS signals are often distally located, posing difficulties for current sequence-based models, and complex traits likely require integrating information beyond cis-regulatory effects, such as gene function and network interactions. Even perfect prediction of steady-state gene expression changes due to cis-regulatory variants would likely be insufficient for many traits. TraitGym also provides a benchmark for distinguishing Mendelian disease variants (typically rare and monogenic) from TSS-distance and consequence matched common variants. On this task, where mechanisms might be simpler and more directly tied to strong regulatory feature changes, sequence-to-function models, including AlphaGenome, generally performed better than on the GWAS benchmark (Supplementary Fig. 11d). AlphaGenome offered a slight improvement over previous sequence-based models. However, broad-spectrum predictors like CADD16, which leverage conservation, remain highly competitive, partly because conservation implicitly accounts for gene essentiality and function – factors that sequence-to-function models focusing purely on regulatory impact by design will not capture. We also note that in the TraitGym Mendelian benchmark, due to matching criteria, about 7% of negative control variants had a high probability (PIP > 0.5) of being eQTLs in at least one study in the eQTL catalogue, which sequence-to-function models might understandably rank highly. Overall, it will likely be necessary to integrate sequence-to-function models with other measures, such as conservation and estimates of gene function, to enable direct predictions of variant deleteriousness.

Supplementary Note: Revisiting clinically annotated non-coding variants with ACMG guidelines

This is a case-by-case breakdown of the performance of AlphaGenome on the set of non-coding variants provided in the Ellingford *et al.* 2022 ACMG guidelines for non-coding variants[117].

We obtained Table S1 from Ellingford *et al.* 2022, distributed in Figshare (CC BY 4.0 license). This table contains a set of genetic variants and their candidate disease genes from real-world anonymized disease cases, as well as a series of ACMG classifications (VUS, Likely Pathogenic/Pathogenic, Likely Benign/Benign) and evidence codes and curator notes for those ratings.

For each row of the table, we used the "Disease/phenotype" to match a tissue of origin to the AlphaGenome prediction metadata. We then performed basic analysis of the variant using the AlphaGenome API. For each variant, we analysed the effect of the variant on genome tracks available for the tissue of interest (`predict_variant`), scored the variant effect using the default variant scorers (`score_variant`), reporting both raw and quantiled scores when appropriate. For a subset of variants we used *in silico* mutagenesis (`score_ism_variants`) with AlphaGenome to examine contribution scores for the nucleotides including and surrounding the variant position to identify candidate motifs.

Based on these predictions, we assessed whether or not each variant has *in silico* evidence of variant effect. For variants whose clinical curation notes contained reference to experimental evidence, we assessed the extent to which the *in silico* evidence matched what was observed experimentally.

Summary of AlphaGenome applied to non-coding variant case studies.

Region type	Variant ID (Gene)	Prior Curation	AlphaGenome Finding	*In silico* evidence of variant effect	Impact
**Splicing**	chr1_215867824_G_C (*USH2A*)	VUS	Precisely predicts the creation of a 129 bp intronic cryptic exon, matching prior minigene assays.	Yes	Corroborates existing functional evidence
**Splicing**	chr15_42387805_C_G (*CAPN3*)	VUS	Predicts clear exonic skipping. SpliceAI at default settings misses this effect.	Yes	Corroborates existing functional evidence, see Case study 2
**Splicing**	chr11_66519596_A_T (*BBS1*)	Pathogenic	Predicts multiple competing novel splice acceptors, explaining the "complex splicing impact" noted by curators.	Yes	Corroborates existing functional evidence
**Splicing**	chr15_48430805_G_C (*FBN1*)	Likely Pathogenic	Accurately predicts a 2-nucleotide exon extension, replicating the exact molecular outcome seen in patient RNA-seq.	Yes	Corroborates existing functional evidence
**Splicing**	chr1_94010795_C_T (*ABCA4*)	Pathogenic	Predicts a drop in exon coverage and decreased splice site usage, consistent with curator notes on incorrect splicing.	Yes	Corroborates existing functional evidence
**Promoter**	chr2_127418408_A_G (*PROC*)	VUS	Predicts major loss of expression, disruption of activating histone marks, and identifies a promoter motif.	Yes	Corroborates existing functional evidence
**Promoter**	chr8_11703860_G_T (*GATA4*)	Not Available	Predicts no meaningful change in local activity or gene expression, suggesting the variant is non-functional.	None	Supports benign classification
**Promoter**	chr11_2171856_C_T (*TH*)	Likely Pathogenic	Predicts strong decrease in expression and confirms disruption of the known CREB motif via ISM.	Yes	Corroborates existing functional evidence
**Promoter**	chr13_48303720_T_G (*RB1*)	VUS	Predicts decreased expression and DNase accessibility; ISM pinpoints the key functional motif within a known CRE.	Yes	Corroborates existing functional evidence
**Promoter**	chrX_71223179_T_C (*GJB1*)	VUS	Predicts strong negative impact on expression; ISM identifies the critical SOX10 binding motif within an ENCODE CRE.	Yes	Extends existing evidence (base pair resolution motif within CRE)
**Enhancer**	chr1_209816135_A_AA (*IRF6*)	VUS	Predicts decreased expression and identifies a functional TF binding motif within an VISTA CRE.	Yes	Extends existing evidence (base pair resolution motif within CRE)

Continued on next page

Region type	Variant ID (Gene)	Prior Curation	AlphaGenome Finding	*In silico* evidence of variant effect	Impact
			Continued from previous page		
**Enhancer**	chr7_156791413_A_C (*SHH*)	Likely Pathogenic	Predicts decreased local enhancer marks but misses the effect on gene expression ˜1MB away.	Partial	None
**Enhancer**	chr11_31664397_C_A (*PAX6*)	Likely Pathogenic	Predicts decreased local DNase accessibility but misses the effect on gene expression ˜128kb away.	Partial	None
**Enhancer**	chr17_70680162_T_C (*SOX9*)	Benign	*Limitation:* Cannot predict; variant is 1.5Mb from the gene, exceeding the model's input length.	None	None
**3' UTR**	chr11_5225488_A_T (*HBB*)	VUS	Predicts strong expression decrease by disrupting a canonical polyadenylation motif.	Yes	Changes classification **VUS** $\rightarrow$ **Likely Pathogenic**, see Case study 1
**3' UTR**	chrX_49250456_T_C (*FOXP3*)	VUS	Predicts a clear drop in expression by identifying a disrupted polyA motif.	Yes	Corroborates existing functional evidence
**3' UTR**	chr1_25816825_T_C (*SEPN1*)	VUS	*Limitation:* Predicts weak effect; true mechanism is disruption of mRNA secondary structure (out of scope).	None	None
**3' UTR**	chr1_100195897_T_G (*DBT*)	Likely Pathogenic	Correctly predicts decreased expression but underestimates the magnitude.	None	None
**3' UTR**	chr14_75958692_G_A (*TGFB3*)	VUS	Predicts no significant changes to gene expression.	None	None
**5' UTR**	chr5_1295046_T_G (*TERT*)	VUS	Predicts upregulation, providing *in silico* evidence that clarifies prior contradictory functional data.	Yes	Resolves ambiguity, supports Pathogenic
**5' UTR**	chr1_21509427_C_T (*ALPL*)	VUS	Predicts inconsistent and weak effects on expression at the TSS.	None	None
**5' UTR**	chr8_22130633_C_T (*HR*)	VUS	*Limitation:* No transcriptional changes predicted; mechanism is a missense change in a uORF (out of scope).	None	None
**5' UTR**	chr10_27100453_T_C (*ANKRD26*)	VUS	Predicts slight upregulation but with low confidence and some discordance.	None	None
**5' UTR**	chr19_11089410_CT_C (*LDLR*)	VUS	Predictions for expression changes are inconsistent across different readouts.	None	None

Continued on next page

Region type	Variant ID (Gene)	Prior Curation	AlphaGenome Finding	*In silico* evidence of variant effect	Impact
Continued from previous page					
**RNA gene**	chr2_121530927_G_A (*RNU4ATAC*)	Likely Pathogenic	*Limitation:* No effect predicted; known mechanism (RNA secondary structure) is out of scope for the model.	None	None
**RNA gene**	chr3_169764963_CAG_C (*TERC*)	VUS	*Limitation:* No transcriptional effect predicted; known mechanism (catalytic activity) is a non-transcriptional outcome.	None	None
**RNA gene**	chr9_35657920_G_GCA (*RMRP*)	Pathogenic	*Limitation:* No effect predicted; mechanism is post-transcriptional (mRNA cleavage impairment).	None	None
**RNA gene**	chr3_169764918_C_T (*TERC*)	VUS	Predicts only a slight effect on the short transcript, with a quantile score that may be artificially high.	None	None
**miRNA**	chr7_129774757_C_T (*MIR96*)	VUS	*Limitation:* Fails to predict miRNA expression, a limitation of training on total/polyA+ RNA-seq data.	None	None
**miRNA**	chr15_79209844_C_T (*MIR184*)	Likely Pathogenic	*Limitation:* Fails to predict miRNA expression, a limitation of training on total/polyA+ RNA-seq data.	None	None

We highlight two case studies in this Supplemental Note. The remaining 28 case studies and all the code for generating the plots and analyses is available in a public Colaboratory notebook that can be run with an AlphaGenome API key.

Case study 1: HBB 3'UTR VUS (`chr11:5225488:A>T`) in Beta-thalassemia

Ellingford *et al.* curation:

- Classification: VUS
- Inheritance: Recessive
- CADD: 16.41
- Splice AI: 0
- Consensus ACMG rules: PM2_Supporting; PM1_Supporting; PP4; PM3
- Curator notes: "Disrupts polyadenlylation signal. Pathogenic variant at same position and multiple other ClinVar pathogenic variants across the PolyA signal. PM1_Supporting used, but could alternatively apply PM5. Found with a pathogenic variant, but in trans not confirmed, and also as a homozygote (PM3)."

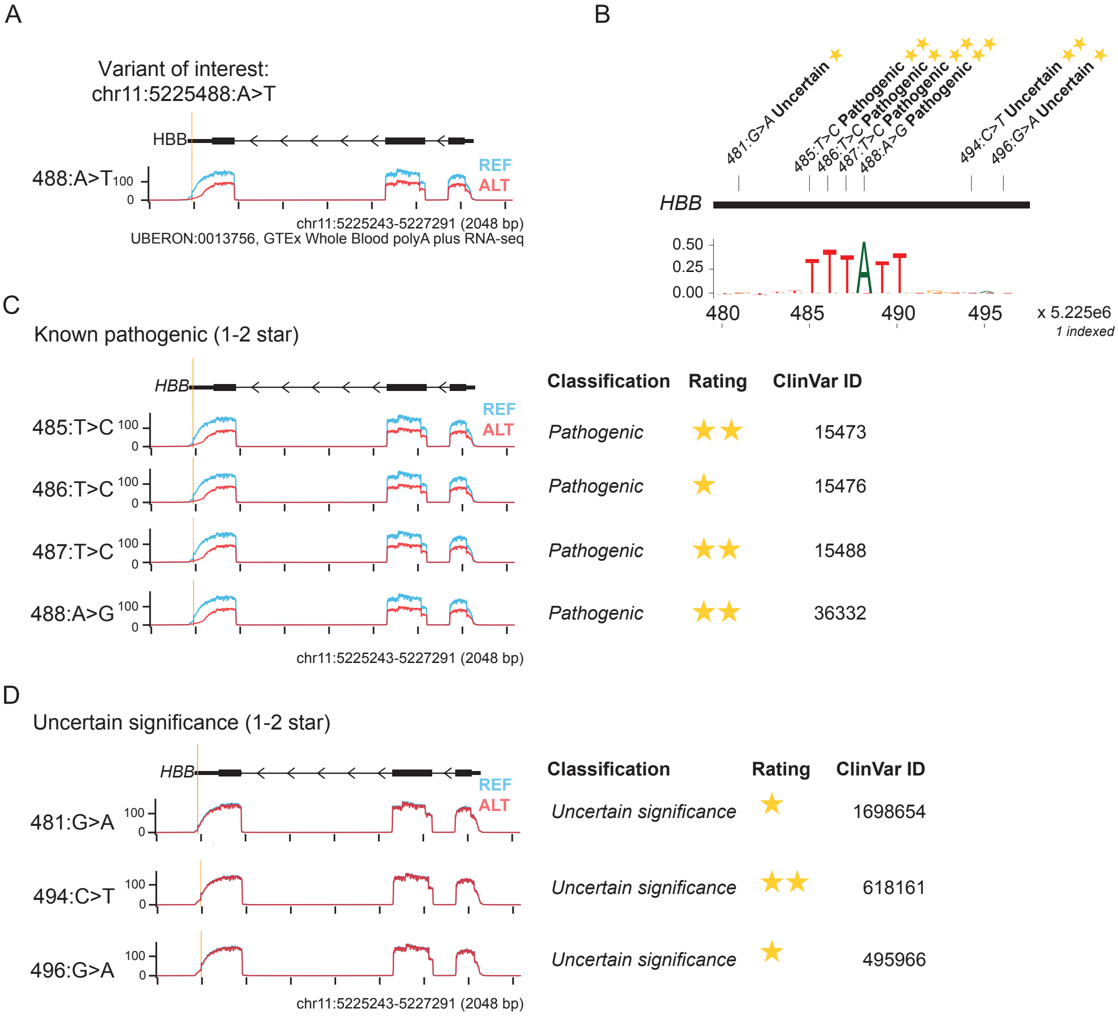

Supplementary Figure 15 | **AlphaGenome predictions for variant `chr11:5225488:A>T` and related variants in whole blood tissue. (a)** *HBB* RNA-seq track predictions for the variant of interest, `chr11:5225488:A>T`. Reference and alternate allele predictions are shown. **(b)** *In silico* mutagenesis of the positions surrounding `chr11:5225488:A>T`. Contribution scores (y-axis) indicate the contribution of each position to *HBB* expression. Nearby ClinVar variants are labeled. **(c)** *HBB* RNA-seq track predictions for the ClinVar pathogenic variants of interest from (b). **(d)** RNA-seq track predictions for the ClinVar variants of uncertain significance from B.

AlphaGenome interpretation:

We analyzed this variant's effect in whole blood (`UBERON:0013756`), the relevant tissue for Beta thalassemia. AlphaGenome predicted the variant would decrease *HBB* gene expression (Supplementary Fig. 15A). Further *in silico* mutagenesis revealed that the variant disrupts a canonical polyadenylation motif in the gene's 3' UTR (Supplementary Fig. 15B).

To benchmark this prediction, we analyzed known pathogenic variants in the same region. The original curators noted the presence of "other ClinVar pathogenic variants across the PolyA signal." We queried ClinVar (accessed October 1, 2025) for pathogenic single nucleotide variants ($\geq$1 star) within 20 bp of the VUS. This identified four pathogenic variants, all of which were located within the five nucleotides that AlphaGenome predicted were most critical for *HBB* expression (Supplementary Fig. 15B). Crucially, AlphaGenome's predictions for these known pathogenic variants mirrored those for the variant of interest, showing a similar decrease in predicted *HBB* expression (Supplementary Fig. 15C).

We identified three other nearby VUSs ($\geq$1 star) in ClinVar that were located outside the polyadenylation site. In contrast to the pathogenic variants, AlphaGenome predicted that these VUSs would have no

effect on HBB expression (Supplementary Fig. 15D). This aligned with their ClinVar curation notes, which mentioned that these variants have not been observed in beta-thalassemia cases.

Conclusion: *In silico* evidence of variant effect, VUS → Likely pathogenic

AlphaGenome predicts that the variant affects RNA transcript levels by interfering with a canonical polyadenylation site. Importantly, this predicted effect is the same as that of known Pathogenic variants in the same polyadenylation site.

If this *in silico* evidence was available at the time of the curation in 2022, the curators would have been able to activate the ACMG evidence code PM5 ("variants with exactly the same impact, on the same gene, but not at the same nucleotide") at a Moderate level of evidence, instead of the weaker PM1_Supporting. This would have combined with their existing annotations (PM2_Supporting; PP4; PM3) to resolve the VUS to Likely Pathogenic.

The existing ClinVar annotation for this variant is a zero-star entry from 2004 from the authors of a case study; depositing an ACMG compliant Likely Pathogenic submission would result in a one-star entry that supersedes this submission and updates this variant with modern evidence. We are planning to submit this variant annotation to ClinVar.

Case study 2: CAPN3 Exonic VUS (`chr15:42387805:C>G`) in Muscular Dystrophy

Ellingford *et al.* curation:

- Classification: VUS
- Inheritance: Recessive
- CADD: 25
- Splice AI: 0.02 (at default settings)
- Consensus ACMG rules: PM2_Supporting; PS3_Supporting
- Curator notes: "Abnormal splicing seen in minigene assay - creation of an exonic splice site. Reported twice in LOVD. PP3 is not applied as PS3 is used."

AlphaGenome interpretation:

We investigated this variant in skeletal muscle tissue (`UBERON:0001134`), the relevant context for muscular dystrophy. Although the variant is a missense variant located within an exon of the *CAPN3* gene, AlphaGenome predicted exon skipping (Supplementary Fig. 16). This conclusion was supported by multiple lines of evidence: a predicted decrease in RNA-seq coverage (raw score = -0.017, quantile score = -1.0), a shift in splice site usage (raw score = 0.18, quantile score = 1.0), and the formation of a novel junction connecting the flanking exons (raw score 1.47, quantile score = 1.0).

This prediction of a splicing defect initially appeared to conflict with the low SpliceAI score (0.02) reported by the Ellingford *et al.* curators. We hypothesized this was due to a possible use of pre-computed scores from the VEP plugin, which uses a default search window (`max_distance`) of 50 bp. As the nearest splice acceptor is 52 bp from the variant, the default analysis would fail to detect its effect. Indeed, re-analyzing the variant with an expanded 500 bp window using SpliceAI Lookup yielded a delta score of 0.22, supporting a splicing defect and resolving the discrepancy. This observation is consistent with the limitations of precomputed SpliceAI scores reported recently[118].

Finally, we considered the variant's coding effect. As a `T184R` missense mutation in *CAPN3*, it receives high pathogenicity scores from protein-specific predictors like AlphaMissense (0.928) and PrimateAI-3D (0.92). Given our splicing evidence and the curators' reference to a supportive minigene assay, this variant possibly has a dual mechanism, impacting the protein both at the RNA (splicing) and

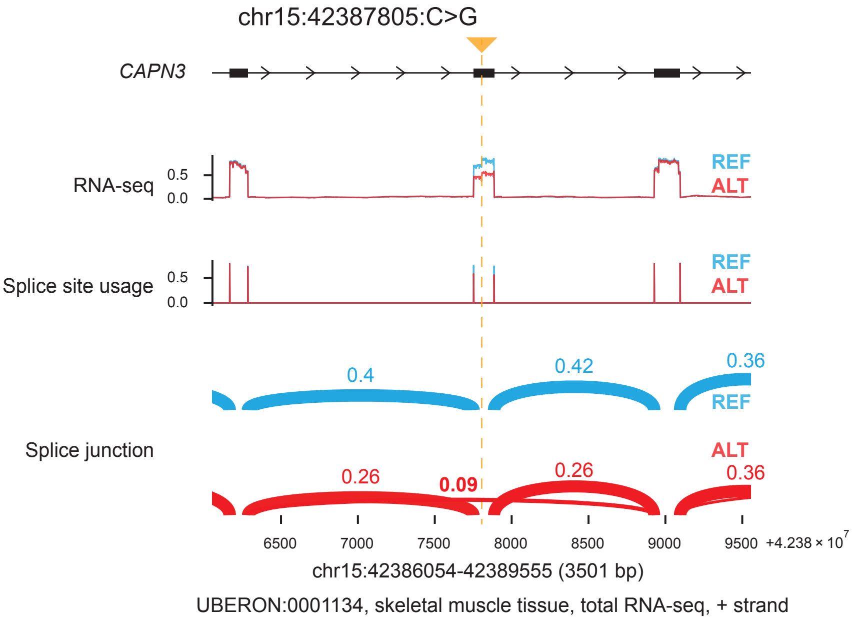

Supplementary Figure 16 | **AlphaGenome predictions for variant `chr15:42387805:C>G` in skeletal muscle tissue**. RNA-seq, splice site usage, and splice junction track predictions are shown for the reference and alternate allele.

amino acid (missense) levels.

Conclusion: *In silico* evidence for variant effect

The combined *in silico* evidence from AlphaGenome and a correctly parameterized SpliceAI supports a deleterious splicing effect. This would justify activating the ACMG code PP3 ("Multiple lines of computational evidence support a deleterious effect"). However, the curators had already activated PS3 ("*In vitro*... functional studies supportive of a damaging effect") based on the minigene assay results. Since ACMG guidelines allow only one of the two evidence codes to be activated, our findings support the existing evidence but would not change the variant's final classification.

Supplementary References

68. Mungall, C. J., Torniai, C., Gkoutos, G. V., Lewis, S. E. & Haendel, M. A. Uberon, an integrative multi-species anatomy ontology. *Genome Biol.* **13** (2012). Cited on page 2.
69. Malone, J. *et al.* Modeling sample variables with an Experimental Factor Ontology. *Bioinformatics* **26** (2010). Cited on page 2.
70. Sarntivijai, S. *et al.* CLO: The cell line ontology. *J. Biomed. Semantics* **5** (2014). Cited on page 2.
71. Diehl, A. D. *et al.* The Cell Ontology 2016: enhanced content, modularization, and ontology interoperability. *J. Biomed. Semantics* **7** (2016). Cited on page 2.
72. ENCODE Project Consortium. An integrated encyclopedia of DNA elements in the human genome. *Nature* **489** (2012). Cited on pages 2, 3, 34.
73. Lizio, M. *et al.* Gateways to the FANTOM5 promoter level mammalian expression atlas. *Genome Biol.* **16** (2015). Cited on pages 2, 4.
74. Reiff, S. B. *et al.* The 4D Nucleome Data Portal as a resource for searching and visualizing curated nucleomics data. *Nat Commun* **13** (2022). Cited on page 3.
75. Wilks, C. *et al.* recount3: summaries and queries for large-scale RNA-seq expression and splicing. *Genome Biol.* **22** (2021). Cited on page 4.
76. Noguchi, S. *et al.* FANTOM5 CAGE profiles of human and mouse samples. *Sci. Data* **4** (2017). Cited on page 4.
77. Dobin, A. *et al.* STAR: Ultrafast universal RNA-seq aligner. *Bioinformatics* **29** (2013). Cited on page 6.
78. Danecek, P. *et al.* Twelve years of SAMtools and BCFtools. *Gigascience* **10** (2021). Cited on page 6.
79. Dent, C. I. *et al.* Quantifying splice-site usage: a simple yet powerful approach to analyze splicing. *NAR Genom. Bioinform.* **3** (2021). Cited on page 7.
80. Fudenberg, G., Kelley, D. R. & Pollard, K. S. Predicting 3D genome folding from DNA sequence with Akita. *Nat. Methods* **17** (2020). Cited on page 8.
81. Bradbury, J. *et al.* *JAX: composable transformations of Python+NumPy programs* 2018. `http://github.com/jax-ml/jax`. Cited on page 9.
82. Hennigan, T., Cai, T., Norman, T., Martens, L. & Babuschkin, I. *Haiku: Sonnet for JAX* 2020. `https://github.com/google-deepmind/dm-haiku`. Cited on page 9.
83. Ioffe, S. & Szegedy, C. *Batch normalization: Accelerating deep network training by reducing internal covariate shift* in *Proc. International Conference on Machine Learning (ICML)* (2015). Cited on page 10.
84. Hendrycks, D. & Gimpel, K. *Gaussian Error Linear Units (GELUs)* Preprint at `https://arxiv.org/abs/1606.08415`. 2016. Cited on page 10.
85. Brock, A., De, S. & Smith, S. L. *Characterizing signal propagation to close the performance gap in unnormalized ResNets* in *Proc. International Conference on Machine Learning (ICML)* (2020). Cited on page 10.
86. De, S. & Smith, S. Batch Normalization Biases Residual Blocks Towards the Identity Function in Deep Networks. *Advances in Neural Information Processing Systems* **33** (2020). Cited on page 10.
87. Jumper, J. *et al.* Highly accurate protein structure prediction with AlphaFold. *Nature* **596** (2021). Cited on page 12.
88. Shazeer, N. *Fast transformer decoding: One write-head is all you need* Preprint at `https://arxiv.org/abs/1911.02150`. 2019. Cited on page 12.
89. Bello, I., Pham, H., Le, Q. V., Norouzi, M. & Bengio, S. *Neural Combinatorial Optimization with Reinforcement Learning* Preprint at `https://arxiv.org/abs/1611.09940`. 2016. Cited on page 12.
90. RoFormer: Enhanced transformer with Rotary Position Embedding. *Neurocomputing* **568** (2024). Cited on page 12.
91. Dai, Z. *et al.* *Transformer-XL: Attentive Language Models Beyond a Fixed-Length Context* Preprint at `https://arxiv.org/abs/1901.02860`. 2019. Cited on page 13.
92. Loshchilov, I. & Hutter, F. *Decoupled Weight Decay Regularization* Preprint at `https://arxiv.org/abs/1711.05101`. 2017. Cited on page 20.

93. Jeanteur, P. *Alternative Splicing and Disease* (Springer Science & Business Media, 2006). Cited on page 21.
94. Chao, K.-H., Mao, A., Salzberg, S. L. & Pertea, M. Splam: a deep-learning-based splice site predictor that improves spliced alignments. *Genome Biol* **25** (2024). Cited on page 21.
95. Wang, R., Nambiar, R., Zheng, D. & Tian, B. PolyA_DB 3 catalogs cleavage and polyadenylation sites identified by deep sequencing in multiple genomes. *Nucleic Acids Res* **46** (2018). Cited on page 22.
96. Kerimov, N. *et al.* A compendium of uniformly processed human gene expression and splicing quantitative trait loci. *Nat. Genet.* **53** (2021). Cited on pages 30, 36.
97. Scheller, I. F., Lutz, K., Mertes, C., Yépez, V. A. & Gagneur, J. Improved detection of aberrant splicing with FRASER 2.0 and the intron Jaccard index. *Am. J. Hum. Genet.* **110** (2023). Cited on page 31.
98. Yépez, V. A. *et al.* Detection of aberrant gene expression events in RNA sequencing data. *Nat. Protoc.* **16** (2021). Cited on page 31.
99. Koenig, Z. *et al.* A harmonized public resource of deeply sequenced diverse human genomes. *Genome Res* **34** (2024). Cited on page 31.
100. Nori, H., Jenkins, S., Koch, P. & Caruana, R. *InterpretML: A Unified Framework for Machine Learning Interpretability* Preprint at `https://arxiv.org/abs/1909.09223`. 2019. Cited on page 31.
101. Kerimov, N. *et al.* eQTL Catalogue 2023: New datasets, X chromosome QTLs, and improved detection and visualisation of transcript-level QTLs. *PLoS Genet* **19** (2023). Cited on pages 32, 33.
102. Pedregosa, F. *et al.* Scikit-learn: Machine Learning in Python. *Journal of Machine Learning Research* **12** (2011). Cited on pages 33, 38.
103. Roadmap Epigenomics Consortium *et al.* Integrative analysis of 111 reference human epigenomes. *Nature* **518** (2015). Cited on page 34.
104. Luo, Y. *et al.* New developments on the Encyclopedia of DNA Elements (ENCODE) data portal. *Nucleic Acids Res.* **48** (2020). Cited on page 34.
105. Fulco, C. P. *et al.* Activity-by-contact model of enhancer–promoter regulation from thousands of CRISPR perturbations. *Nature genetics* **51** (2019). Cited on page 34.
106. Nasser, J. *et al.* Genome-wide enhancer maps link risk variants to disease genes. *Nature* **593** (2021). Cited on page 34.
107. McLaren, W. *et al.* The Ensembl Variant Effect Predictor. *Genome Biol.* **17** (2016). Cited on pages 34, 37.
108. Koscielny, G. *et al.* Open Targets: a platform for therapeutic target identification and validation. *Nucleic acids research* **45** (2017). Cited on pages 37, 61.
109. Merico, D. *et al.* ATP7B variant c. 1934T> G p. Met645Arg causes Wilson disease by promoting exon 6 skipping. *NPJ genomic medicine* **5** (2020). Cited on page 56.
110. Nishimura, D. Y. *et al.* Positional cloning of a novel gene on chromosome 16q causing Bardet–Biedl syndrome (BBS2). *Human Molecular Genetics* **10** (2001). Cited on page 56.
111. Vargas-Poussou, R. *et al.* Spectrum of mutations in Gitelman syndrome. *Journal of the American Society of Nephrology* **22** (2011). Cited on page 56.
112. Cummings, B. B. *et al.* Improving genetic diagnosis in Mendelian disease with transcriptome sequencing. *Science translational medicine* **9** (2017). Cited on page 56.
113. Bolduc, V. *et al.* A recurrent COL6A1 pseudoexon insertion causes muscular dystrophy and is effectively targeted by splice-correction therapies. *JCI insight* **4** (2019). Cited on page 56.
114. Gillis, E. *et al.* An FBN 1 Deep Intronic Mutation in a Familial Case of Marfan Syndrome: An Explanation for Genetically Unsolved Cases? *Human Mutation* **35** (2014). Cited on page 56.
115. Kazazian, H. J., Dowling, C. E., Waber, P. G., Huang, S. & Lo, W. The spectrum of beta-thalassemia genes in China and Southeast Asia (1986). Cited on page 56.
116. Faghfoury, H., Baruteau, J., Ogier de Baulny, H., Häberle, J. & Schulze, A. Transient fulminant liver failure as an initial presentation in citrullinemia type I. *Molecular Genetics and Metabolism* **102** (2011). Cited on page 56.
117. Ellingford, J. M., Ahn, J. W., Bagnall, R. D., *et al.* Recommendations for clinical interpretation of variants found in non-coding regions of the genome. *Genome Medicine* **14** (2022). Cited on page 66.

118. Martin-Geary, A. C., Lecoquierre, F., Walker, S., Whiffin, N. & Dawes, R. *Using SpliceAI to triage splice-altering variants in 7220 individuals with rare conditions highlights limitations of the precomputed scores* Preprint at https://www.medrxiv.org/content/10.1101/2025.08.27.25334471v1. 2025. Cited on page 71.
