# Supplementary material for: Advancing regulatory variant effect prediction with AlphaGenome
Source: Nature. 2026 Jan 28;649(8099):1206–18. doi: 10.1038/s41586-025-10014-0 (PMC12851941; doi:10.1038/s41586-025-10014-0)
Supplement: Supplementary file 2 — Reporting Summary [file 41586_2025_10014_MOESM2_ESM.pdf]

nature portfolio

Corresponding author(s): Ziga Avsec & Pushmeet Kohli

Last updated by author(s): Nov 25, 2025

Reporting Summary

Nature Portfolio wishes to improve the reproducibility of the work that we publish. This form provides structure for consistency and transparency in reporting. For further information on Nature Portfolio policies, see our Editorial Policies and the Editorial Policy Checklist.

Statistics

For all statistical analyses, confirm that the following items are present in the figure legend, table legend, main text, or Methods section.

n/a	Confirmed	
☐	☒	The exact sample size (*n*) for each experimental group/condition, given as a discrete number and unit of measurement
☒	☐	A statement on whether measurements were taken from distinct samples or whether the same sample was measured repeatedly
☐	☒	The statistical test(s) used AND whether they are one- or two-sided<br>*Only common tests should be described solely by name; describe more complex techniques in the Methods section.*
☒	☐	A description of all covariates tested
☒	☐	A description of any assumptions or corrections, such as tests of normality and adjustment for multiple comparisons
☐	☒	A full description of the statistical parameters including central tendency (e.g. means) or other basic estimates (e.g. regression coefficient) AND variation (e.g. standard deviation) or associated estimates of uncertainty (e.g. confidence intervals)
☐	☒	For null hypothesis testing, the test statistic (e.g. *F*, *t*, *r*) with confidence intervals, effect sizes, degrees of freedom and *P* value noted<br>*Give P values as exact values whenever suitable.*
☒	☐	For Bayesian analysis, information on the choice of priors and Markov chain Monte Carlo settings
☒	☐	For hierarchical and complex designs, identification of the appropriate level for tests and full reporting of outcomes
☐	☒	Estimates of effect sizes (e.g. Cohen's *d*, Pearson's *r*), indicating how they were calculated

Our web collection on statistics for biologists contains articles on many of the points above.

Software and code

Policy information about availability of computer code

Data collection	No software was used for data collection. This study utilized pre-existing, publicly available datasets.
Data analysis	Data analysis used Python v.3.11.8 (https://www.python.org/), NumPy v2.2.5 (https://github.com/numpy/numpy), SciPy v.1.14.1 (https://www.scipy.org/), seaborn v.0.12.2 (https://github.com/mwaskom/seaborn), Matplotlib v.3.9.1 (https://github.com/matplotlib/matplotlib), Pandas v.2.2.3 (https://github.com/pandas-dev/pandas), anndata v0.11.4 (https://github.com/scverse/anndata), Scikit-learn v1.6.1, (https://github.com/scikit-learn/scikit-learn), STAR (version 2.7.11b), Samtools (version 1.21), FRASER 2.085, DROP pipeline (version 1.3.3), and Colab (https://research.google.com/colaboratory; release 2025-06-16).<br><br>AlphaGenome is available for non-commercial use via an online API at http://deepmind.google.com/science/alphagenome, with an accompanying Python software development kit (SDK) provided to interact with the model. The model source code and weights will also be provided upon final publication.We also provide a genome interpretation suite to facilitate the exploration and interpretation of AlphaGenome. In addition to the online API and interpretation suite, we also provide model source code, weights, variant scoring implementations and a selection of variant evaluation datasets and predictions. These can all be found at https://github.com/google-deepmind/alphagenome

For manuscripts utilizing custom algorithms or software that are central to the research but not yet described in published literature, software must be made available to editors and reviewers. We strongly encourage code deposition in a community repository (e.g. GitHub). See the Nature Portfolio guidelines for submitting code & software for further information.

Data

Policy information about availability of data

All manuscripts must include a data availability statement. This statement should provide the following information, where applicable:

- Accession codes, unique identifiers, or web links for publicly available datasets
- A description of any restrictions on data availability
- For clinical datasets or third party data, please ensure that the statement adheres to our policy

All primary experimental datasets utilized for the training and evaluation of AlphaGenome in this study were obtained from publicly accessible sources. A comprehensive manifest detailing these data sources – including specific repositories (e.g., ENCODE portal, GTEx portal, 4D Nucleome portal, ClinVar, gnomAD), individual accession numbers, relevant version information, and direct URLs where applicable – is provided in Supplementary Table 2. This study did not generate new primary experimental data requiring deposition.

Research involving human participants, their data, or biological material

Policy information about studies with human participants or human data. See also policy information about sex, gender (identity/presentation), and sexual orientation and race, ethnicity and racism.

Reporting on sex and gender	No human population data was collected as part of this study. Sex is included as clinical covariates for the eQTL mapping. We also report sex-specific track data.
Reporting on race, ethnicity, or other socially relevant groupings	There is a description of genetic ancestry in QTL tasks - Ancestries mentioned in the text are European, African, and Yoruba.
Population characteristics	No human population data was collected as part of this study.
Recruitment	No human data was collected as part of this study.
Ethics oversight	No human data was collected as part of this study.

Note that full information on the approval of the study protocol must also be provided in the manuscript.

Field-specific reporting

Please select the one below that is the best fit for your research. If you are not sure, read the appropriate sections before making your selection.

☒ Life sciences ☐ Behavioural & social sciences ☐ Ecological, evolutionary & environmental sciences

For a reference copy of the document with all sections, see nature.com/documents/nr-reporting-summary-flat.pdf

Life sciences study design

All studies must disclose on these points even when the disclosure is negative.

Sample size	All available data were used for each benchmark and evaluation. No subsampling was performed
Data exclusions	Data were binned into splits during model development. No data were excluded from benchmarks.
Replication	Code and methods were carefully checked for completeness and replicability.
Randomization	Random assignment of subjects was not applicable as this is a computational study utilizing pre-existing, publicly available genomic datasets. However, randomness was employed computationally via random seeds for model initialization and data augmentation. To ensure robust performance estimation and prevent data leakage, data was partitioned using pre-defined genomic interval splits (following established benchmarks) for model training and evaluation.
Blinding	Blinding was not necessary as the evaluation relies on automated, quantitative benchmarking rather than subjective scoring. Bias was mitigated by strictly segregating training, validation, and testing data to ensure models were evaluated solely on data they had never seen.

Reporting for specific materials, systems and methods

We require information from authors about some types of materials, experimental systems and methods used in many studies. Here, indicate whether each material, system or method listed is relevant to your study. If you are not sure if a list item applies to your research, read the appropriate section before selecting a response.

Materials & experimental systems

n/a	Involved in the study
☒	☐ Antibodies
☒	☐ Eukaryotic cell lines
☒	☐ Palaeontology and archaeology
☒	☐ Animals and other organisms
☒	☐ Clinical data
☒	☐ Dual use research of concern
☒	☐ Plants

Methods

n/a	Involved in the study
☒	☐ ChIP-seq
☒	☐ Flow cytometry
☒	☐ MRI-based neuroimaging

Plants

Seed stocks	None
Novel plant genotypes	None
Authentication	None
